# 8-Hydroxyquinoline-Amino Acid Hybrids and Their Half-Sandwich Rh and Ru Complexes: Synthesis, Anticancer Activities, Solution Chemistry and Interaction with Biomolecules †

**DOI:** 10.3390/ijms222011281

**Published:** 2021-10-19

**Authors:** Tamás Pivarcsik, Orsolya Dömötör, János P. Mészáros, Nóra V. May, Gabriella Spengler, Oszkár Csuvik, István Szatmári, Éva A. Enyedy

**Affiliations:** 1 MTA-SZTE Lendület Functional Metal Complexes Research Group, University of Szeged, Dóm Tér 7, H-6720 Szeged, Hungary; pivarcsik.tamas@chem.u-szeged.hu (T.P.); domotor.o@chem.u-szeged.hu (O.D.); meszaros.janos@chem.u-szeged.hu (J.P.M.); spengler.gabriella@med.u-szeged.hu (G.S.); 2 Department of Inorganic and Analytical Chemistry, Interdisciplinary Excellence Centre, University of Szeged, Dóm Tér 7, H-6720 Szeged, Hungary; 3 Centre for Structural Science, Research Centre for Natural Sciences, Magyar Tudósok Körútja 2, H-1117 Budapest, Hungary; may.nora@ttk.hu; 4 Department of Medical Microbiology, Albert Szent-Györgyi Health Center and Albert Szent-Györgyi Medical School, University of Szeged, Semmelweis U. 6, H-6725 Szeged, Hungary; 5 Institute of Pharmaceutical Chemistry and Stereochemistry Research Group of Hungarian Academy of Sciences, University of Szeged, Eötvös U. 6, H-6720 Szeged, Hungary; oszkar.csuvik@pharm.u-szeged.hu (O.C.); szatmari.istvan@szte.hu (I.S.)

**Keywords:** multidrug resistance, solution speciation, cytotoxicity, organometallic, DNA binding, albumin binding

## Abstract

Solution chemical properties of two novel 8-hydroxyquinoline-D-proline and homo-proline hybrids were investigated along with their complex formation with [Rh(η^5^-C_5_Me_5_)(H_2_O)_3_]^2+^ and [Ru(η^6^-*p*-cymene)(H_2_O)_3_]^2+^ ions by pH-potentiometry, UV-visible spectrophotometry and ^1^H NMR spectroscopy. Due to the zwitterionic structure of the ligands, they possess excellent water solubility as well as their complexes. The complexes exhibit high solution stability in a wide pH range; no significant dissociation occurs at physiological pH. The hybrids and their Rh(η^5^-C_5_Me_5_) complexes displayed enhanced cytotoxicity in human colon adenocarcinoma cell lines and exhibited multidrug resistance selectivity. In addition, the Rh(η^5^-C_5_Me_5_) complexes showed increased selectivity to the chemosensitive cancer cells over the normal cells; meanwhile, the Ru(η^6^-*p*-cymene) complexes were inactive, most likely due to arene loss. Interaction of the complexes with human serum albumin (HSA) and calf-thymus DNA (ct-DNA) was investigated by capillary electrophoresis, fluorometry and circular dichroism. The complexes are able to bind strongly to HSA and ct-DNA, but DNA cleavage was not observed. Changing the five-membered proline ring to the six-membered homoproline resulted in increased lipophilicity and cytotoxicity of the Rh(η^5^-C_5_Me_5_) complexes while changing the configuration (L vs. D) rather has an impact on HSA or ct-DNA binding.

## 1. Introduction

Half-sandwich organometallic compounds are among the most widely studied anticancer complexes of platinum group metals such as Ru, Rh, Os, and Ir [1,2]. The physico-chemical and biological properties of the half-sandwich complexes can be fine-tuned by varying the metal center, the π-bound ligand (usually arene or cyclopentadienyl derivative ring) and the facial ligands occupying the remaining coordination sites [1,2,3,4]. The piano-stool complexes often contain a bidentate ligand and a chlorido co-ligand as leaving group, and these components affect the solution stability, lipophilicity, the overall charge and the reactivity of the complexes; thus, they also have a strong influence on the anticancer properties [1,2,3,4,5,6,7,8]. The bidentate ligands are typically (N,N), (N,O), (O,S) or (O,O) donor chelating compounds and can possess intrinsic cytotoxicity. Among the bioactive ligands, 8-hydroxyquinoline (HQ) and its derivatives are extensively studied [7,8,9,10]. The HQ scaffold is used as a privileged structure due to its broad range of pharmacological properties [10,11,12,13,14,15,16,17,18,19], which are often related to the ability to coordinate to endogenous metal ions such as Fe(II/III), Cu(II) and Zn(II) [10,13,14,15,16]. Numerous cytotoxic complexes of HQ ligands formed with rare earth metal ions [20], Pd(II) [21] or Pt(II) [22] are also reported. One of the most prominent compounds is the Ga(III) complex of HQ (tris(8-quinolinolato)gallium, KP46), which had successfully entered clinical trials and it was effective against renal cancer [23]. HQ and its derivatives also form complexes with Ru(η^6^-*p*-cymene) (RuCym) and Rh(η^5^-C_5_Me_5_) (RhCp*) characterized by high solution stability, and many of them were reported to display potent anticancer activity in human cancer cell lines [7,8,9,24,25,26,27].

**Scheme 1:**
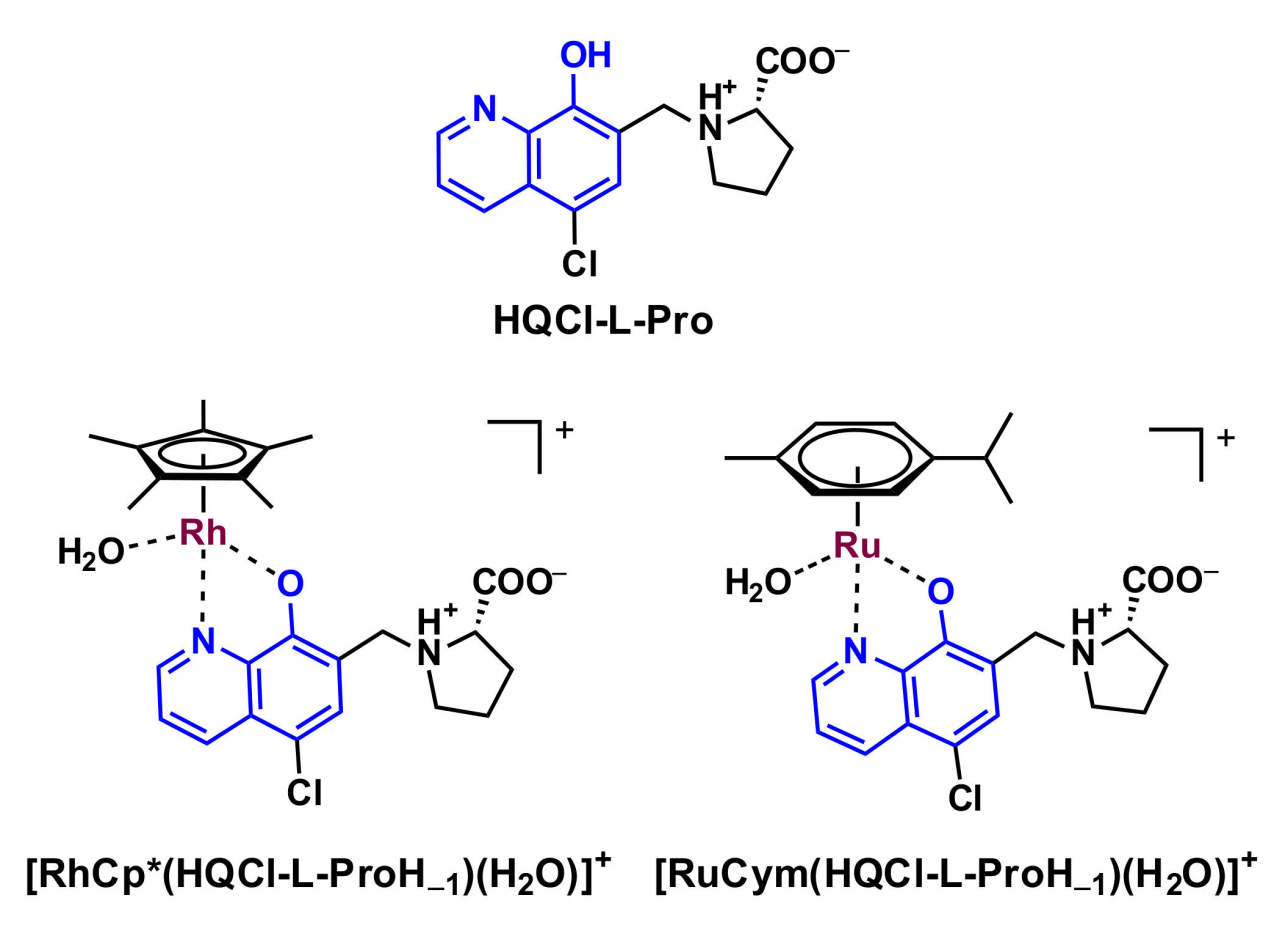
Chemical formula of HQCl-L-Pro in its neutral form and its RuCym and RhCp* aquated complexes [25].

Organometallic complexes of a series of halogenated 8-hydroxyquinoline derivatives were developed and studied thoroughly by the research groups of Turel and Hartinger [7,9,26,27,28,29]. However, some of these half-sandwich organometallic complexes were found to be fairly promising; they generally suffer from poor water solubility due to their lipophilic nature. There are some options to improve the solubility of the complexes, namely more hydrophilic substituents on the arene and/or on the HQ ligands that can be utilized, or the leaving group can be modified. Movassaghi et al. introduced a more polar aromatic ligand (*N*-acetyl-L-phenylalanine ethyl ester) instead of *p*-cymene in Ru(II) complexes, which resulted in a positive effect on the solubility while the cytotoxicity remained at the low-micromolar level [28]. The solubility could be increased when the hydrophilic 1,3,5-triaza-7-phosphaadamantane (PTA) was introduced as a co-ligand; however, the cytotoxicity was decreased [26]. When a polar functional group (SO_3_^−^) is introduced in the case of the 8-hydroxyquinoline-5-sulfonate, the complexes possess excellent water solubility, although it caused the loss of the anticancer activity reported in our previous study [24]. Therefore, the lipophilic–hydrophilic characteristics of the complexes should also be optimized very carefully for adequate solubility and anticancer properties. In our previous work, we developed an HQ–L-proline hybrid (S)-5-chloro-7-((proline-1-yl)methyl)8-hydroxyquinoline (HQCl-L-Pro, Scheme 1) with increased water solubility due to its zwitterionic structure (COO^−^ and NH^+^ moieties) in a wide pH range including the physiological pH; its RuCym and RhCp* complexes (Scheme 1) were also relatively hydrophilic [25].

HQCl-L-Pro and its RhCp* complex were found to be somewhat more cytotoxic against doxorubicin-resistant Colo 320 adenocarcinoma human cells compared to the drug-sensitive Colo-205 cells. On the other hand, complexation with RuCym resulted in lower cytotoxic activity, possibly due to arene loss and less efficient cellular uptake [25]. The paradoxical toxicity of certain HQ derivatives against P-glycoprotein (P-gp)-expressing multidrug-resistant cells was already reported earlier [30,31]. The preferential toxicity in otherwise multidrug-resistant cell lines is hypothesized to be due to the incorporation of the CH_2_-N subunit at position seven on the HQ scaffold [30]. It is noteworthy that multidrug resistance (MDR) is one of the major impediments to successful cancer treatment. 

**Scheme 2:**
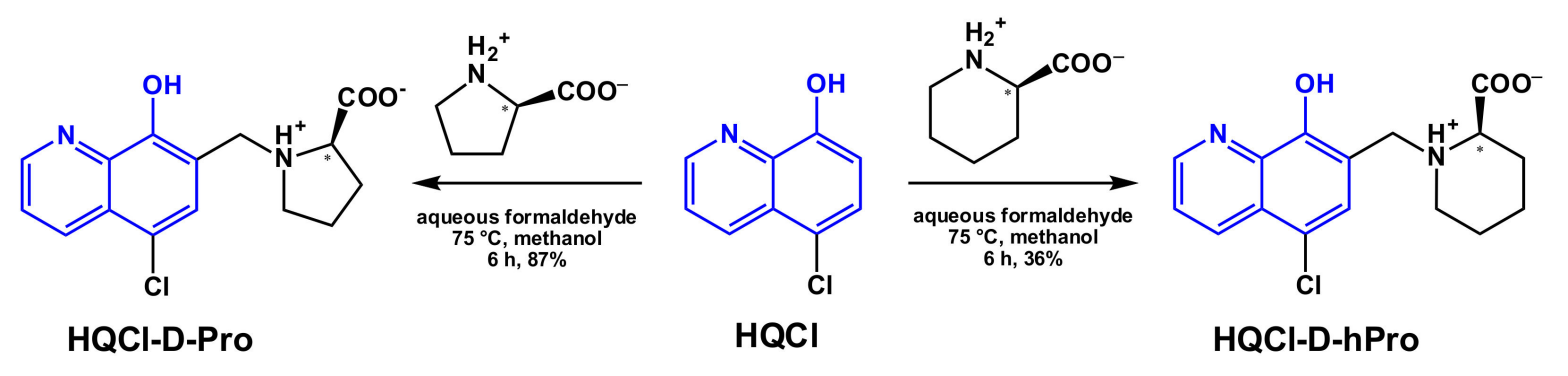
Synthetic route for the HQCl-D-Pro and HQCl-D-hPro ligands obtained from HQCl. (The symbol * denotes the chirality center.)

In this work, we designed two novel HQ–D-amino acid hybrids retaining the MDR-selectivity and the excellent water solubility, namely (R)-5-chloro-7-((proline-1-yl)methyl)8-hydroxyquinoline (HQCl-D-Pro) and (R)-5-chloro-7-((homoproline-1-yl)methyl)8-hydroxyquinoline (HQCl-D-hPro) were developed (Scheme 2). Our aim was to monitor the effect of the chirality of the amino acid moiety (L vs. D) and the substitution of proline fragment with homoproline on the cytotoxicity, MDR-selectivity, lipophilicity and complex formation ability. Herein, we report the synthesis and characterization of HQCl-D-Pro, HQCl-D-hPro and their RuCym and RhCp* complexes. Solution stability, structure, p*K*_a_ values for deprotonation of the aqua ligand, chloride ion affinity and lipophilicity of the complexes were determined in addition to the investigation of their cytotoxicity in chemosensitive and in multidrug-resistant cancer cells. Interaction with human serum albumin (HSA) and DNA was also investigated since plasma protein binding is an important factor of ADME profiling, while DNA might be considered as a potential target molecule for complexes of platinum group metals. 

## 2. Results and Discussion

### 2.1. Synthesis of HQCl-D-Pro and HQCl-D-hPro, Their Ru(η^6^-p-cymene) and Rh(η^5^-C_5_Me_5_) Complexes 

In order to increase the water solubility of 5-chloro-8-hydroxyquinoline (HQCl), zwitterionic D-proline and D-homoproline moieties were incorporated in the ligand scaffold. The coupling of HQCl with the D-amino acids was achieved via a modified Mannich reaction (Scheme 2). HQCl, (homo)proline and formaldehyde were refluxed in methanol for 6 h. The quantity of HQCl was 1.2 equivalent to (homo)proline since the elimination of the non-reacted or incompletely reacted HQCl is more convenient than the elimination of D-amino acids. The structures of both novel compounds were confirmed by ^1^H and ^13^C NMR spectroscopic and electrospray mass spectrometry (ESI-MS) measurements (see Experimental section and NMR spectra in Figures S1–S4).

**Scheme 3:**
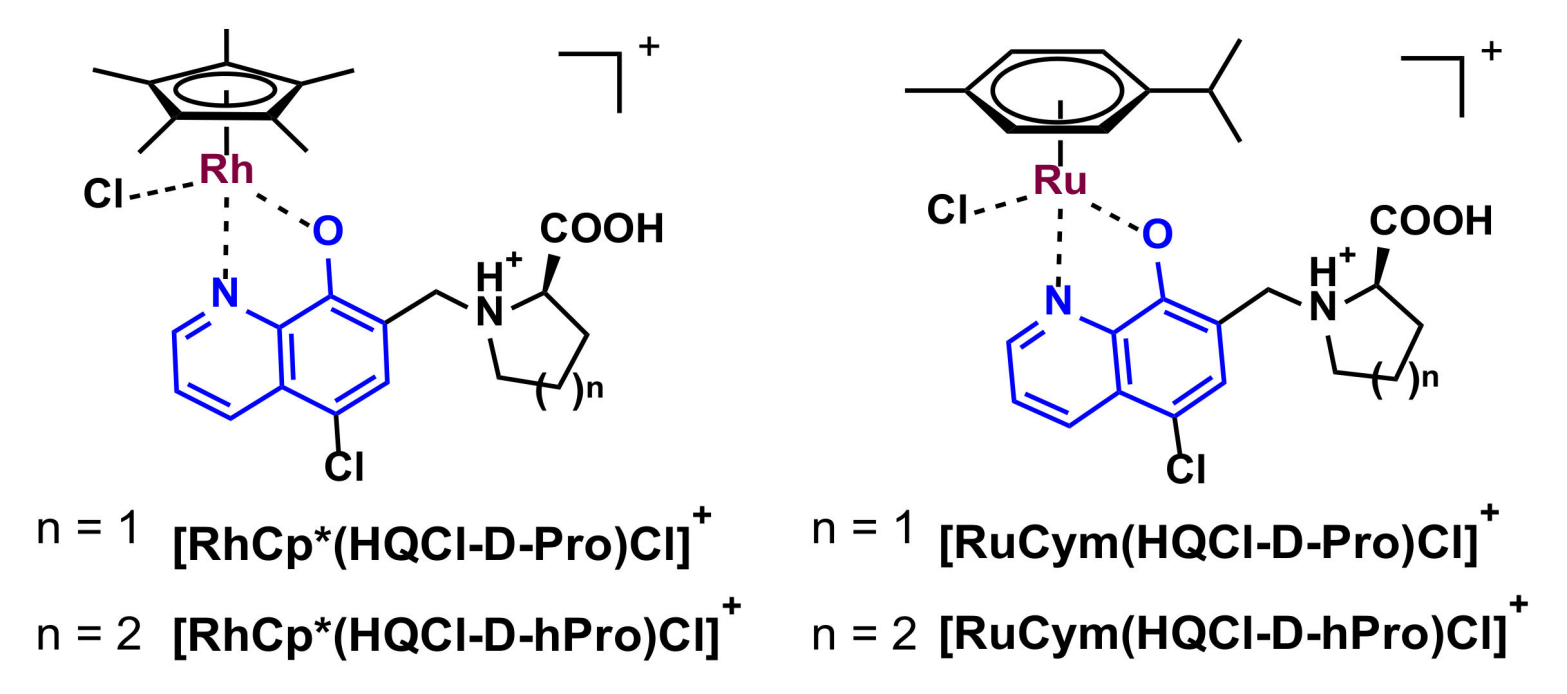
Chemical structure of the isolated RhCp* and RuCym complexes of the 8-hydroxyquinoline-amino acid hybrids (counter ion: Cl^−^). (The possible H bond between the N_(h)Pro_H^+^ and coordinated O^−^ moieties is not shown. The symbol n indicates the number of carbon atoms: 1 or 2.)

The synthesis and isolation of the organometallic RuCym and RhCp* complexes of HQCl-D-Pro and HQCl-D-hPro reaction (Scheme 3) were performed with the well-established method; namely, the ligands were mixed with half-equivalent of the corresponding dimeric precursor [M(arene)Cl_2_]_2_ in methanol. After 24 h reaction time, the solvent was partly evaporated, and precipitation was completed with the addition of diethyl ether. The formed complexes were decanted, filtered out and washed with diethyl ether and *n*-hexane, then dried. The structure and purity of the four chlorido [M(arene)(HL)Cl]Cl (Scheme 3) complexes were characterized by ^1^H and ^13^C NMR spectroscopy in CD_3_OD, ESI-MS (see Experimental section and Figures S5–S12) and by X-ray crystallography for the RhCp*–HQCl-D-hPro complex (see the next section). Additionally, capillary zone electrophoresis (CZE) was also applied to check the purity of the complexes (an exemplary electropherogram is shown in Figure S13). Both the NMR spectra and the electropherograms indicate the formation of the complexes and the lack of the peaks belonging to the unbound ligands and metal precursors.

### 2.2. Structural Studies of [RhCp*(HQCl-D-hPro)Cl]Cl∙H_2_O∙CH_3_OH (1) by X-ray Crystallography

**Figure 1:**
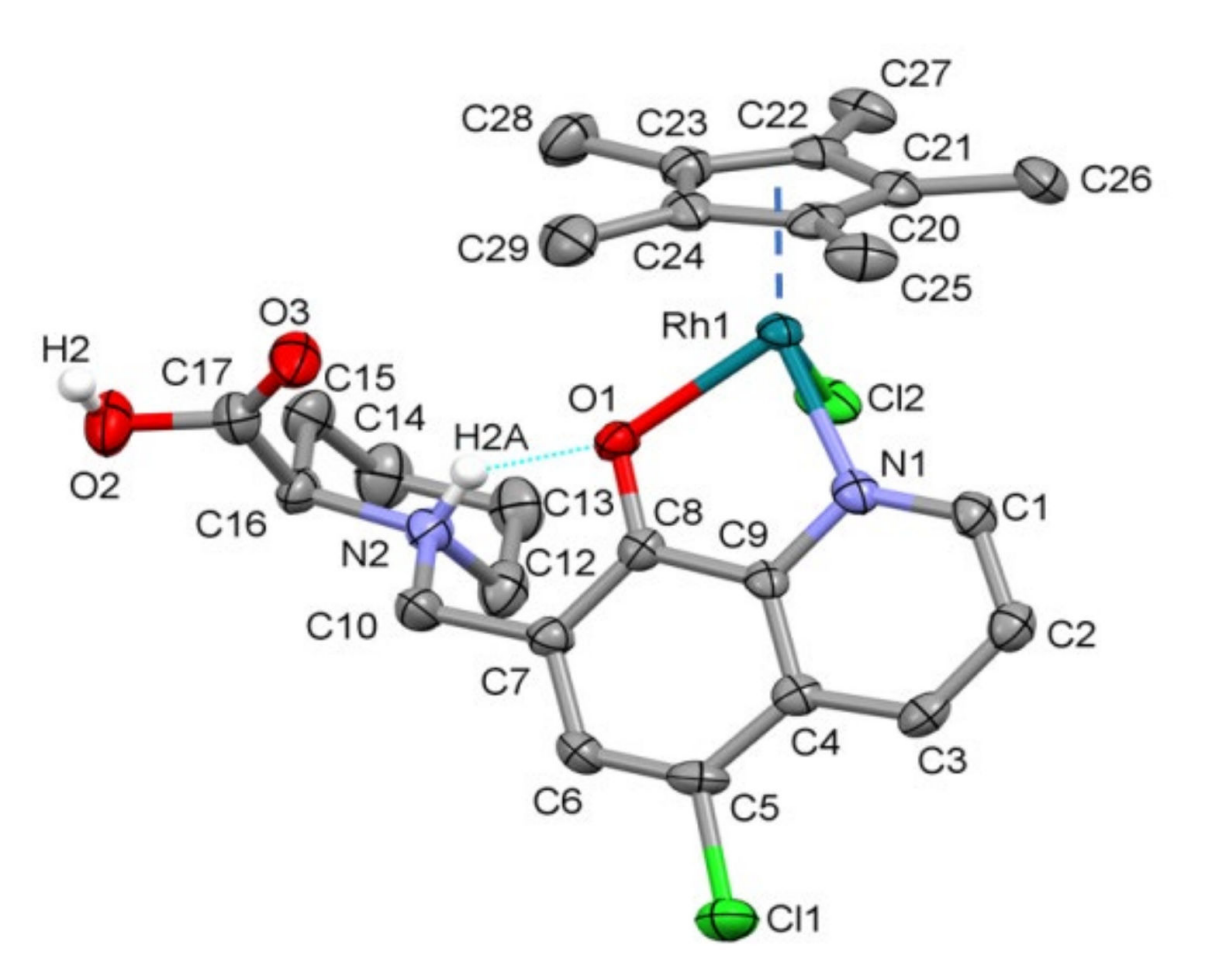
Molecular structure of [RhCp*(HQCl-D-hPro)Cl]Cl∙H_2_O∙CH_3_OH (**1**) with atom numbering. Displacement parameters are drawn at 50% probability level. Solvents, counter ion and hydrogen atoms are omitted for clarity (except homoproline proton H2A involved in intramolecular hydrogen bond (light blue solid line) and the proton on the carboxyl group).

For the crystallization of [RhCp*(HQCl-D-hPro)Cl]Cl, the vapor diffusion method was applied; accordingly, the previously isolated complex was dissolved in methanol, and diethyl ether was allowed to diffuse into the sample slowly. The crystal structure of (**1**) was determined by single-crystal X-ray diffraction (Table S1). The complex crystallized in monoclinic crystal system in the *P*2_1_ space group. The ORTEP representation of the compound is shown in Figure 1, and the unit cell containing two molecules can be seen in Figure S14.

In this crystal, the RhCp* ion is coordinated by quinoline nitrogen and hydroxylate oxygen, as was expected, while the COOH group and homoproline nitrogen are protonated. The coordination sphere is saturated by the binding of a chlorido co-ligand, and the overall charge of the complex (+1) is neutralized by a Cl^−^ counter ion. The asymmetrical unit contains methanol and a water molecule; the latter has two disordered positions with an occupancy ratio of 70/30. The water hydrogen atoms could not be detected on the difference Fourier map. The dihedral angle calculated between the Cp* and 8-hydroxyquinoline ring planes is 57.6°, and between the 8-hydroxyquinoline and the homoproline ring plane, it is 72.5°. The latter angle is probably due to the intramolecular hydrogen bond that emerged between the protonated homoproline nitrogen and the coordinated O^−^ atom (N2-H2A…O1), which further stabilizes the coordination of the ligand. Some selected bond lengths and angles are collected in Table S2. The main secondary interaction between the molecules is the C/O–H…O/Cl hydrogen bonds. Selected interatomic distances and angles are collected in Table S3, and the main hydrogen bonds are shown in Figure S15. No significant π–π interaction could be found between the aromatic rings of the molecules. The closest Rh–Rh distance is 7.688(6) Å in the crystal. Comparison of the coordination geometry in crystal (**1**) with previously determined [RhCp*)(8-hydroxyquinolinato)Cl] (Ref. code GAQLES) [24] with the overlay of Rh and the coordinating Cl, O and N atoms is shown in Figure S16. The dihedral angle between Cp*—8-hydroxyquinoline ring plane is 49.0(3)° in the case of GAQLES, while it is 57.6° for (**1**). The Rh–Cg distance is somewhat shorter in crystal (**1**) (1.758(3) Å) than in GAQLES (1.768(3) Å).

### 2.3. In Vitro Cytotoxicity and Induction of Apoptosis of the Ligands and Their Complexes

The in vitro cytotoxic activity of the novel water-soluble amino acid derivatives of HQCl and their RhCp* and RuCym complexes was measured by the thiazolyl blue tetrazolium bromide (MTT) assay in two human colon adenocarcinoma cell lines (Colo-205 and Colo-320) and in one non-tumoral human lung fibroblast cell line (MRC-5). The results are given in terms of IC_50_ values (Table 1). Colo-205 is a drug-sensitive cell line with a normal ABCB1 transporter expression pattern, while Colo-320 is multidrug-resistant and overexpresses the ABCB1 system. 

**Figure 2:**
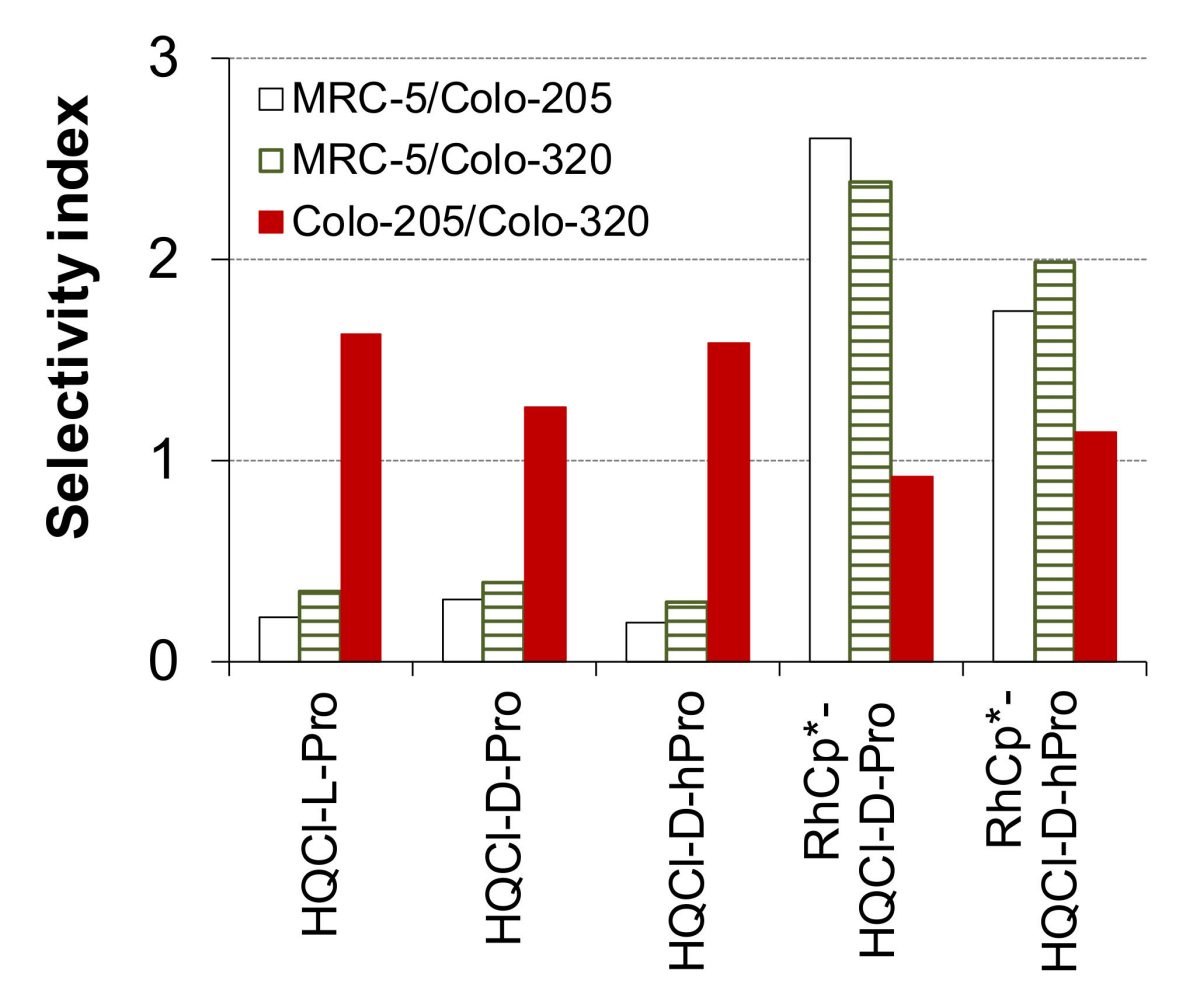
Selectivity indexes (ratio of the IC_50_ values measured for Colo-205, Colo-320 and MRC-5 cells) calculated between the indicated cell lines for the ligands (HQCl-L-Pro, HQCl-D-Pro, HQCl-D-hPro) and the RhCp* complex of HQCl-D-Pro and HQCl-D-hPro. (72 h incubation time.)

Doxorubicin was used as a positive control, and HQCl-L-Pro as the enantiomeric pair of HQCl-D-Pro was also assayed for comparison. Different selectivity indexes were calculated as the quotient of the IC_50_ values in MRC-5, and the given cancer cell or the IC_50_ value obtained in the sensitive cell was divided by that of the resistant cell (Figure 2). The organometallic precursors have no toxic effect in these cell lines [25]. The tested amino acid hybrids exerted cytotoxicity in the 12–21 μM concentration range against the cancer cells; although, no significant differences can be observed between their activity. The ligands are not selective towards cancer cells over the normal cells. However, they were found to be somewhat more cytotoxic against the MDR cells than the sensitive ones. The tested RuCym complexes did not display measurable activity (IC_50_ > 100 μM), as it was also found for the analogous complex of HQCl-L-Pro [25]. On the contrary, the RhCp* complexes showed similar but somewhat lower cytotoxicity (IC_50_ = 19–32 μM) in comparison with the ligands with improved selectivity to the chemosensitive cancer cells over the MRC-5 cells (Figure 2). However, these RhCp* complexes did not display MDR selective toxicity as similar IC_50_ values were obtained in both cancer cell lines. This finding also suggests that these complexes are not the substrate of the efflux pump. Among the two RhCp* compounds, the HQCl-D-hPro complex is more cytotoxic; thus, the presence of the additional methylene moiety in the ligand seems to be beneficial in terms of biological activity.

**Figure 3:**
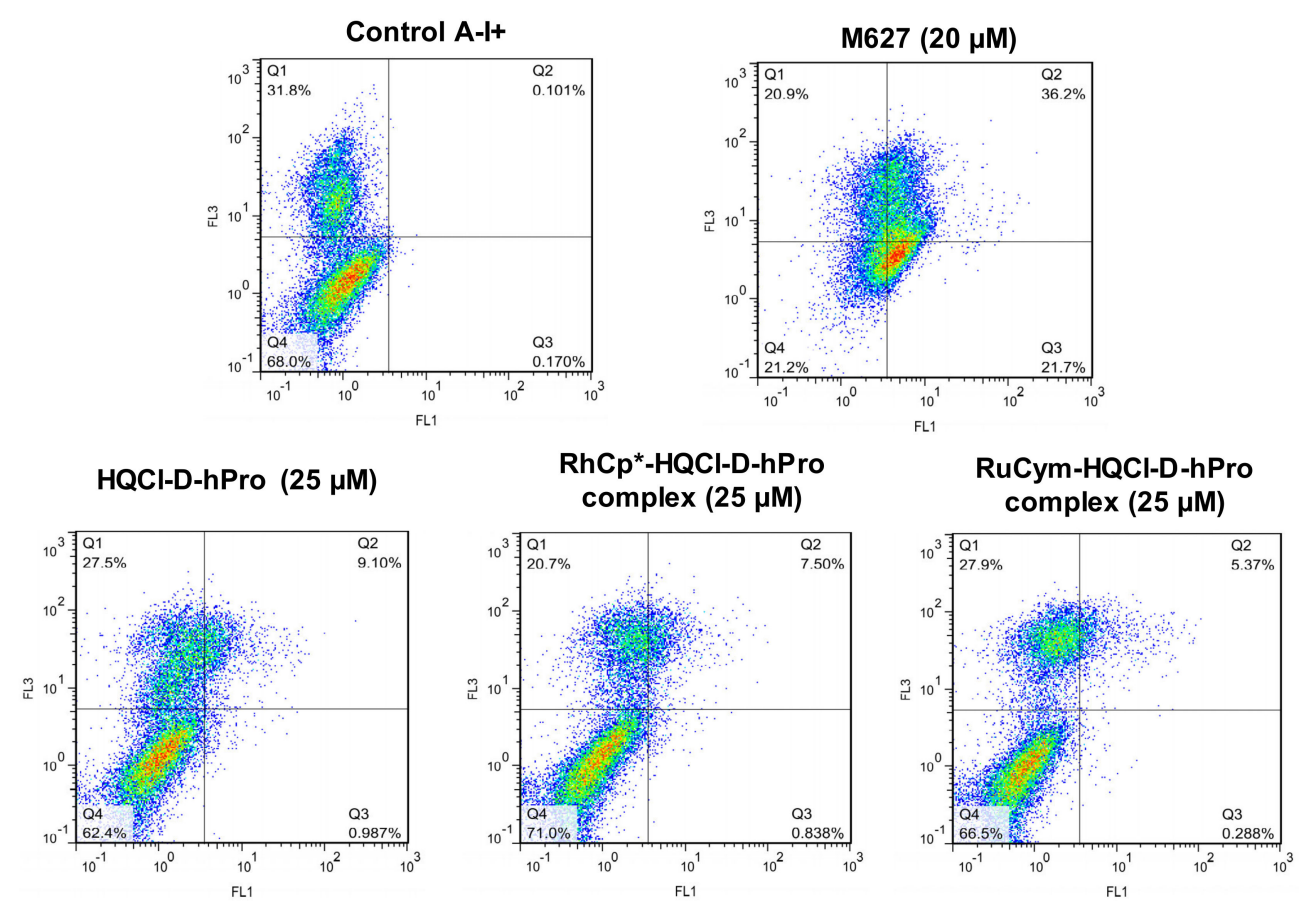
Apoptosis induction by metal complexes on MDR Colo-205 adenocarcinoma cells at 25 µM. M627 at 20 µM was applied as a positive control. Cell populations based on the flow cytometric analysis: **Q1**: dead cells (annexin negative, propidium iodide positive) (A−, PI+); **Q2**: late apoptotic cells (annexin positive, propidium iodide positive) (A+, PI+); **Q3**: early apoptotic cells (annexin positive, propidium iodide negative) (A+, PI−); **Q4**: living cells (annexin negative, propidium iodide negative) (A−, PI−); FL1: fluorescence of annexin V-FITC; FL3: fluorescence of propidium iodide.

The mechanism of cytotoxicity induced by HQCl-D-hPro and its RhCp* and RuCym complexes was assessed by the analysis of Colo-205 cells stained with fluorescein isothiocyanate-labeled Annexin-V and propidium iodide (PI) using flow cytometry. The applied concentration was selected based on the IC_50_ values of the cytotoxic derivatives (25 μM), and 3 h incubation time was applied. 12*H*-Benzo[α]phenothiazine (M627) and cisplatin were used as positive controls. Annexin-V assay offers the possibility of detecting early phases of apoptosis. Apoptosis is a morphologically and biochemically distinct form of programmed cell death, which is the preferred mode of action for an anticancer compound. PI was used to distinguish between viable, early apoptotic and necrotic or late apoptotic cells. The fluorescence of PI (FL3) is plotted versus Annexin-V fluorescence (FL1), as shown in Figure 3 for the HQCl-D-hPro and its RhCp* and RuCym complexes. Population (as a percentage of the various gated events) of early and late apoptotic and necrotic cells is provided in Table S4. HQCl-D-hPro and its complexes were not able to induce early apoptosis. A higher percentage of the late apoptotic and necrotic cells was obtained for the more cytotoxic HQCl-D-hPro (9.1%) and its RhCp* complex (7.5%) in comparison to the less toxic RuCym complex (5.4%). In order to better understand the different behavior of the studied compounds, an in-depth analysis of the various solution (physico-)chemical properties of the ligands and complexes were performed.

### 2.4. Solution Chemical Behavior of the Title Ligands and Their Organometallic Complexes

#### 2.4.1. Solution Equilibria and Lipophilicity of HQCl-D-Pro and HQCl-D-hPro

**Figure 4:**
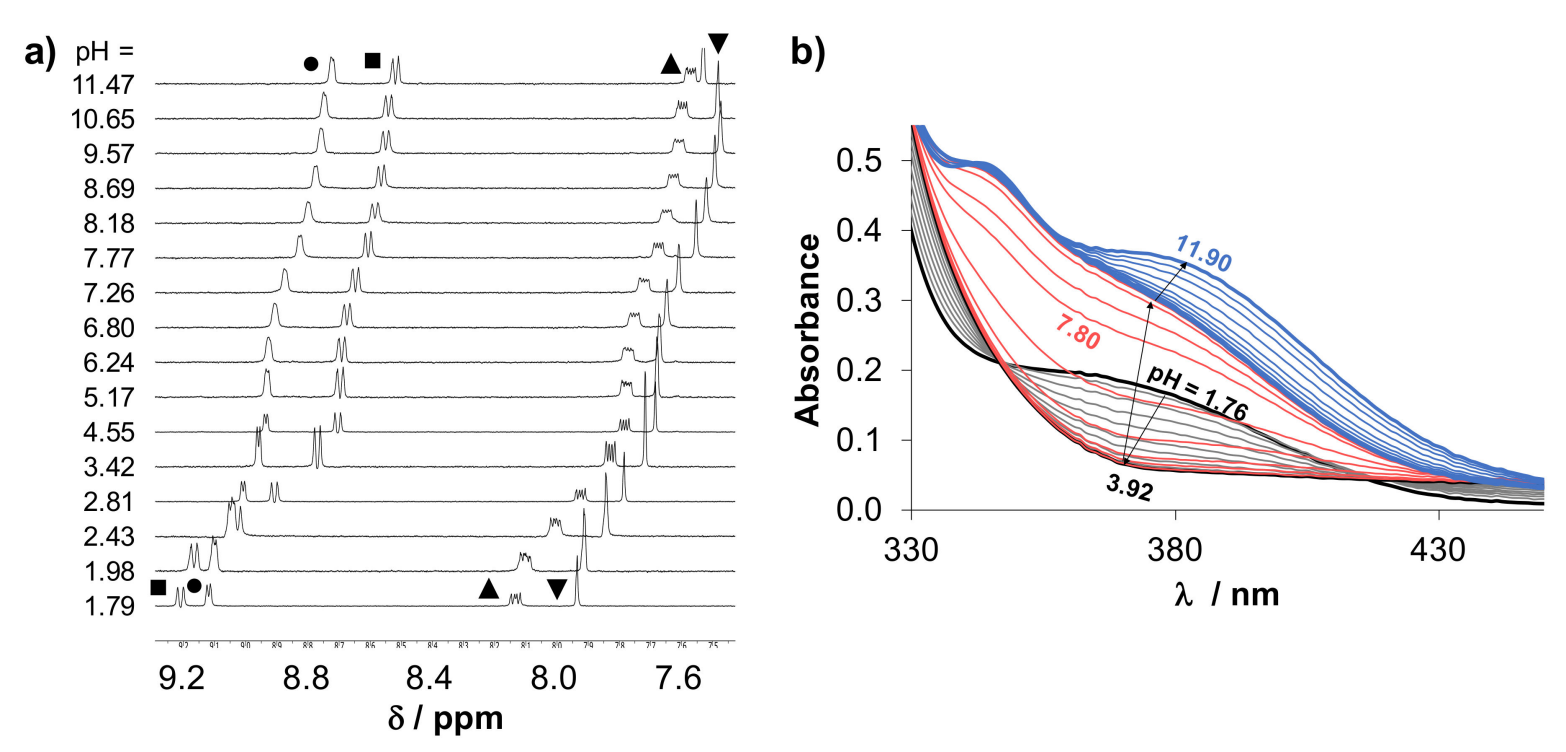
(**a**) ^1^H NMR spectra of HQCl-D-hPro recorded at various pH values in the low-field region. Peak assignation: C^2^H(■), C^4^H(●), C^3^H(▲) and C^6^H(▼). {*c* = 500 µM; 10% (*v*/*v*) D_2_O} (**b**) UV-vis spectra of HQCl-D-hPro at various pH values. {*c* = 117 µM; *I* = 0.2 M KNO_3_; *T* = 25.0 °C; *ℓ* = 1 cm}.

The pharmacokinetic behavior of a drug molecule is strongly affected by its physico-chemical properties, and the actual protonation state, the overall charge and lipophilicity are considered as key parameters. Therefore, proton dissociation processes of HQCl-D-Pro and HQCl-D-hPro were investigated in aqueous solution by pH-potentiometric, UV-visible (UV-vis) and ^1^H NMR spectroscopic titrations. Representative UV-vis and ^1^H NMR spectra recorded for HQCl-D-hPro are shown in Figure 4, and the p*K*_a_ values determined by the different techniques are collected in Table 2.

**Scheme 4:**
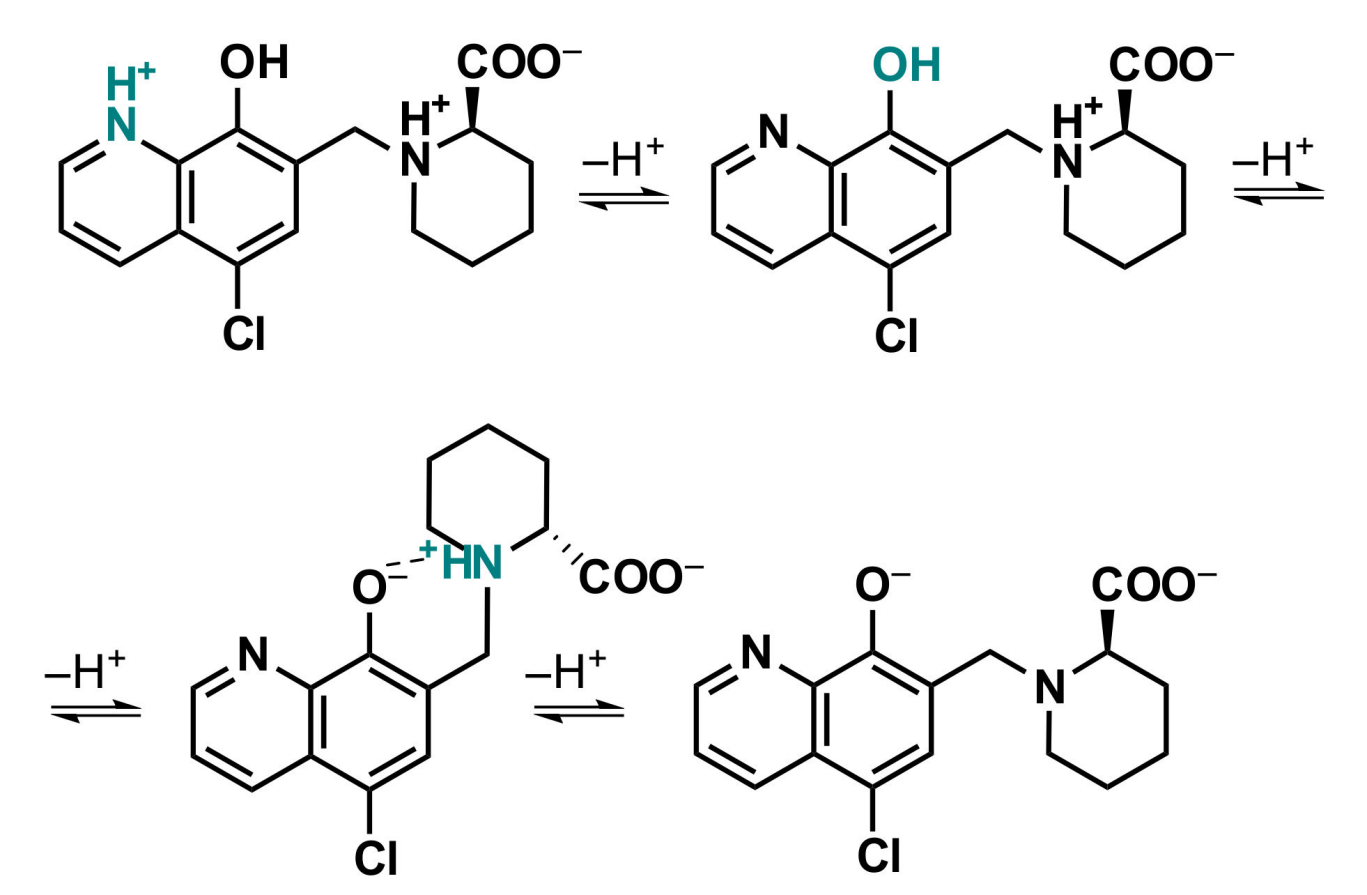
Deprotonation steps of HQCl-D-hPro in the monitored pH range. (Dashed line between O^−^ and NH^+^ indicates the hydrogen bond.)

These amino acid-8-hydroxyquinoline hybrids have theoretically four dissociable protons, although they could be characterized by only three proton dissociation processes in the monitored pH range (1.8–11.9), similar to that reported for HQCl-L-Pro [25]. The UV-vis and ^1^H NMR spectral changes (Figure 4) are also similar, and it is suggested that the order of the deprotonation steps is the following: quinolinium-NH^+^ (N_q_H^+^), phenolic hydroxyl group (OH) and the (homo)proline-NH^+^ (N_hPro/Pro_H^+^) moiety (Scheme 4) on the basis of the analysis of the spectra recorded at various pH values.

The proton dissociation of the carboxylic acid group takes place in the very acidic pH range (pH << 2), thus p*K*_a_ could not be calculated for this process. Based on the determined p*K*_a_ values (Table 2), it can be concluded that deprotonation of the N_q_H^+^ moiety takes place at the acidic pH range, while the (homo)proline-NH^+^ loses its proton at fairly high pH values. Consequently, these ligands possess zwitterionic amino acid moiety in a wide pH range. Notably, a hydrogen bond is most likely formed between the deprotonated phenolate group and the positively charged (homo)proline-nitrogen (Scheme 4), resulting in the relatively high p*K*_a_ (N_hPro/Pro_H^+^). The hydroxyl group of the HQ scaffold is characterized by a p*K*_a_ value, which is very close to the physiological pH and is somewhat lower for HQCl-D-hPro than for HQCl-D-Pro. Therefore, a lower fraction of the negatively charged species (O^−^, COO^−^, N_hPro/Pro_H^+^) is seen in the case of the latter ligand at pH 7.4 (HQCl-D-Pro: 30%; HQCl-D-hPro: 42%); although, the main fraction is still the neutral form (OH, COO^−^, N_hPro/Pro_H^+^) in both cases.

It is worth noting that these amino acid hybrids have excellent water solubility due to their zwitterionic form, unlike most 8-hydroxyquinolines. Distribution coefficients (*D*_7.4_) were determined at physiological pH, and log*D*_7.4_ = −0.02 ± 0.01 and +0.36 ± 0.02 (in 100 mM KCl) were obtained for HQCl-D-Pro and HQCl-D-hPro, respectively. These data represent the increased lipophilicity of the homoproline derivative due to the presence of the additional methylene group. Notably, the higher lipophilicity of the ligand HQCl-D-hPro did not result in a significant change in the cytotoxicity in comparison to HQCl-D-Pro, only in the case of the RhCp* complexes.

#### 2.4.2. Solution Equilibria and Lipophilicity of the Title Organometallic Complexes

To better understand the different behavior of the RhCp* and RuCym complexes of HQCl-D-Pro and HQCl-D-hPro against cancer cells, their solution chemical properties were characterized and compared. Our main aim is to determine equilibrium constants for the formation of these complexes, for the deprotonation of the coordinated aqua ligand and the non-coordinating (homo)proline amino group and for the possible water–chloride exchange process in chloride ion containing media (Scheme S1).

**Figure 5:**
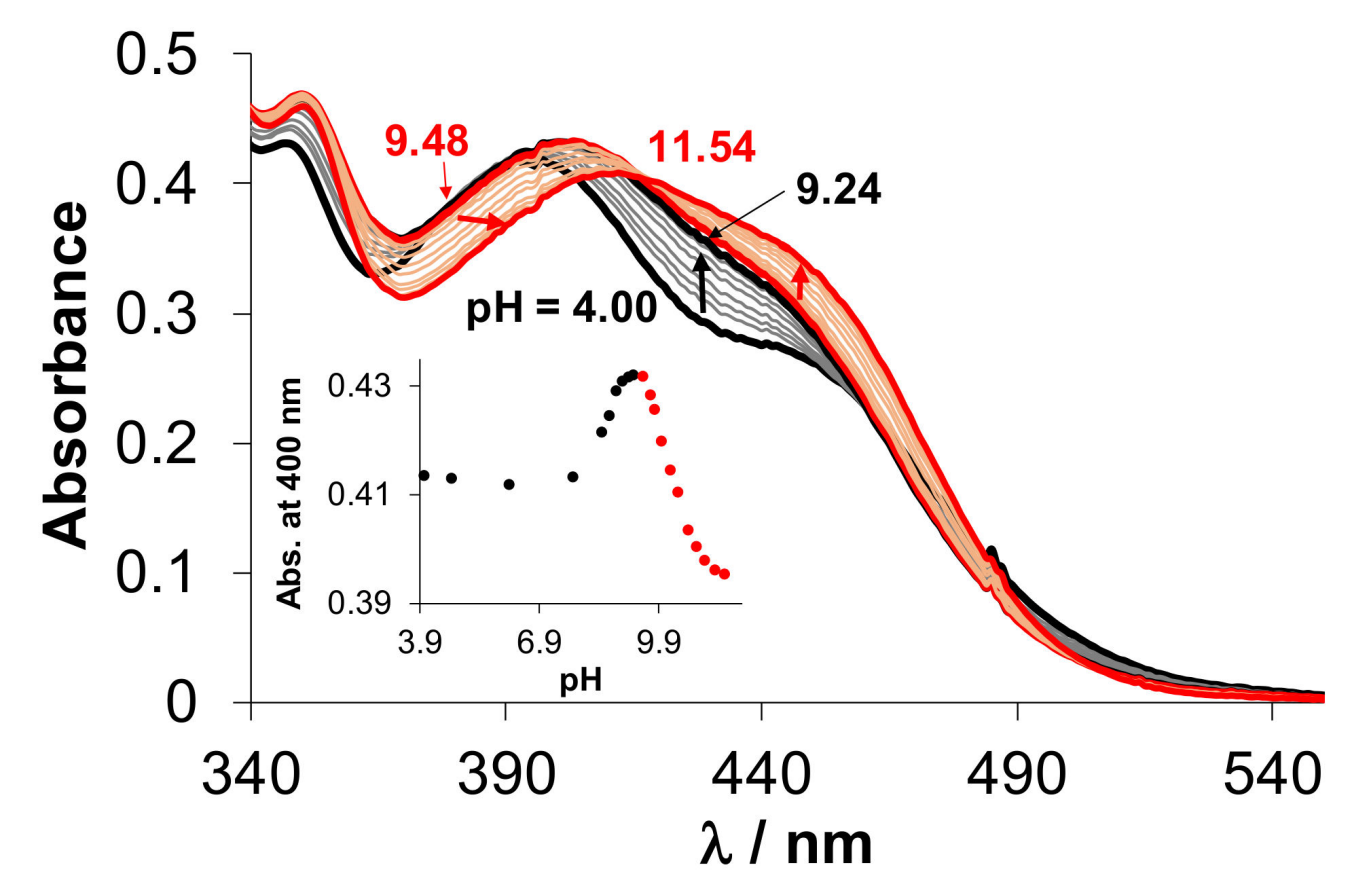
UV-vis spectra of the RuCym–HQCl-D-hPro complex at various pH values. Inserted figure shows the absorbance changes at 400 nm plotted against the pH. (Abs. stands for absorbance and arrows indicate the direction of the changes of the spectra with increasing the pH in the different pH ranges (black: between 4.00–9.24, red: 9.48–11.54.) {*c*_RuCym_ = *c*_ligand_ = 116 µM; *I* = 0.2 M KNO_3_; *T* = 25.0 °C; *ℓ* = 1 cm}.

As a first step, the solution stability of the complexes was checked in phosphate buffer at pH 7.4 using ^1^H NMR spectroscopy. The compounds were found to be stable during the monitored 72 h period. The ^1^H NMR and UV-vis spectra of the organometallic complexes were measured at 500 μM and 100 μM concentrations, respectively, by varying the pH, and the spectra were found to be unchanged between pH 1 and ~8 in the case of RuCym (Figure 5) and 1–~9 for RhCp* (not shown) complexes. By lowering the pH to 0.7, only ~3% of RhCp*–HQCl-D-hPro and ~2% of RuCym–HQCl-D-hPro complexes were dissociated after 24 h equilibration time according to the ^1^H NMR peak integral values (Figure S17). This low fraction of unbound organometallic ion and ligand did not allow the direct determination of stability constants of the complexes. (Notably, pH 0.7 is the most acidic pH that can be achieved, keeping the ionic strength constant at 0.2 M.) 

The complex formation processes were followed in the absence of chloride ions, similar to our previous studies [5,24,25,32], to obtain comparative data. Time dependence of UV-vis spectra recorded for the equimolar solution of the ligand and [M(arene)(H_2_O)_3_]^2+^ at pH 0.7 and 4.1 was followed. The charge-transfer band in the 340–500 nm region is a good indicator for complexation. Spectra showed significant complex formation immediately after mixing the reactants at pH 0.7, and the equilibrium could be reached relatively fast for the RhCp* complexes (HQCl-D-hPro: ~3 min, HQCl-D-Pro: ~30 min), while the formation of the RuCym complexes was significantly slower (~7 h). As the actual protonation state of the ligand changes by increasing pH, it was also found that at higher pH the complexation becomes faster. The equilibration time decreased to <2 min for both RhCp* complexes at pH 4.1, while ~1.5 h was needed for the RuCym complexes, as it is shown in Figure S18. Although, in the isolated solid complexes, the neutral ligands are coordinated, in solution under this condition, the formation of complexes with the general formula [M(arene)(HQCl-D-(h)ProH_−1_)(H_2_O)]^+^ is expected due to the deprotonation of the non-coordinating carboxyl group. In summary, these findings clearly indicate the high stability of the title complexes and the considerable difference in the rate of complex formation with the two types of metal ions. 

**Figure 6:**
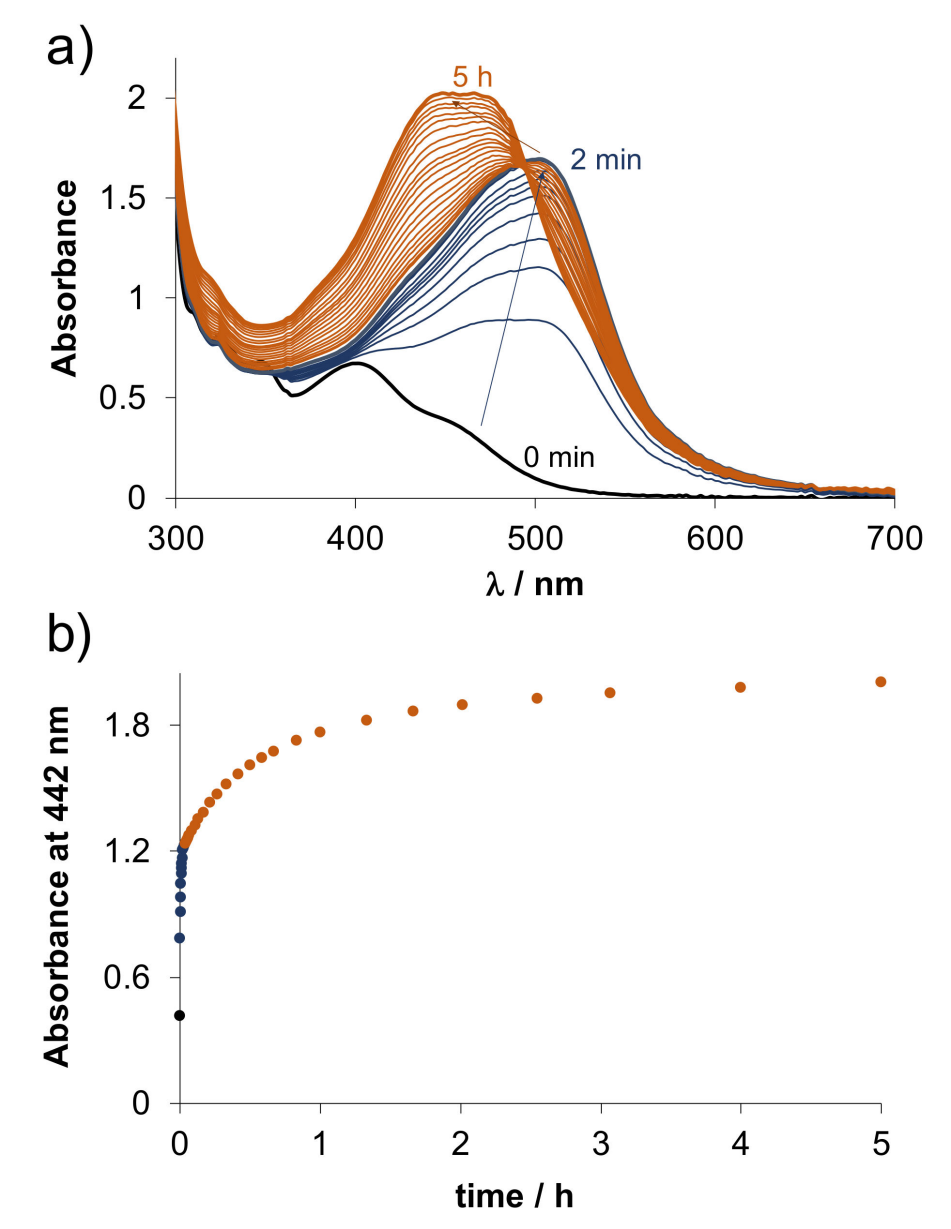
(**a**) Time-dependent UV-vis spectra of the RuCym–HQCl-D-Pro complex in the presence of 2 equiv. phen at pH = 7.40, and (**b**) absorbance changes at 442 nm. (Dark blue color indicates the first process: liberation of *p*-cymene and coordination of the first phen; orange color indicates the second process: coordination of the second phen.) {*c*_RuCym_ = *c*_HQCl-D-Pro_ = 200 µM; *c*_1,10-phenantroline_ = 400 µM; pH = 7.40 (20 mM phosphate buffer); *T* = 25.0 °C; *ℓ* = 1 cm}.

Formation constants for the RhCp* complexes of HQCl-D-hPro and HQCl-D-Pro were determined via ligand displacement studies using the (N,N) donor set bearing 2-picolylamine. This competitor ligand was gradually added to the RhCp*–8-hydroxyquinoline complexes at pH 7.4, and UV-vis and ^1^H NMR spectra were recorded (see Figure S19 for HQCl-D-hPro complex). ^1^H NMR measurements confirmed that the suggested displacement reaction takes place (Figure S19b). Based on the significant UV-vis spectral changes, stability constants log*K* = 13.35 ± 0.03 and 13.48 ± 0.03 were calculated for the complexes of HQCl-D-hPro and HQCl-D-Pro, respectively, using the known stability constant of the RhCp*–2-picolylamine complex [32]. These values are similar to that of the complex of HQCl-L-Pro (log*K* = 13.41 [25]), suggesting similar stability of the proline and homoproline bearing complexes. Unfortunately, this approach could not be applied for the RuCym complexes since irreversible arene loss and the slow oxidation of Ru(II) were observed due to the addition of 2-picolylamine, similar to that found for the RuCym complex of HQCl-L-Pro [25]. Besides 2-picolylamine, the rigid aromatic 1,10-phenantroline (phen, Figure 6) or the excess of the 8-hydroxyquinoline ligand itself (Figure S20) also results in the loss of *p*-cymene. Two main processes were seen upon the addition of two equiv. phen to the complex (Figure 6). The first step is most probably the loss of the arene ring and the formation of a mixed-ligand complex [Ru(II)(HQCl-D-ProH_−1_)(phen)(H_2_O)_2_]^+^ (λ_max_ = 500 nm), which is followed by the slower binding of the second phen. Notably, there is no indication for the oxidation of the metal center in this case, as the coordination of two phen ligands can stabilize the +2 oxidation state; unlike in the case of HQCl-D-Pro excess, where the slow development of bands in the wavelength range 550–700 nm suggests the oxidation (Figure S20). 

In the basic pH range, both the UV-vis and ^1^H NMR spectra recorded for the [M(arene)(H_2_O)_3_]^2+^–ligand (1:1) systems reveal the further chemical transformation of the [M(arene)(HQCl-D-(h)ProH_−1_)(H_2_O)]^+^ species. Based on the UV-vis spectra measured for the RuCym–ligand systems (see Figure 5 for HQCl-D-hPro), two processes take place, which are overlapping, and they were identified as the deprotonation of the coordinated water molecule and the non-coordinating (homo)proline nitrogen (Scheme S1). The ^1^H NMR spectra show that the chemical shifts of the proline-CH_2_ (and CH) moieties are mainly sensitive to the proline nitrogen deprotonation (Figure S21), and they change at somewhat higher pH values than, e.g., those of the *p*-cymene CH_3_ and 8-hydroxyquinoline CH protons (Figure S21). It should be noted that peaks of the *p*-cymene CH_3_ groups are doubled at pH > 10 (Figure S21b), indicating the loss of their symmetry due to the formation of the hydroxido complex, which probably blocks the free rotation of the arene ligand. Two p*K*_a_ values were determined by both methods for each RuCym complex that is in good agreement (Table 3).

These deprotonation processes take place at somewhat higher pH in the case of the RhCp* complexes compared to the RuCym compounds; however, other differences were also observed. UV-vis spectra measured for RhCp*–HQCl-D-hPro (1:1) system at various pH values indicate only a single process as isosbestic points are seen (Figure S22a), as most probably in this case, the charge transfer bands are not sensitive enough for the deprotonation of the non-chromophoric homoproline-N.

**Figure 7:**
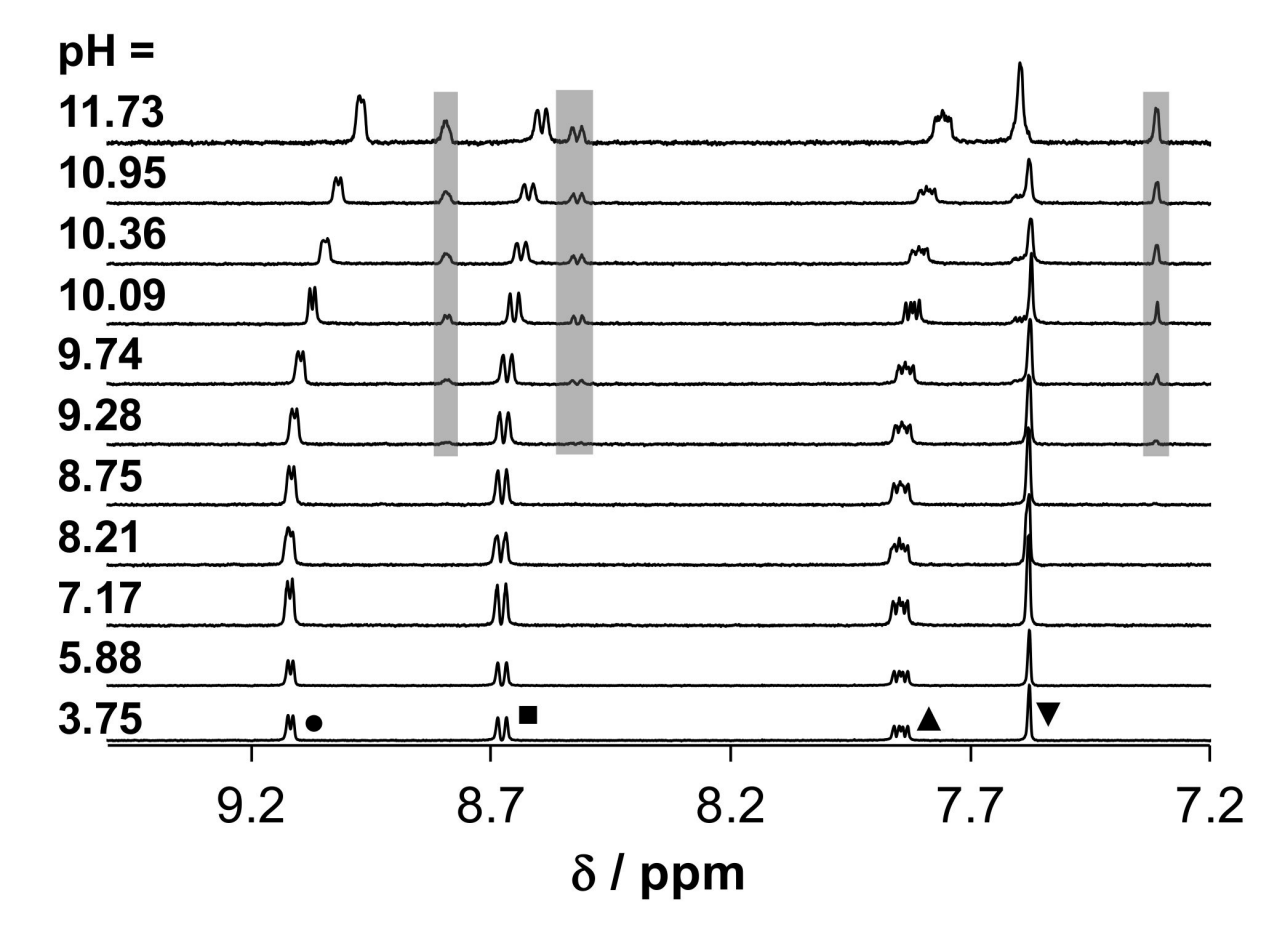
^1^H NMR spectra of the RhCp*–HQCl-D-Pro (1:1) system at various pH values in the high-field region. For the peak assignation, see the legend of Figure 4. Grey boxes show the peaks of the minor species. {*c*_RhCp*_ = *c*_ligand_ = 500 μM; 10% (*v*/*v*) D_2_O); *I* = 0.2 M KNO_3_; *T* = 25.0 °C}.

Thus, only one p*K*_a_ value could be computed (Table 3), which is rather assigned to the H_2_O → OH^−^ process. The peaks of the protons in the ^1^H NMR spectra located nearby the metal center (both 8-hydroxyquinoline CH and CH_3_ protons of RhCp*) and the peaks of the homoproline moiety are shifted by increasing the pH (Figure S22b,c). This suggests that both deprotonation processes might be realized. When the two sets of chemical shifts (Figure S22c) are evaluated separately, p*K*_a_ = 10.06 ± 0.06 and p*K*_a_ = 9.82 ± 0.13 can be obtained from the signals of the C^4^H proton of 8-hydroxyquinoline moiety, and C^15^H proton of hPro, respectively. This finding suggests that the mixed hydroxido complex appears at a more basic pH compared to the analogous RuCym complex. By the deconvolution of the UV-vis spectra obtained for the RhCp*–HQCl-D-Pro (1:1) system, only one p*K*_a_ could be computed (Table 3), assigned to the aqua ligand deprotonation. However, novel peaks are observed in the ^1^H NMR spectra at pH > 8.7 (grey boxes in Figure 7), which are pH-independent and do not belong to an unbound ligand (or organometallic ion). Due to this side-reaction, no p*K*_a_ values were calculated from the ^1^H NMR spectra.

Samples were prepared at pH 11.0 in which the concentration of the RhCp*–HQCl-D-Pro complex was varied in a wide range (2.33 mM–50 µM), and ^1^H NMR and UV-vis spectra (Figure S23) were measured. The normalization of the UV-vis spectra showed only minor changes in the charge transfer band (330–530 nm), and the fraction of the minor species estimated on the basis of peak integrals from the ^1^H NMR spectra was ca. 22–28% in the monitored concentration range. When *N*-methylimidazole was added in increasing amount to the complex due to its monodentate binding at the coordination site of the co-ligand, the fraction of the minor species was gradually decreased (Figure S24). This minor species might be formed by the coordination of the Pro carboxylate moiety resulting in the tridentate binding of the ligand, or the proline nitrogen of a second ligand might coordinate, and a dimeric species is formed. The latter suggestion could not be confirmed by ESI-MS measurements, and the relatively constant fraction of the minor species in the applied wide concentration range is also against it. Interestingly, the formation of additional species was not observed with the 6-membered ring HQCl-D-hPro possibly due to steric reasons. 

**Figure 8:**
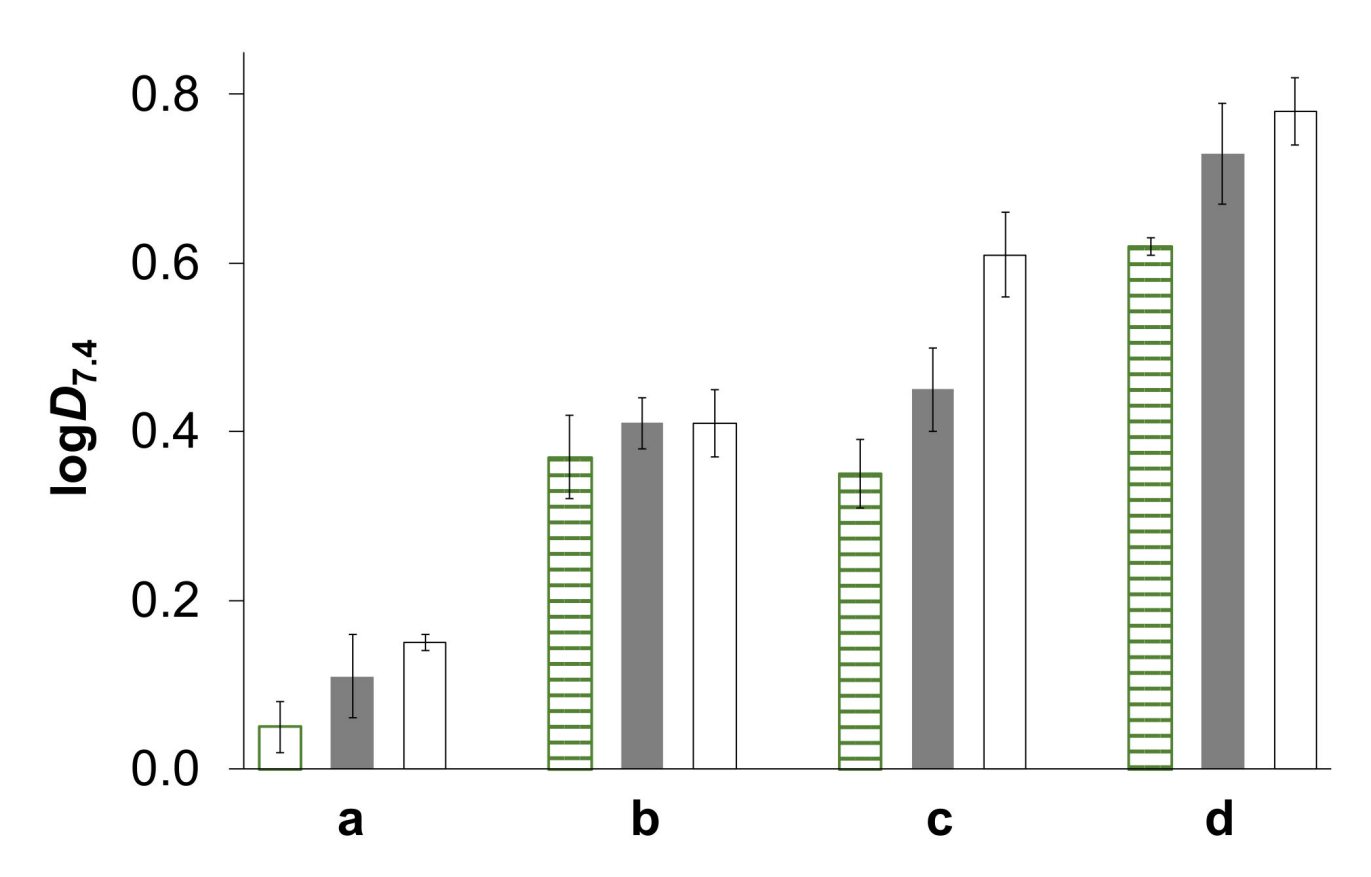
Lipophilicity of the title complexes expressed as log*D*_7.4_ measured via *n*-octanol/water partitioning at pH = 7.40 at three types of chloride ion concentrations: 4 mM (stripped bar), 24 mM (grey bar) and 100 mM (white bar). a: RhCp*–HQCl-D-Pro, b: RhCp*–HQCl-D-hPro, c: RuCym–HQCl-D-Pro, d: RuCym–HQCl-D-hPro. {*c*_complex_ = 150 µM; *T* = 25.0 °C}.

Based on the determined p*K_a_* values of the complexes (Table 3), it can be concluded that at physiological pH all of them are present predominantly in their [M(arene)(HQCl-D-(h)ProH_−1_)(H_2_O)]^+^ form. However, in the presence of chloride ions, the exchange of the aqua ligand to the halide ligand is possible. The water/chloride co-ligand exchange process was monitored spectrophotometrically at pH 7.4, as it was performed for other RuCym and RhCp* complexes in our previous works [5,6,9,24,25,32]. Log*K*′ (H_2_O/Cl^−^) values computed by the deconvolution of the UV-vis spectra (exemplary spectra are shown for RuCym–HQCl-D-hPro complex in Figure S25) are collected in Table 3. It can be concluded that the RhCp* complexes have stronger chloride ion affinity. The coordination of the chloride ion changes the charge of the complex; thus, it will also influence lipophilicity. In order to characterize the hydrophilic/lipophilic character of the complexes, *n*-octanol/buffer aqueous solution partitioning was performed at pH 7.4. The measurements were performed at three kinds of chloride ion concentrations regarding different biofluids (4, 24 and 100 mM for nucleus, cytosol and blood, respectively). The calculated log*D*_7.4_ values can be found in Figure 8.

By increasing the chloride ion concentration, the lipophilicity is increased due to the formation of a more neutral [M(arene)(HQCl-D-(h)ProH_−1_)(Cl)] complex. The homoproline hybrids are more lipophilic, as is expected based on the different lipophilic properties of the ligands. The higher cytotoxic activity of the RhCp*–HQCl-D-hPro complex over the HQCl-D-Pro complex might be related to its increased lipophilicity. It should be noted that the RuCym complexes are more lipophilic than the analogous RhCp* complexes, although they were less cytotoxic, indicating that besides lipophilicity, other factors obviously can influence the pharmacological activity. 

### 2.5. Binding of the Complexes to Biological Macromolecules

#### 2.5.1. Interaction of the Complexes with Human Serum Albumin

HSA is the most abundant protein in the circulatory system and has an important role in the transport of drug molecules, strongly affecting their pharmacokinetic properties [33]. On the other hand, it can also serve as a general model to investigate the interaction between an organometallic complex and a protein. As a first step, the binding of the RuCym and RhCp* complexes of HQCl-D-Pro and HQCl-D-hPro to HSA was followed by UV-vis spectrophotometry in time at pH 7.4. The spectral changes (not shown) revealed relatively slow binding processes, and the equilibrium could be reached after 3–4 h for the HQCl-D-Pro complexes, similar to that found for the HQCl-L-Pro bearing compounds [34], while 10 h was needed for the HQCl-D-hPro complexes irrespectively of the type of the metal ion. Therefore, we chose the equilibration time as 24 h for further measurements.

**Figure 9:**
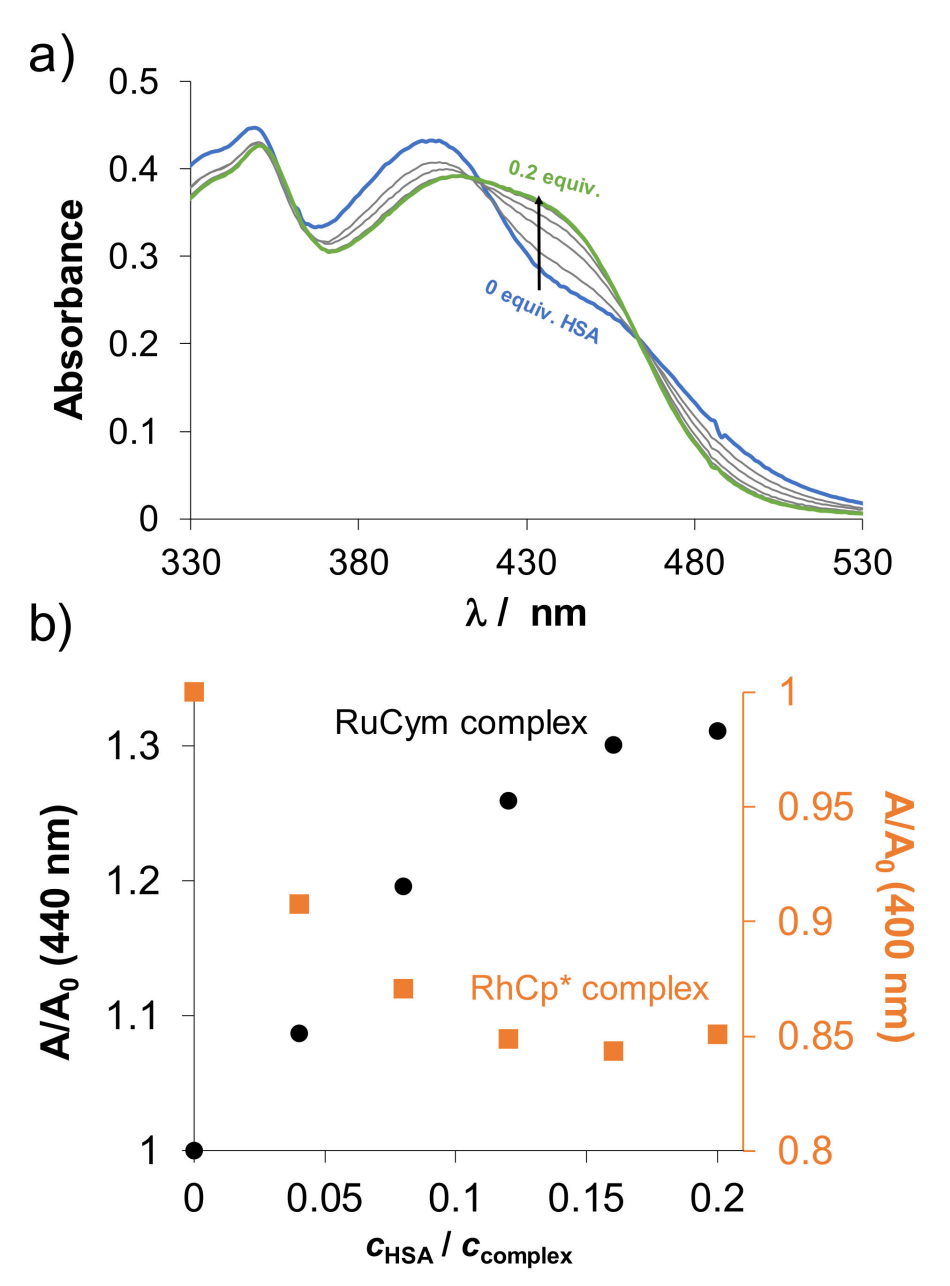
(**a**) UV-vis spectra of RuCym–HQCl-D-hPro complex in the presence of various equiv. HSA. (Arrows show the spectral changes with increasing equiv. HSA) (**b**) Relative absorbance changes against *c*_HSA_/*c*_complex_ ratio of RuCym–HQCl-D-hPro complex (●) in comparison with RhCp*–HQCl-D-hPro (■). (A is the measured absorbance at 440 nm at the given ratio, A_0_ is the initial absorbance of the complex without HSA.) {*c*_complex_ = 100 µM; *c*_HSA_ = 0–20 µM; *T* = 25.0 °C; *ℓ* = 1 cm; pH = 7.40 (PBS’)}.

UV-vis spectra were also recorded at various complex-to-HSA ratios (for HQCl-D-hPro complexes, see Figure 9), and upon the addition of the protein, the absorbance spectra changed gradually. The changes ended in the presence of ca. 0.16 (RhCp*) and 0.2 (RuCym) equiv. HSA, which indicates a high number (≥5) of possibly bound complexes. Based on the ^1^H NMR spectra of the complexes recorded in the presence of 0.5 equiv. HSA (see Figure S26 for RhCp*–HQCl-D-Pro complex), it was concluded that peaks belonging to unbound complex or ligand could not be detected, and the appearance of multiple peaks suggests different binding environments around the organometallic center.

Additionally, the global binding profile of the complexes was studied by capillary zone electrophoresis (CZE), as the free complex and the protein-bound species have different electrophoretic mobility, thus, different migration times. Analysis of the electropherograms (see Figure S27 for the RuCym–HQCl-D-Pro complex) reveals the decreasing fraction of the free complex with increasing HSA/complex ratio (Figure S28), and practically all the complexes are already bound at a low equiv. HSA (≤0.2), as it was suggested on the basis of the UV-vis measurements as well. Peaks of the free ligand were not detected in the electropherograms; thus, the binding of the complex is not accompanied by ligand displacement. Based on the binding curves calculated from the CZE results (Figure S28), the HQCl-D-hPro complexes bind to the protein with a higher affinity in comparison to the D/L-Pro derivatives and a somewhat weaker binding of the complexes containing the Pro amino acid with D-configuration is also observed. 

The slow binding kinetics indicates coordination binding mode, and the monodentate coordination of a side chain donor of HSA takes place most likely by the replacement of the co-ligand water or chloride, while the original bidentate (N,O) ligand remains coordinated, as it was also reported to analogous high stability RuCym and RhCp* complexes [34,35]. Among others, surface-exposed His side chain residues are suggested as main binding sites for half-sandwich RuCym and RhCp* complexes [34,35,36,37,38]. Short N- and C-terminally protected tripeptides such as Ac-Ala–His–Ala-NH_2_ (AHA), Ac-Phe–His–Ala-NH_2_ (FHA) oligopeptides were used as His binding models for the RuCym and RhCp* complexes of HQCl-L-Pro in our previous work [34].

The HQCl-L-Pro complexes displayed strong binding to these oligopeptides (RhCp* complex: log*K*′ = 4.2 (AHA), 4.7 (FHA), RuCym complex: log*K*′ = 4.4 (AHA), 5.1 (FHA) [34], and no correlation with either lipophilicity or chloride ion affinity was found. The RuCym–HQCl-L-Pro complex reacted much slower (3 h) with the tripeptides in comparison to the RhCp* complex (few seconds) [34].

Similar reaction rates with FHA were found for the HQCl-D-Pro and HQCl-D-hPro complexes, and the reaction with this simple oligopeptide is much faster compared to the HSA. Conditional stability constants determined for the complex-FHA ternary species by ^1^H NMR spectroscopic measurements (exemplary spectra are presented in Figure S29) are shown in Table 4. The differences between the constants are not significant (except for the complex RuCym–HQCl-L-Pro); thus, the similar binding of these complexes to FHA is suggested. In all, this oligopeptide model can serve as a good binding model for surface-exposed side chains, although it provides limited information about binding events at more deeply located binding pockets of HSA, where the role of secondary interactions might be more significant.

**Figure 10:**
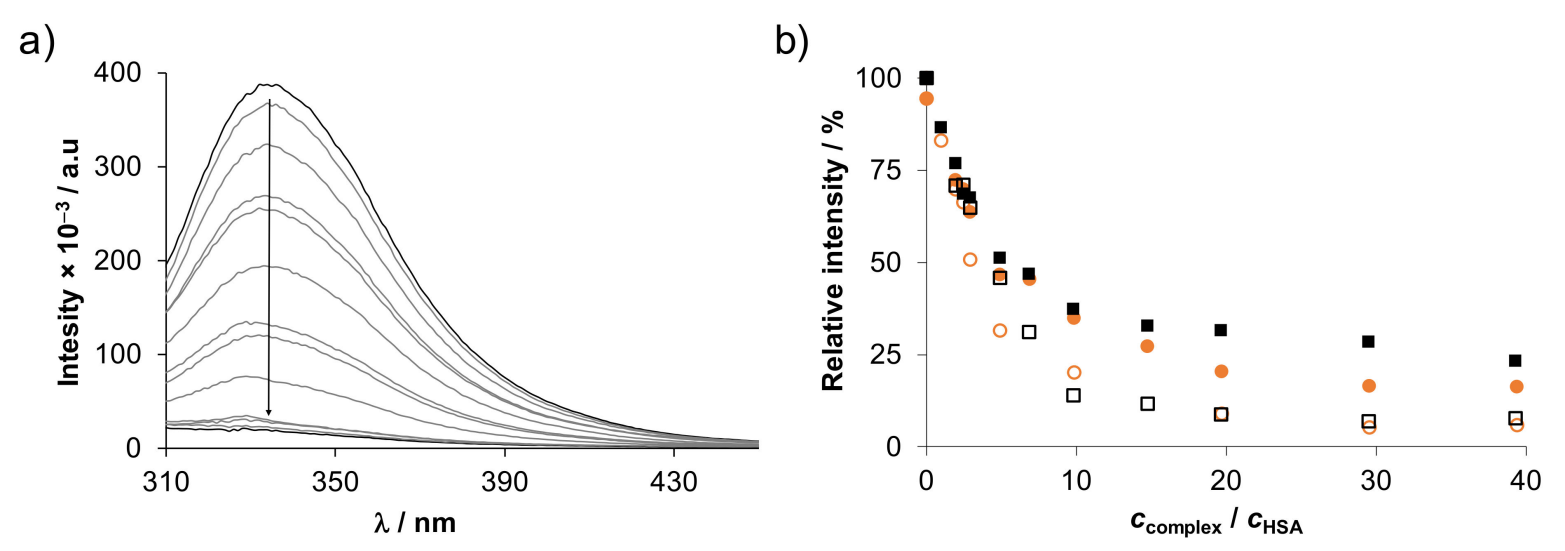
(**a**) Fluorescence emission spectra recorded for HSA upon addition of various amount of RhCp*–HQCl-D-hPro complex using λ_EX_ = 295 nm. (**b**) Intensity changes at 330 nm plotted against *c*_complex_/*c*_HSA_ ratio. Symbols for the complexes: RhCp*–HQCl-D-Pro (**□**), RhCp*–HQCl-D-hPro (○), RuCym–HQCl-D-Pro (■) and RuCym–HQCl-D-hPro (●). {*c*_HSA_ = 1 µM, *c*_complex_ = 1–40 µM; pH = 7.40 (PBS’ buffer)}.

Therefore, binding of the RuCym and RhCp* complexes of HQCl-D-Pro and HQCl-D-hPro into the two main hydrophobic binding pockets of HSA located in subdomain IIA (site I) and IIIA (site II) was monitored by spectrofluorometry. Tryptophan (Trp-214) quenching and dansylglycine (DG) site marker displacement experiments were performed to calculate binding constants for site I and II, respectively, similar to our previous works [9,34,38]. Representative emission spectra for the quenching experiment and relative intensity changes are shown in Figure 10.

The binding constants (Table 4) calculated on the basis of the fluorescence spectral changes indicate significant binding into both hydrophobic binding pockets. The differences are minor; however, it can be concluded that the binding of the complexes bearing the L-configuration proline is somewhat stronger, showing that chirality can also influence the interaction, while the Pro/hPro exchange resulted in similar or lower constants at site I and higher at site II. Comparing the inactive RuCym and the cytotoxic RhCp* complexes, it should be noted that the difference in the extent of their HSA binding is insignificant; thus, it cannot explain their different bioactivity. 

Possible changes in the secondary structure of HSA due to its interaction with the RhCp* and RuCym complexes of HQCl-D-hPro were investigated by CD spectroscopy. HSA is mostly composed of α-helices, and its CD spectrum can be characterized by two negative bands (208 and 222 nm). In the presence of the complexes, the α-helix was slightly affected by the addition of the complexes up to 5 equivalents (Figure S30); minor absorbance changes were seen at 208 and 222 nm, and the α-helix content was decreased from 50% to 47% or 46% in the case of the RhCp* and RuCym complex, respectively, due to our estimation based on the method suggested by Micsonai et al. [39]. Only at higher complex excess (>10) we observed somewhat larger changes; however, such protein-to-complex ratios are far from the biologically relevant conditions.

In summary, HSA is a potential binder of the title complexes and may act as their carrier leading to an increased accumulation in the tumor cells due to the enhanced permeability and retention effect.

#### 2.5.2. Interaction of the Complexes with DNA

**Figure 11:**
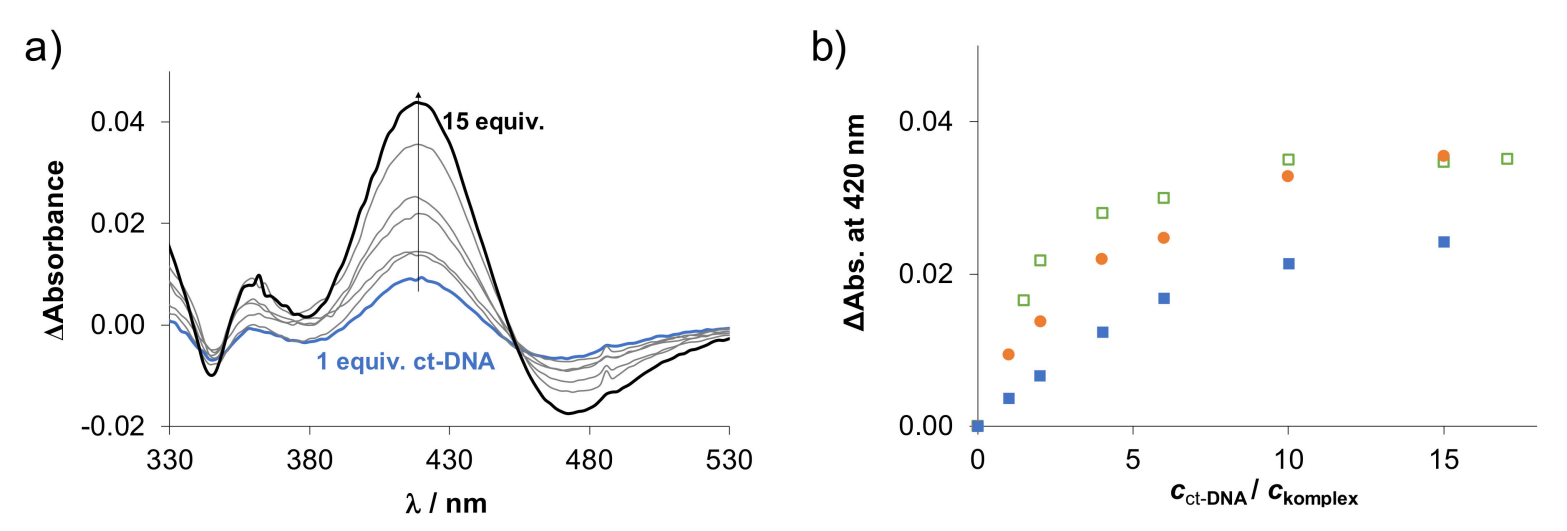
(**a**) UV-vis difference spectra of RhCp*–HQCl-D-hPro complex in the presence of various equiv. ct-DNA (difference spectrum = spectrum of the complex with ct-DNA–spectra of the complex and the ct-DNA alone). (Arrow shows the changes of the ΔAbsorbance values.) (**b**) Changes of ΔAbsorbance values (ΔAbs.) at 420 nm at increasing ct-DNA/complex ratio. Symbols for the ligands in the RhCp* complexes: HQCl-D-Pro (**□**), HQCl-L-Pro (■) and HQCl-D-hPro (●). {*c*_complex_ = 88–100 µM, *c*_ct-DNA_ = 0–1.75 mM, *c*_KCl_ = 4 mM; pH = 7.40 (20 mM phosphate buffer); *ℓ* = 1 cm}.

DNA is a classical target for metallodrugs in general; however, other primary targets such as proteins are also considered for RuCym and RhCp* complexes [40,41]. Herein, the interaction of the title complexes and their HQCl-L-Pro analogs towards calf thymus DNA (ct-DNA) was investigated at pH 7.4 and at 4 mM chloride concentration, which corresponds to the chloride content of the nucleus. As a first step, the reaction rate was monitored for the interaction between the complexes and ct-DNA using UV-vis spectrophotometry, as is shown for the RuCym-HQCl-D-hPro–DNA system in Figure S31. Depending on the metal center, the equilibrium could be reached in different time intervals; namely, for RhCp* complexes, it was 2–6 min, while slower interaction was typical for the RuCym complexes (2–3 h). UV-vis spectra were also recorded at various ct-DNA-to-complex ratios, and difference spectra were calculated as it is presented for the RhCp*–HQCl-D-hPro complex in Figure 11a (for the RuCym–HQCl-L-Pro complex, see Figure S32). Absorbance changes are shown for the RhCp* complexes of HQCl-L-Pro, HQCl-D-Pro and HQCl-D-hPro (Figure 11b). The changes in the absorbance reach the saturation value at lower ct-DNA/complex ratios in the case of the complexes with the D-forms of the amino acids compared to HQCl-L-Pro bearing species suggesting the weaker binding of the latter complex. The tested RuCym complexes behaved similarly; however, the spectral changes were even smaller, which did not allow direct comparison. Such changes of the charge transfer bands in the UV-vis spectra suggest coordination of ct-DNA donor atoms (most likely via a nitrogen donor monodentate) to the metal center. 

**Figure 12:**
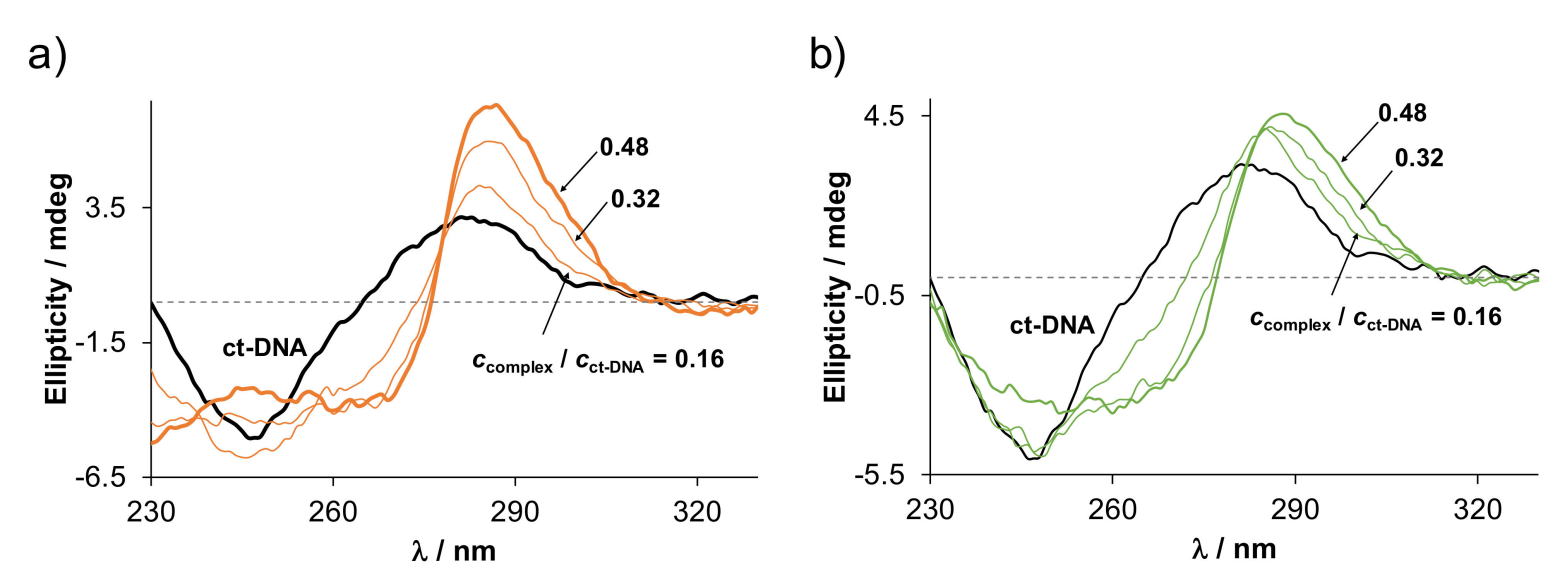
CD spectra of ct-DNA in the absence and presence of various equiv. (**a**) RhCp*-D-HQCl-hPro and (**b**) RuCym-D-HQCl-hPro. (The unit mdeg is millidegree.) {c_complex_ = 0–47 µM, c_ct-DNA_ = 98 µM, c_KCl_ = 4 mM; pH = 7.40 (20 mM phosphate buffer); ℓ = 1 cm} Notably, at higher than 50 µM complex concentration no reliable spectra could be recorded due to the high absorbance of the solution.

For further studies, the most cytotoxic RhCp*–HQCl-D-hPro complex and its inactive RuCym analog were selected to compare their DNA binding efficacy. ct-DNA has well-defined circular dichroism (CD) signal, including a negative band at around 247 nm and a positive one around 281 nm, due to the helical B conformation and nucleobase stacking, respectively [42]. Changes in the CD spectra of ct-DNA were recorded at different complex-to-ct-DNA ratios for both HQCl-D-hPro complexes (Figure 12). A significant bathochromic shift of the positive band (λ_max_ = 281 nm → 288 nm) could be observed upon the addition of the complexes, and the ellipticity was increased, especially for the RhCp* complex. The negative band was also shifted to the higher wavelengths but with decreasing ellipticity. It is reported that the bathochromic shifts are often the indicators of a coordinative bond forming between the metal ion and the nucleobase [43,44]. On the other hand, the increase in the ellipticity of the positive band can indicate changes in nucleobase stacking interactions, which is often related to an intercalative binding mode as well as the appearance of an induced negative CD signal at around 300 nm [43,44]; however, the latter phenomenon was not observed in our case. In all, the UV-vis and CD spectroscopic results rather suggest that coordination occurs between the metal center and nucleobase of ct-DNA in the case of both the RuCym and the RhCp* complexes and intercalation is not likely. 

Therefore, the interaction of RhCp* and RuCym complexes of HQCl-D-hPro with ct-DNA was further investigated via the widely used displacement assay with the intercalating ethidium bromide (EB) by steady-state and time-resolved spectrofluorometry to check the possibility of the intercalation. Upon addition of the complexes to EB–ct-DNA (1:4) samples, the fluorescence intensity was decreased (Figure S33), but surprisingly the signal fell below the intensity of the free EB. The change in the relative intensity shows a sigmoid character for both complexes, which suggests a more complex binding event than a simple EB displacement process.

Then fluorescence decay of the ct-DNA–EB system was followed at increasing concentrations of the RhCp*–HQCl-D-hPro complex (shown in Figure S34a). Lifetime data were obtained by the time-correlated single-photon counting (TCSPC) technique for both complexes and are collected in Table S5. It was found that the high excess of the metal complexes (10–15 equiv.) resulted in a very similar decay curve compared to that of the free EB. The system could be characterized by two lifetimes related to free EB and DNA-bound EB, which are in good agreement with the literature data [45]. The lifetimes were not altered significantly upon addition of the complex (τ_1_ = 1.69–1.57 ns (EB); τ_2_ = 21.78–19.99 ns (bound EB)), while the corresponding amplitude values (α_1_; α_2_) were changed considerably indicated in Figure S34b. Based on these data, it is estimated that intensity obtained in the absence of complex and at lower metal complex-to-ct-DNA ratios (4–6) mostly comes from the bound EB. On the other hand, at higher equivalents of the complex, it seems to come from the free EB. As the steady-state measurements showed the unusually low emission upon the EB displacement, the direct interaction between the complexes and EB was also studied by fluorometry (Figure S35). The addition of the RuCym–D-HQCl-hPro complex to EB decreased the intensity, and static quenching of EB took place according to lifetime experiments (Figure S35b). (The RhCp* complex behaved similarly.) Namely, a non-fluorescent product is formed, which could also be present in ternary EB–ct-DNA–complex systems. 

In all, we found that EB was released upon binding of the complexes to ct-DNA; however, intercalation of the complexes is not suggested; it is more probable that their binding via coordinative bonds to the macromolecule modifies the EB binding capacity of ct-DNA, leading to the liberation of this intercalating agent.

**Figure 13:**
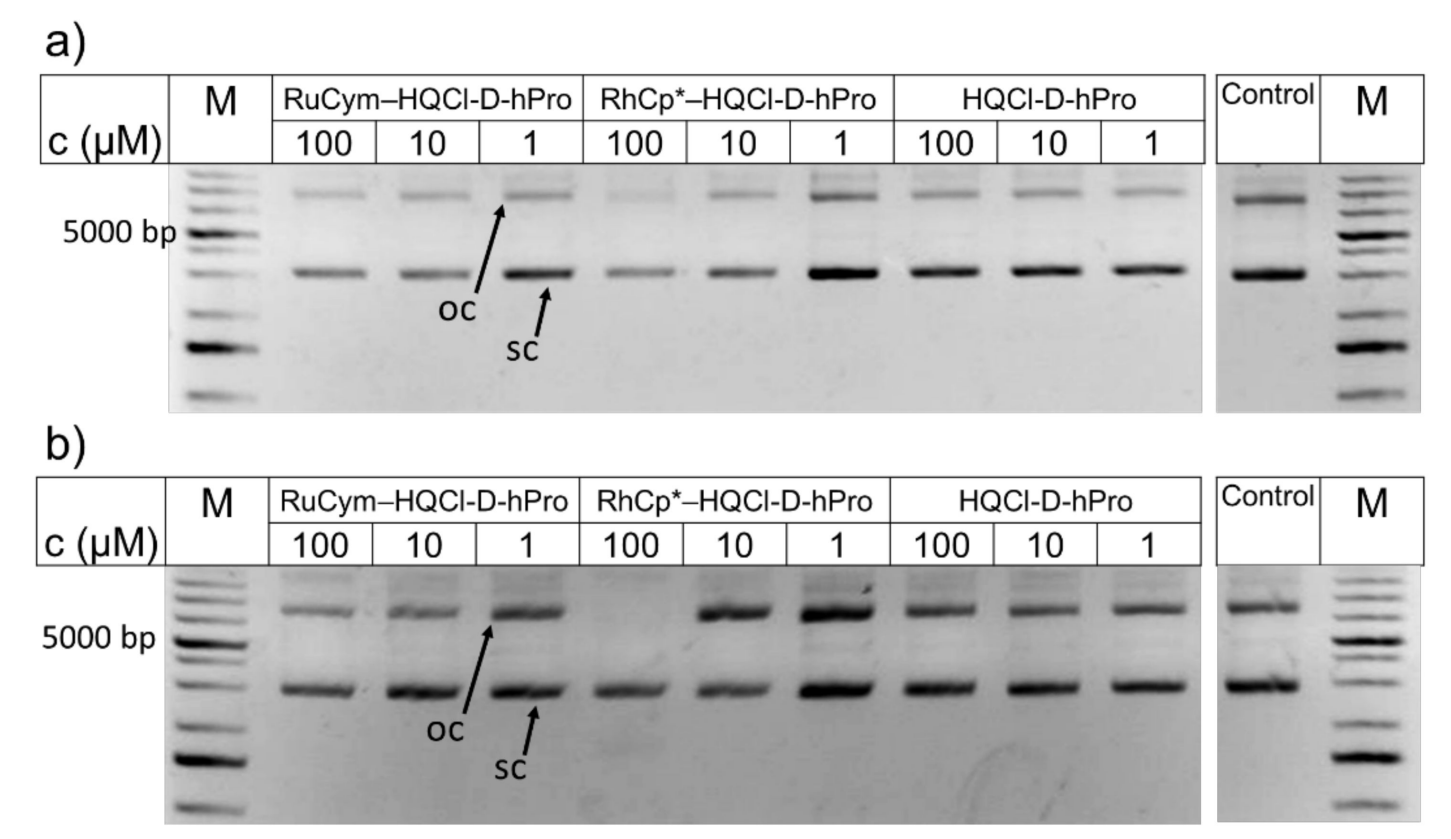
Incubation of HQCl-D-hPro ligand and its RuCym and RhCp* complexes with plasmid DNA for (**a**) 0 h, (**b**) 72 h analyzed on 1 % (*w*/*v*) agarose gel. The reaction mixtures contained 50 ng pGEX-6P-1 plasmid DNA and HQCl-D-hPro, RuCym–HQCl-D-hPro, RhCp*–HQCl-D-hPro in concentrations between 0 and 100 µM. {pH = 7.4 (20 mM phosphate buffer, 4 mM KCl); incubation time: up to 3 days; *T* = 37 °C.} Arrows indicate various forms of the plasmid: oc: open circular, sc: supercoiled. Control sample contained only plasmid DNA. 1 kb GeneRuler Plus was used as a marker (M).

In order to investigate whether HQCl-D-hPro ligand and its RhCp* and RuCym complexes result in nonspecific DNA cleavage, in vitro DNA cleavage experiments were also performed, and results are presented in Figure 13. In this assay, both single-stranded and double-stranded cleavage can be detected using bacterial plasmid DNA since the plasmid changes from the intact superhelical (sc) conformation to open circular (oc) and linear over digestion, which can be well detected by agarose gel electrophoresis. No DNA damage was observed for the ligand and its RuCym complex even after 72 h (there is a strong similarity to control). The RhCp* complex behaved similarly up to 10 µM, whereas at 100 µM concentration, the superhelical DNA remained intact during the incubation period, although the disappearance of the open circular DNA line is seen after 72 h. The observed effect is clearly not related to DNA cleavage; since fragmentation or the appearance of linear DNA could not be seen, the supercoiled DNA form was still visible. We can assume that the decrease in intensity might be due to the competition for DNA between the concentrated complex and EB (that is used for the detection of DNA in the gel). In all, we can conclude that the tested compounds clearly do not induce DNA cleavage even at higher concentrations than their IC_50_ values, so damage on naked DNA is not suggested as the mechanism of action.

## 3. Materials and Methods

### 3.1. Chemicals

All solvents were of analytical grade and used without further purification (except the solutions used for the DNA cleavage assay, *vide infra*). [RhCp*(μ-Cl)Cl]_2_, [Ru(η^6^-*p*-cymene)(μ-Cl)Cl]_2_, 5-chloro-8-hydroxyquinoline, D-proline, D-homoproline, aqueous formaldehyde (30% (*v*/*v*)), pic, phen, KNO_3_, HNO_3_, KOH, *n*-octanol, CD_3_OD, D_2_O, doxorubicin, EB, HSA (A8763, essentially globulin free), ct-DNA, DG, 4,4-dimethyl-4-silapentane-1-sulfonic acid (DSS) were Sigma-Aldrich (St. Louis, MO, USA) products and were used without further purification. FHA was purchased from GenScript (Piscataway, NJ, USA). NaH_2_PO_4_, Na_2_HPO_4_, KH_2_PO_4_, KCl, NaCl, diethyl-ether, *n*-hexane, ethanol, ethyl acetate and methanol were purchased from Molar Chemicals (Halásztelek, Hungary) or Reanal (Budapest, Hungary). [RuCym(HQCl-L-Pro)Cl]Cl and [RhCp*(HQCl-L-Pro)Cl]Cl were used in some cases due to comparative reasons, and their preparation and characterization were reported in our previous work [25].

### 3.2. Stock Solutions and Sample Preparation

Milli-Q water was used for the preparation of stock and sample solutions. The aqueous [RhCp*(H_2_O)_3_](NO_3_)_2_ and [RuCym(H_2_O)_3_](NO_3_)_2_ stock solutions were obtained by dissolving an exact amount of the dimeric precursor in water followed by the addition of equivalent amounts of AgNO_3_ and AgCl precipitate was filtered off. Determination of the concentration of the precursor and ligand stock solutions was performed by pH-potentiometry using Hyperquad2013 [46], as reported in our former work [5,6,25]. HSA stock solutions were prepared in modified phosphate-buffered saline (PBS’), which contains 12 mM Na_2_HPO_4_, 3 mM KH_2_PO_4_, 1.5 mM KCl and 100.5 mM NaCl; the concentration of K^+^, Na^+^ and Cl^−^ ions corresponds to the human blood serum. Residual citrate content of HSA was removed by repeated ultrafiltration of the protein stock solution, and its concentration was calculated from its UV absorption: λ_280 nm_ (HSA) = 36,850 M^−1^ cm^−1^ [47]. The ct-DNA stock solutions (ca. 1 mg/mL) were made by hydrating the solid ct-DNA in 20 mM phosphate buffer containing 4 mM KCl (pH = 7.40) and were left for 4 days at 4 °C. Concentrations of the ct-DNA stock solutions, expressed in nucleotide units, were determined by UV spectrophotometry using the molar absorption coefficient 6600 M^−1^ cm^−1^ at 260 nm [48]. The concentration of ct-DNA is expressed in nucleotides in this work. The ratio of UV absorbance at 260 and 280 nm was checked, and the ratio of A_260nm_/A_280nm_ ≥ 1.8 indicating that the ct-DNA was free of proteins [48]. A stock solution of EB was prepared by dissolving a known amount in water. Samples were prepared in buffer solutions to study the interaction with HSA (PBS’) and ct-DNA (20 mM phosphate buffer with 4 mM KCl) and were incubated for 24 h at 25 °C.

### 3.3. Synthesis and Characterization of Ligands and Their Complexes

#### 3.3.1. Synthesis of HQCl-D-Pro

D-Proline (1.028 g, 8.93 mmol), 5-chloro-8-hydroxyquinoline (2.000 g, 11.14 mmol), aqueous formaldehyde (30% (*v*/*v*)) (0.301 g, 10.04 mmol), and methanol (80 mL) were placed in a 250 mL round bottom flask and were refluxed at 75 °C for 6 h. The mixture was cooled down, and then it was evaporated. The residue was crystallized from 35 mL ethanol (ethanol)/ethyl acetate). Yield: 2.381 g (87%); m.p.: 190–192 °C. Specific rotation at 20 °C: ${\left[\alpha \right]}_{D}^{20}=+1.6\;$(0.5 g/mL in methanol). Purity was ≥98% as confirmed by NMR (Figures S1 and S2). ^1^H NMR (CD_3_OD, δ, ppm): 8.948 (dd, *J* = 4.17 Hz; 1.46 Hz, 1H, H(2)), 8.575 (dd, *J* = 8.57 Hz; 1.48 Hz, 1H, H(4)), 7.740 (s, 1H, H(6)), 7.705 (dd, *J* = 8.56 Hz; 4,19 Hz, 1H, H(3)), 4.592 (dd, ^2^*J* = 68.71 Hz, ^3^*J* = 13.00 Hz, 2H, H(9)), 4.054 (dd, *J* = 9.32 Hz; 5.77 Hz, 1H, H(11)), 3.625 (m, 2H, H(14)), 2.477 (m, 1H, H(12)), 2.184 (m, 1H, H(12)), 2.117 (m, 1H, H(13)), 1.956 (m, 1H, H(13)). ^13^C NMR (CD_3_OD, δ, ppm): 173.25 (C(15)), 153.62 (C(2)), 150.80 (C(8)), 140.26 (C(8a)), 134.04 (C(4)),130.16 (C(6)), 128.46 (C(5)), 124.77 (C(3)), 121.57 (C(4a)), 114.41 (C(7)), 70.40 (C(11)), 55.61 (C(14)), 54.20 (C(9)), 30.06 (C(12)), 24.43 (C(13)). ESI-MS (methanol, positive): calc. for [M + H^+^] (C_15_H_16_ClN_2_O_3_): 307.0850 (*m*/*z*) found: 307.0855 (*m*/*z*).

#### 3.3.2. Synthesis of HQCl-D-hPro

D-Homoproline (1.153 g, 8.93 mmol), 5-chloro-8-hydroxyquinoline (2.000 g, 11.14 mmol), aqueous formaldehyde (30% (*v*/*v*)) (0.301 g, 10.04 mmol) and methanol (80 mL) were placed in a 250 mL round bottom flask. The mixture was refluxed at 75 °C for 6 h, then cooled down. The mixture was evaporated, and the residue was crystallized from 35 mL ethyl acetate, then recrystallized from 25 mL ethanol. Yield: 1.020 g (36%); m.p.: 194–197 °C. ${\left[\alpha \right]}_{D}^{20}=-4.6\;$(1.0 g/mL in methanol). Purity was ≥ 98% as confirmed by NMR (Figures S3 and S4). ^1^H NMR (CD_3_OD, δ, ppm): 8.951 (dd, *J* = 4.20 Hz; 1.47 Hz, 1H, H(2)), 8.589 (d, *J* = 8.56 Hz; 1.49 Hz, 1H, H(4)), 7.822 (s, 1H, H(6)), 7.717 (m, *J* = 8.56 Hz; 4.20 Hz, 1H, H(3)), 4.575 (d, *J* = 68.20 Hz, **^3^***J* = 12.95, 1H, H(9)), 3.604 (dd, *J* = 10.76 Hz; 3.65 Hz, 1H, H(11)), 3.479 (m, 1H, H(15)), 3.087 (td, J = 12.28 Hz; 3.09 Hz, 1H, H(15)), 2.295 (m, 1H, H(12)), 1.79–1.91 (m, 3H, H(12+13)), 1.67–1.75 (m, 1H, H(14)), 1.53–1.62 (m, 1H, H(14)). ^13^C NMR (CD_3_OD, δ, ppm): 173.69 (C(16)), 154.07 (C(8)), 150.75 (C(2)), 140.30 (C(8a)), 134.08 (C(4)), 130.75 (C(6)), 128.48 (C(5)), 124.83 (C(3)), 121.51 (C(4a)), 113.58 (C(7)), 68.83 (C(11)), 54.55 (C(15)), 52.23 (C(9)), 28.98 (C(12)), 23.44 (C(14)), 22.69 (C(13)). ESI-MS (methanol, positive): calc. for [M + H^+^] (C_16_H_18_ClN_2_O_3_): 321.1006 (*m*/*z*) found: 321.1014 (*m*/*z*).

#### 3.3.3. Synthesis of [RuCym(HQCl-D-Pro)Cl]Cl

HQCl-D-Pro ligand (5.0 mg, 16.3 µmol) and [RuCymCl_2_]_2_ (5.0 mg, 8.15 µmol) were dissolved in methanol (800 µL) and stirred at room temperature for 24 h, then the solution was concentrated. Precipitation was carried out by addition of diethyl ether to the mixture. The formed orange solid was filtered and washed with diethyl ether and *n*-hexane. The same synthetic route was applied for the other three complexes. Yield: 8.02 mg (80%). ^1^H NMR (CD_3_OD, δ, ppm, Figure S7): 9.358 (t, *J* = 4.25 Hz, 1H, H_lig_(2)), 8.519 (d, *J* = 8.65 Hz, 1H, H_lig_(4)), 7.722 (m, 1H, H_lig_(3)), 7.518 (s, 0.38 H, H_lig_(6)), 7.448 (s, 0.60 H, H_lig_(6)), 5.847–5.901 (m, 2H, H_cym_(C3)), 5.597–5.665 (m, 2H, H_cym_(C2)), 4.851 (d, *J* = 13.21 Hz, 1H, H_lig_(9)), δ 4.627–4.647 (m, 0.77H, H_lig_(9 and 11)), 4.518 (d, *J* = 12.98 Hz, 1H, H_lig_(9)), 4.290 (d, *J* = 13.25 Hz, 1H, H_lig_(9)), 4.392 (m, *J* = 9.40 Hz; 5.79 Hz, 0.63H, H_lig_(11)), 3.490–3.581 (m, 1H, H_lig_(14)), 3.060 (m, *J* = 19.12 Hz; 8.88 Hz, 0.65H, H_lig_(14)), 2.872 (m, *J* = 6.91 Hz, 0.67 H, H_cym_(C5)), 2.810 (m, *J* = 6.91 Hz, 0.33 H, H_cym_(C5)), 2.515–2.611 (m, 1H, H_lig_(12)), 2.304 (m, 1H, H_lig_(12)), 2.258 (d, *J* = 5.08 Hz, 3H, H_cym_(C8)), 2.116 (m, 1H, H_lig_(13)), 1.949 (m, 1H, H_lig_(13)), 1.194 (m, 6H, H_cym_(C6+C7)). ^13^C NMR (CD_3_OD, δ, ppm, Figure S8): 171.88 (C(15)), 168.71 (C(8)), 167.64 (C(8)), 153.03 (C(2)), 152.84 (C(2)), 145.97 (C(8a)), 145.30 (C(8a)), 135.85 (C(4)), 135.79 (C(4)), 132.10 (C(6)), 130.61 (C(6)), 129.70 (C(5)), 129.29 (C(5)), 125.62 (C(3)), 125.48 (C(3)), 115.93 (C(4a)), 115.18 (C(4a)), 114.39 (C(7)), 114.00 (C(7)), 103.43; 103.20 (C(C4)), 100.26; 99.82 (C(C1)), 84.08; 83.90; 83.45; 83.43; 82.92; 82.63; 82.17; 81.95 (C(C2+C3)), 68.32 (C(11)), 67.84 (C(11)), 57.19 (C(14)), 55.36 (C(9)), 54.82 (C(9)), 54.05 (C(9)), 32.31 (C(C5)), 32.25 (C(C5)), 30.02 (C(12)), 29.12 (C(12)), 24.20 (C(13)), 23.61 (C(13)), 22.52; 22.50; 22.35; 22.30 (C(C6+C7)), 18.76 (C(8)), 18.70 (C(C8)). ESI-MS (methanol, positive): calc. for [RuCym(HQCl-D-Pro)H_−1_]^+^ (C_25_H_28_ClN_2_O_3_Ru): 541.0832 (*m*/*z*) found: 541.0815 (*m*/*z*) and [RuCym(HQCl-D-Pro)]^2+^ (C_25_H_29_ClN_2_O_3_Ru): 271.0455 (*m*/*z*) found 271.0454 (*m*/*z*).

#### 3.3.4. Synthesis of [RhCp*(HQCl-D-Pro)Cl]Cl

The synthesis was the same as it was described for [RuCym(HQCl-D-Pro)Cl]Cl. HQCl-D-Pro (5.0 mg, 16.3 µmol), [RhCp*Cl_2_]_2_ (5.0 mg, 8.15 µmol). Yield: 8.40 mg (84%). ^1^H NMR (CD_3_OD, δ, ppm, Figure S5): 8.946 (d, J = 4.52 Hz, 1H, H_lig_(2)), 8.561 (d, *J* = 8.48 Hz, 1H, H_lig_(4)), 7.774 (m, *J* = 8.59 Hz; 4.88 Hz, 1H, H_lig_(3)), 7.549 (s, 0.34 H, H_lig_(6)), 7.480 (s, 0.66 H, H_lig_(6)), 4.949 (d, *J* = 13.04 Hz, 0.29 H, H_lig_(9)), 4.683 (d, *J* = 14.17 Hz, 0.23 H, H_lig_(9)), 4.511 (d, *J* = 12.60 Hz, 0.18 H, H_lig_(9)), 4.206 (d, *J* = 12.96 Hz, 0.30 H, H_lig_(9)), 4.408 (t, *J* = 7.61 Hz, 1H, H_lig_(11)), 3.52–3.62 (m, 1H, H_lig_(14)), 3.065 (dd, *J* = 18.34 Hz; 8.65 Hz, 1H, H_lig_(14)), 2.50–2.63 (m, 1H, H_lig_(12)), 2.24–2.34 (m, 0.7H, H_lig_(12)), 2.05–2.20 (m, 1H, H_lig_(13)), 1.90–2.00 (m, 1H, H_lig_(13)), 1.651 (m, 1H, H_lig_(13)), 1.775 (s, 15H, H_C5Me5_(CH_3_)). ^13^C NMR (CD_3_OD, δ, ppm, Figure S6): 150.15 (C(2)), 136.07 (C(4)), 131.16 (C(6)), 129.76 (C(5)), 125.64 (C(3)), 115.98 (C(4a)), 113.84 (C(7)), 113.49 (C(7)), 95.96 (C(C_5_)), 95.90 (C(C_5_)), 68.64 (C(11)), 67.67 (C(11)), 57.55 (C(14)), 55.15 (C(9)), 55.00 (C(9)), 29.93 (C(12)), 29.25 (C(12)), 23.99 (C(13)), 8.96 (C(Me_5_)). ESI-MS (methanol, positive): calc. for [RhCp*(HQCl-D-Pro)H_−1_]^+^ (C_25_H_29_ClN_2_O_3_Rh): 543.0922 (*m*/*z*) found: 543.0926 (*m*/*z*) and [RhCp*(HQCl-D-Pro)]^2+^ (C_25_H_30_ClN_2_O_3_Rh): 272.0500 (*m*/*z*) found 272.0501 (*m*/*z*).

#### 3.3.5. Synthesis of [RuCym(HQCl-D-hPro)Cl]Cl

The synthesis was the same as it was described for [RuCym(HQCl-D-Pro)Cl]Cl. HQCl-D-hPro (5.0 mg, 15.6 µmol), [RuCymCl_2_]_2_ (4.79 mg, 7.8 µmol). Yield: 7.90 mg (81%). ^1^H NMR (CD_3_OD, δ, ppm, Figure S11): 9.358 (d, *J* = 4.94 Hz, 1H, H_lig_(2)), 8.518 (dd, *J* = 9.97 Hz; 1.90 Hz, 1H, H_lig_(4)), 7.735 (ddd, *J* = 8.62 Hz; 5.03 Hz; 2.77 Hz, 1H, H_lig_(3)), 7.526 (s, 1H, H_lig_(6)), 7.450 (s, 1H, H_lig_(6)), 5.83–5.88 (m, 2H, H_cym_(C3)), 5.59–5.65 (m, 2H, H_cym_(C2)), 4.887 (d, *J* = 13.17 Hz, 0.6H, H_lig_(9)), 4.392 (d, *J* = 13.26 Hz, 0.4H, H_lig_(9)), 4.28–4.32 (m, 1H, H_lig_(9+11)), 3.987 (dd, *J* = 9.89 Hz; 3.69 Hz, 0.4H, H_lig_(11)), 3.47–3.51 (m, 0.6H, H_lig_(15)), 3.38–3.43 (m, 0.4H, H_lig_(15)), 3.138 (t, *J* = 11.88 Hz, 0.4H, H_lig_(15)), 2.896 (t, *J* = 11.18 Hz, 0.6H, H_lig_(15)), 2.75–2.86 (m, 1H, H_cym_(C5)), 2.354 (m, 1H, H_lig_(12)), 1.978 (m, 0.5H, H_lig_(12)), 2.259 (d, *J* = 4.61 Hz, 3H, H_cym_(C8)), 1.90–1.52 (m, 3.5H, H_lig_(12+13+14)), 1.209–1.152 (m, 6H, H_cym_(C6+C7)). ^13^C NMR (CD_3_OD, δ, ppm, Figure S12): 172.07 (C(16)), 171.66 (C(16)), 169.16 (C(8)), 168.28 (C(8)), 152.99 (C(2)), 152.66 (C(2)), 146.09 (C(8a)), 145.61 (C(8a)), 135.85 (C(4)), 135.74 (C(4)), 132.57 (C(6)), 131.71 (C(6)), 129.77 (C(5)), 129.52 (C(5)), 125.61 (C(3)), 114.52 (C(4a)), 114.47 (C(4a)), 113.82 (C(7)), 103.17 (C(C4)), 102.89 (C(C4)), 100.33 (C(C1)), 100.21 (C(C1)), 84.21; 83.62; 83.40; 82.63; 81.86 (C(C2+C3)), 65.29 (C(11)), 64.95 (C(11)), 56.40 (C(15)), 55.08 (C(15)), 52.49 (C(9)), 52.15 (C(9)), 32.29 (C(C5)), 28.22 (C(12)), 27.97 (C(12)), 22.93 (C(14)), 22.70 (C(14)), 22.54; 22.46; 22.32 (C(C6+C7)), 22.13 (C(13)), 21.99 (C(13)), 18.76 (C(C8)). ESI-MS (methanol, positive): calc. for [RuCym(HQCl-D-hPro)H_−1_]^+^ (C_26_H_30_ClN_2_O_3_Ru): 555.0989 (*m*/*z*) found: 555.0959 (*m*/*z*) and [RuCym(HQCl-D-hPro)]^2+^ (C_26_H_31_ClN_2_O_3_Ru): 278.0534 (*m*/*z*) found 278.0522 (*m*/*z*) and [RuCym(HQCl-D-hPro)(Cl)]^+^ (C_26_H_31_Cl_2_N_2_O_3_Ru): 591.0755 (*m*/*z*) found: 591.0729 (*m*/*z*).

#### 3.3.6. Synthesis of [RhCp*(HQCl-D-hPro)Cl]Cl

The synthesis was the same as it was described for [RuCym(HQCl-D-Pro)Cl]Cl. HQCl-D-hPro (5.0 mg, 15.6 µmol), [RhCp*Cl_2_]_2_ (4.79 mg, 7.8 µmol). Yield: 7.70 mg (79 %). ^1^H NMR (CD_3_OD, δ, ppm, Figure S9): 8.948 (d, *J* = 4.44 Hz, 1H, H_lig_(2)), 8.563 (d, *J* = 8.43 Hz, 1H, H_lig_(4)), 7.780 (m, *J* = 8.52 Hz; 4.88 Hz, 1H, H_lig_(3)), 7.548 (s, 0.40 H, H_lig_(6)), 7.464 (s, 0.60 H, H_lig_(6)), 4.907 (d, *J* = 13.18 Hz, 1H, H_lig_(9)), 4.371 (d, *J* = 12.37 Hz, 1H, H_lig_(9)), 4.243 (d, *J* = 12.98 Hz, 1H, H_lig_(9)), 4.061 (d, *J* = 11.80 Hz, 1H, H_lig_(11)), 3.600 (m, 0.6H, H_lig_(15)), 3.374 (d, *J* = 11.97 Hz, 0.4H, H_lig_(15)), 3.051 (t, *J* = 22.83 Hz, 1H, H_lig_(15)), 2.10–2.41 (m, 2H, H_lig_(12)), 1.963–1.644 (m, 4H, H_lig_(13+14)), 1.768 (s, 15H, H_C5Me5_(CH_3_)). ^13^C NMR (CD_3_OD, δ, ppm, Figure S10): 171.83 (C(16)), 171.54 (C(16)), 150.39 (C(2)), 150.04 (C(2)), 136.11 (C(4)), 136.00 (C(4)), 132.78 (C(6)), 131.81 (C(6)), 129.83 (C(5)), 125.79 (C(3)), 114.59 (C(4a)), 114.39 (C(7)), 114.08 (C(7)), 95.87 (C(C_5_)), 65.72 (C(11)), 65.01 (C(11)), 57.41 (C(15)), 55.70 (C(15)), 52.39 (C(9)), 28.83 (C(12)), 23.47 (C(13)), 22.18 (C(14)), 21.96 (C(14)), 8.97 (C(Me_5_)). ESI-MS: calc. for [RhCp*(HQCl-D-hProH_−1_)]^+^ (C_26_H_31_ClN_2_O_3_Rh): 557.1078 (*m*/*z*) found: 557.1054 (*m*/*z*) and [RhCp*(HQCl-D-hPro)]^2+^ (C_26_H_32_ClN_2_O_3_Rh): 279.0578 (*m*/*z*) found 279.0568 (*m*/*z*).

### 3.4. Electrospray Mass Spectrometry 

High-resolution ESI-MS experiments were performed by a Waters Q-TOF Premier (Micromass MS Technologies, Manchester, UK) mass spectrometer with an electrospray ion source. Samples contained 100 µM compounds (ligand or complex) in methanol (LC-MS grade).

### 3.5. pH-Potentiometric Measurements

pH-potentiometric measurements were carried out at 25.0 ± 0.1 °C in water in the pH range between 2.0 and 11.5 and at a constant ionic strength of 0.2 M KNO_3_. The titrations were performed in a carbonate-free KOH solution (0.20 M). The exact concentrations of HNO_3_ and KOH solutions were determined by pH-potentiometric titrations. An Orion 710A pH-meter equipped with a Metrohm combined electrode (type 6.0234.100) and a Metrohm 665 Dosimat burette were used for the pH-potentiometric measurements. The electrode system was calibrated according to the method suggested by Irving et al. [49]. The average water ionization constant, p*K*_w_, was determined as 13.76 ± 0.01, which is in good agreement with the literature data [50]. The initial volume of the samples was 5.0 mL. The ligand concentration was 1.75 or 2.0 mM. Samples were degassed by bubbling purified argon through them for about 10 min prior to the measurements, and the inert gas was also passed over the solutions during the titrations. The computer program Hyperquad2013 [46] was utilized to establish the stoichiometry of the species and to calculate the equilibrium constants, similarly to that performed in our previous works [5,6,9,25,32].

### 3.6. UV-Visible Spectrophotometry and Determination of Distribution Coefficients

Agilent Cary 8454 diode array spectrophotometer was utilized to obtain UV-vis spectra in the wavelength range 190–1100 nm. The path length (*ℓ*) was 1 cm in most cases (the actual *ℓ* is always indicated in the legends of the figures). The concentrations of the ligands and complexes were between 88 and 240 µM. For HSA and ct-DNA containing samples, complex concentrations were 100 µM, and 0.5 and 2 equiv. biomolecule were added for kinetic studies, respectively. Individual samples contained 0–20 µM HSA and 0–1.75 mM (nucleotide) ct-DNA. Spectra were always background corrected. The computer program HypSpec [46] was used to obtain stability constants.

Distribution coefficients of the ligands as well as of the complexes were determined at pH = 7.4 using the traditional shake-flask method in *n*-octanol/buffered aqueous solution (20 mM phosphate) at different chloride ion concentrations (4, 24 and 100 mM) using UV-vis spectrophotometry for the analysis. The measurement and data evaluation were performed as described in our former work [24].

### 3.7. Circular Dichroism Spectroscopy and Spectrofluorometry

CD spectroscopy experiments were performed by a JASCO-J-1500 (ABL&E–JASCO Hungary Ltd., Budapest, Hungary) spectrometer using 0.2 or 1 cm optical path length in the wavelength range from 200 to 250 or to 350 nm. The scanning speed was 200 nm/min. The analytical concentration of HSA and ct-DNA was constant (2 and 98 µM, respectively), and the concentration of the complexes was varied (0–26 and 0–47 µM, respectively).

Fluorescence measurements were carried out on a Fluoromax (Horiba Jobin Yvon, Longjumeau, France) spectrofluorometer using 1 cm × 1 cm quartz cuvette. Samples contained 1 µM HSA or 1 µM DG and 1 µM HSA, or 20 µM (nucleotide) ct-DNA and 5 µM EB, and the complex concentration was varied between 0 and 40 µM or 0 and 300 µM, respectively. Spectroscopic measurements were carried out on individually prepared samples. Excitation wavelengths were 295 nm for Trp-214 quenching, 340 nm for the DG displacement studies and 510 nm for the ct-DNA–EB system. The calculated conditional stability constants for HSA–complex species were obtained using the computer program HypSpec [46]. Calculations always were based on data obtained from at least two independent measurements. Self-absorbance and inner filter effect had to be taken into account [51], and corrections were made as was described in our former works [34,35].

Time-resolved fluorometric measurements for the DNA–EB system were implemented on the same fluorometer in addition to DeltaHub time-correlated single-photon counting (TCSPC) controller using NanoLED light source N-460 (455) (Horiba Jobin Yvon). The time resolution was 25 ps and 50 ps for measurements in time windows of 100 ns and 200 ns, respectively. Decay curves were recorded at λ_EM_ = 610 nm with a slit width of 5 nm; the count number at peak channel was set to 10,000, and approximately 2500 channels were used for analysis. Instrument response function (IRF) was measured using Ludox^®^ (Sigma-Aldrich) as a scatter solution; background correction was not necessary. The computer program DAS6 (version 6.6.; Horiba Jobin Yvon) was used for the analysis of fluorescence decay curves. The fluorescence intensity decay over time is described by a sum of exponentials,

(1) $$I\left(t\right)=\overset{n}{\underset{i=1}{\sum }}\alpha_{i}exp\left(\frac{-t}{\tau_{i}}\right)$$

where *α_i_* and *τ_i_* are the normalized amplitude and lifetime of component *i*, respectively, and *t* is the time [51]. From these parameters, the fraction of emitted light (*f*) by each component *i* can be calculated through Equation (2).

(2) $$f_{i}=\frac{\alpha_{i}\tau_{i}}{\sum \left(\alpha_{i}\tau_{i}\right)}$$

The sum of fractional intensities gives the amplitude-weighted lifetime that is often referred to as the average lifetime (*τ*_ave_) of the system. The quality of the fit was judged from a χ^2^_R_ value close to 1.0 (χ^2^_R_ ≤ 1.20) and a random distribution of weighted residuals. 

### 3.8. NMR Spectroscopy

Bruker Avance III HD Ascend 500 Plus instrument (Billerica, MA, USA) was used for NMR studies. ^1^H NMR spectroscopic measurements were carried out with a WATERGATE water suppression pulse scheme in the presence of 10% (*v*/*v*) D_2_O in most cases. DSS internal standard was added to samples to obtain reference peaks. Complex concentrations were 0.5 mM or 1.0 mM, and 0.5 equiv. HSA was added. ^1^H NMR titrations were carried out in the presence of 0.2 M KNO_3_. With the FHA model peptide, the measurements were performed at 0.5 or 1 mM metal complex concentrations, and 1:1, 1:2 or 2:1 peptide-to-metal complex ratios were applied. The computer program HypSpec [46] was used to obtain stability constants. 

For ligand and complex characterization, ^13^C NMR spectra were recorded in CD_3_OD (10 mM) with the attached proton test (APT) showing CH and CH_3_ as positive peaks, while quaternary C and CH_2_ appear as negative peaks.

### 3.9. Capillary Zone Electrophoresis

Agilent 7100 capillary electrophoresis system equipped with diode-array UV-vis detector (200–600 nm) was utilized to gain electropherograms. Fused silica capillaries of 48 cm total length with 50 or 75 µm inner diameter were used (Agilent Technologies, Santa Clara, CA, USA). The background electrolyte (BGE) was PBS buffer (pH = 7.40), in which the samples were also made. The conditioning process of new capillaries and daily preparation were performed as described formerly [34]. In order to ensure the steady baseline, the capillary was flushed with BGE (2 min) before each run and was rinsed with NaOH (0.1 M; 1.5 min), H_2_O (1.5 min), and then with BGE (2 min) after each separation. The sample tray and the capillary cassette were kept at 25 °C. The hydrodynamic injection was used at 100 mbar for 5 s injection time. For separation, 25 kV voltage was applied to produce a current of ca. 80 or 100 μA. The sample run time was set to 15 min. The computer program ChemStation (Agilent) was used to record electropherograms. Electropherograms and spectra of the corresponding peaks were also collected. In order to check the purity of the isolated organometallic complexes, measurements were performed at 0.5 mM compound concentration with the corresponding ligands and precursors as well. Interaction with HSA was studied at constant protein concentration (50 μM), and the HSA-to-complex ratio was varied between 0 and 0.25. In order to calculate the non-bound complex concentration, external calibration was used based on peak integrals.

### 3.10. X-ray Data Collection, Structure Solution and Refinement for Compound [RhCp*(HQCl-D-hPro)Cl]Cl∙H_2_O∙CH_3_OH

Single crystals suitable for X-ray diffraction experiment of compound [RhCp*(HQCl-D-hPro)Cl]Cl∙H_2_O∙CH_3_OH were grown from methanol with the slow diffusion of diethyl ether at room temperature. A platelet-shaped yellow single crystal was mounted on a loop and transferred to the goniometer. X-ray diffraction data were collected at 140(2) K on a Rigaku RAXIS-RAPID II diffractometer using Mo-*Kα* radiation. Numerical absorption correction [52] was carried out using the program CrystalClear (2008, Rigaku/MSC Inc., Austin, TX, USA) [53]. SHELX (2018/3, University of Göttingen, Göttingen, Germany) [54] program package under WinGX (2020.1, University Of Glasgow, Glasgow, Scotland) [55] software was used for structure solution and refinement. The structures were solved by direct methods. The models were refined by full-matrix least-squares on F^2^. Refinement of non-hydrogen atoms was carried out with anisotropic temperature factors. In the refinement process, the hydrogen atoms were placed into geometric positions and were included in structure factor calculations as riding. The isotropic displacement parameters of the hydrogen atoms were approximated from the U(eq) value of the atom they were bonded to. One water of crystallization was found in two disordered positions with an occupancy ratio of 70/30. Its hydrogen atoms could not be detected. The summary of data collection and refinement parameters are collected in Table S1. Selected bond lengths and angles of compounds were calculated by PLATON (2020.2, Universitiy of Utrecht, Utrecht, Netherlands) software [56]. The graphical representation and the addition of CIF files were created by using the Mercury (2021.1, Cambridge Crystallographic Data Centre, Cambridge, UK) [57] and EnCIFer (2021.1, Cambridge Crystallographic Data Centre, Cambridge, UK) [58] software.

### 3.11. In Vitro Cell Studies

#### 3.11.1. Cell Lines and Culture Conditions

All cell culture reagents were obtained from Sigma-Aldrich and plasticware from Sarstedt (Nümbrecht, Germany). Human colonic Colo-205 doxorubicin-sensitive and Colo-320/MDR-LRP multidrug-resistant adenocarcinoma and MRC-5 human embryonal lung fibroblast cell lines were purchased from LGC Promochem, Teddington, UK. The colonic adenocarcinoma cells were cultured in Roswell Park Memorial Institute (RPMI) 1640 medium supplemented with 10% heat-inactivated fetal bovine serum, 2 mM L-glutamine, 1 mM sodium pyruvate and 100 mM HEPES. The MRC-5 cell line was cultured in Eagle’s Minimal Essential Medium (EMEM, Sigma-Aldrich, St. Louis, MO, USA) supplemented with a non-essential amino acid (NEAA) mixture (Sigma-Aldrich, St. Louis, MO, USA) a selection of vitamins, 10% heat-inactivated foetal bovine serum (FBS), 2 mM L-glutamine (Sigma-Aldrich, St. Louis, MO, USA), 1 mM Na-pyruvate (Sigma-Aldrich, St. Louis, MO, USA), nystatin (Sigma-Aldrich, St. Louis, MO, USA) and a penicillin-streptomycin mixture (Sigma-Aldrich, St. Louis, MO, USA) in con-centrations of 100 U/L and 10 mg/L, respectively. The cells were incubated at 37 °C, in a 5% CO_2_, 95% air atmosphere. All cell lines were detached with Trypsin-Versene (EDTA) solution for 5 min at 37 °C.

#### 3.11.2. MTT Assay

The tested compounds were dissolved in 10% (*v*/*v*) DMSO/H_2_O using 4 mM concentration. Doxorubicin (Merck, Darmstadt, Germany) was used as a positive control. Then stock solutions were diluted in complete culture medium, and two-fold serial dilutions of compounds were prepared in 100 μL of the medium, horizontally. The semi-adherent colonic adenocarcinoma cells were treated with Trypsin-Versene (EDTA) solution. They were adjusted to a density of 1 × 10^4^ cells in 100 μL of RPMI 1640 or EMEM medium and were added to each well, with the exception of the medium control wells. The final volume of the wells containing compounds and cells was 200 μL. The plates containing Colo-205, Colo-320 or MRC-5 cells were incubated at 37 °C for 72 h; at the end of the incubation period, 20 μL of MTT solution (from a stock solution of 5 mg/mL) were added to each well. After incubation at 37 °C for 4 h, 100 μL of SDS solution (10% in 0.01 M HCI) were added to each well, and the plates were further incubated at 37 °C overnight. Cell growth was determined by measuring the optical density (OD) at 540/630 nm with a Multiscan EX ELISA reader ((Thermo Labsystems, Cheshire, WA, USA). Inhibition of the cell growth (expressed as IC_50_: inhibitory concentration that reduces by 50% the growth of the cells exposed to the tested compounds) was determined from the sigmoid curve where 100 − ((OD_sample_ − OD_medium control_)/(OD_cell control_ − OD_medium control_)) × 100 values were plotted against the logarithm of compound concentrations. Curves were fitted by GraphPad Prism software (2021, Graphpad Software, San Diego, CA, USA) [59] using the sigmoidal dose–response model (comparing variable and fixed slopes). The IC_50_ values were obtained from at least 3 independent experiments.

#### 3.11.3. Assay for Apoptosis Induction

The assay was carried out using an Annexin V-FITC Apoptosis Detection Kit from Sigma-Aldrich according to the manufacturer’s instructions. The concentration of the Colo-205 cell suspension was adjusted to approximately 0.5 × 10^6^ cells per mL in RPMI 1640 medium, and the cell suspension was distributed in 1 mL aliquots into a 24-well plate and then incubated overnight at 37 °C and 5% CO_2_. The next day, the medium was removed and replaced by 1 mL RPMI 1640 medium containing the compounds except for the control samples. Colo-205 cells were incubated in the presence of the compounds at 25 μM in the 24-well plate at 37 °C for 3 h, and 12H-benzo[α]phenothiazine (M627) and cisplatin (Teva, Petah Tikva, Israel) at 15 μM were used as positive controls. After the incubation period, the samples were washed with PBS, and a fresh medium was added to the samples. The cells were incubated overnight at 37 °C and 5% CO_2_. The next day, 200 μL 0.25% Trypsin (Trypsin-Versen) was added to the samples until cells appeared detached, followed by the addition of 400 μL of medium supplemented with 10% bovine serum. The cells were collected in Eppendorf tubes and centrifuged at 2000× *g* for 2 min. The harvested cells were re-suspended in a fresh serum-free RPMI 1640 culture medium. After this step, the apoptosis assay was carried out according to the protocol of the kit. The fluorescence was analyzed immediately using a ParTec CyFlow flow cytometer (Partec GmbH, Münster, Germany).

### 3.12. In Vitro DNA Cleavage Analysis

Interaction of free HQCl-D-hPro ligand and its RuCym and RhCp* complexes with the pGEX-6P-1 plasmid DNA (Sigma Aldrich) were examined in 20 mM phosphate buffer with 4 mM KCl (pH = 7.4). Before the measurements, the reaction buffer was treated with 5 mg/L Chelex-100 (Sigma-Aldrich) cation exchange resin for 30 min at 25 °C. This was followed by filtration of the buffer through 0.2 µm pore size sterile, non-pyrogenic/endotoxin-free non-cytotoxic PES filter (Sarstedt) under laminal flow. The tested compounds were dissolved in this filtered cation exchanged buffer. pGEX-6P-1 DNA was purified by GeneJET plasmid maxiprep kit (Thermo Scientific™, Waltham, MA, USA) and was treated with Chelex-100 resin and filtered as described previously, and was followed by buffer exchange of the DNA sample using Amicon 10K 0.5 mL filters (4 × 5 min, 14,000 *g*, 25 °C) to the filtered reaction buffer. The ratio of UV absorbance at 260 and 280 nm was checked, and the ratio of A_260nm_/A_280nm_ = 1.82, indicating that the DNA was free of proteins and RNA. Based on agarose gel electrophoresis, no genomic DNA contamination was detected, and the plasmid DNA is largely in superhelical conformation with a small fraction of open circular form. In a typical reaction, 10 µL aliquots containing 50 ng DNA and 0–100 µM compound were incubated at 37 °C from 0 h up to 3 days. The reaction mixture was immediately examined after incubation or frozen at −30 °C until the gel electrophoresis experiment. The reaction mixtures were separated on 1% (*w*/*v*) agarose gels containing 500 ng/mL EB at 100 V for 45 min (Cleaver Scientific MultiSUB Wide Midi, Rugby, UK). GeneRuler™ 1kb Plus DNA Ladders (Thermo Scientific) served as reference. Images were recorded by a Uvitec BTS 20MS gel documentation system.

## 4. Conclusions

Two novel 8-hydroxyquinoline-D-amino acid hybrids (HQCl-D-Pro, HQCl-D-hPro) and their half-sandwich RuCym and RhCp* complexes were synthesized and characterized in order to obtain MDR-selective anticancer compounds with increased water solubility. Impact of the chirality of the amino acid residue (L vs. D) and the substitution of Pro fragment with hPro was investigated on the solution chemical properties of the novel compounds in addition to their cytotoxicity and selectivity to normal and MDR cells. These D-amino acid hybrids showed excellent solubility due to their zwitterionic structures in a wide pH range based on the determined p*K*_a_ values and exhibited significant cytoxicity (IC_50_ = 13–21 μM) against the tested two human colon adenocarcinoma cell lines (Colo-205 and Colo-320). Both 8-hydroxyquinoline derivatives were more cytotoxic against the MDR cells than the sensitive ones, most probably due to the presence of the CH_2_–N building block at position seven on the 8-hydroxyquinoline scaffold. The RhCp* complexes displayed similar cytotoxicity as their corresponding ligands with improved selectivity to the sensitive cancer cells over the non-tumoral human lung fibroblast cells; however, they did not show MDR selective toxicity. The presence of the additional methylene moiety in the HQCl-D-hPro ligand proved to be beneficial for the anticancer effect as its RhCp* complex was more cytotoxic than that of the HQCl-D-Pro. Meanwhile, the RuCym complexes were inactive (IC_50_ > 100 μM) against the tested cancer cell lines. Comparing the population of the early and late apoptotic and necrotic cells determined for HQCl-D-hPro and its RhCp* and RuCym complexes by apoptosis assay, it was found that the compounds were not able to induce early apoptosis, and a lower percentage of the late apoptotic and necrotic cells were obtained for the RuCym complex than for the more cytotoxic ligand and RhCp* complex.

The RhCp* and RuCym complexes of the title 8-hydroxyquinoline-D-amino acid hybrids possess quite high stability in solution based on the solution speciation data obtained by the combined use of UV-vis and ^1^H NMR spectroscopy. Negligible complex dissociation could be observed even at very acidic pH, such as pH 0.7 (≤3% at 0.5 mM concentration). The complex formation is significantly slower in the case of the RuCym complexes. These ligands coordinate to the metal center via (N,O^−^) donor set, which was confirmed by X-ray crystallography for the isolated complex [RhCp*(HQCl-D-hPro)Cl]Cl. Interestingly, an intramolecular hydrogen bond located between the protonated homoproline nitrogen and the coordinated O^−^ atom further stabilizes the coordination of the ligand. The p*K*_a_ values determined for the complexes revealed that the complexes are present predominantly in their [M(arene)(HQCl-D-(h)ProH_−1_)(H_2_O)]^+^ form in aqueous solution at physiological pH in the absence of chloride ions. In this species, the amino acid moiety of the ligand is in its zwitterionic form (N_Pro_H^+^, COO^−^) and the coordinated aqua ligand deprotonates only in the basic pH range in all cases (p*K*_a_ ≥ 8.77), and the RuCym complexes were characterized by lower p*K*_a_ values in comparison to the RhCp* species. The aqua ligand can be partly or completely substituted by a chlorido ligand in the presence of chloride ions, and the RhCp* complexes have stronger chloride ion affinity as their log*K*′ (H_2_O/Cl^−^) constants are higher by ca. 0.5 logarithm unit compared to those of the RuCym complexes. The coordination of the chloride ion increases the lipophilicity of the complexes due to the formation of a higher fraction of neutral [M(arene)(HQCl-D-(h)ProH_−1_)(Cl)] species. The homoproline hybrids are more lipophilic than the proline complexes. Although the RuCym compounds are more lipophilic than the analogous RhCp* complexes, they displayed much weaker cytotoxicity. The inactivity of the RuCym complexes is thought to be due to their tendency to lose the arene ring.

The UV-vis and capillary zone electrophoresis studies showed strong binding of the complexes to HSA without the release of the bidentate ligand. The homoproline-bearing complexes bind to this protein stronger than the proline complexes, and a somewhat weaker binding was found for the complexes bearing amino acids with D-configuration. Histidine-containing tripeptides could serve as good binding models for surface-exposed side chains, and based on their interaction with the complexes, we suggest that most probably histidine imidazoles of HSA coordinate to the metal ions at the third position by the replacement of the co-ligand (Cl^−^ or H_2_O). The complexes bind to both hydrophobic binding pockets of HSA (site I and II), most probably via coordinative bonds. The α-helix content of HSA was only slightly changed upon the binding of the complexes. The studied half-sandwich complexes are able to interact with ct-DNA as well, and the stronger binding of the D-amino acid hybrid bearing complexes was found over the complexes with L-amino acids. The UV-vis and CD spectroscopic studies suggested that coordination occurs between the metal center and nucleobases of ct-DNA. The HQCl-D-hPro ligand and its RhCp* and RuCym complexes do not induce DNA cleavage; therefore, it is suggested that these compounds do not act through DNA damage.

## Figures and Tables

**Table 1:** In vitro cytotoxicity expressed as IC_50_ values for the ligands (HQCl-D-Pro, HQCl-D-hPro) and their RhCp* and RuCym complexes in Colo-205, Colo-320 human colon adenocarcinoma and MRC-5 human lung fibroblast cell lines in comparison with HQCl-L-Pro. (Incubation time: 72 h).

IC_50_ (μM)	Colo-205	Colo-320	MRC-5
HQCl-L-Pro ^a^	20.92 ± 0.80	12.87 ± 0.74	4.57 ± 0.21
HQCl-D-Pro	17.0 ± 1.0	13.41 ± 0.79	5.33 ± 0.44
HQCl-D-hPro	18.9 ± 1.1	11.9 ± 1.5	3.63 ± 0.10
RhCp*–HQCl-D-Pro	31.5 ± 1.4	34.3 ± 1.0	82.1 ± 5.1
RhCp*–HQCl-D-hPro	21.54 ± 0.60	18.90 ± 0.54	37.6 ± 2.3
RuCym–HQCl-D-Pro	>100	>100	>100
RuCym–HQCl-D-hPro	>100	>100	>100
doxorubicin	0.05 ± 0.01	0.19 ± 0.02	0.82 ± 0.16

^a^ IC_50_ (Colo-205) = 23.4 μM, IC_50_ (Colo-320) = 8.5 μM [25].

**Table 2:** Proton dissociation constants (p*K*_a_) of the ligands. {*I* = 0.2 M KNO_3_; *T* = 25.0 °C}.

Method	Ligand	p*K*_a_ (N_q_H^+^)	p*K*_a_ (OH)	p*K*_a_ (N_hPro/Pro_H^+^)
pH-potentiometry	HQCl-D-Pro ^a^	< 2	7.71 ± 0.06	>11
	HQCl-D-hPro	2.42 ± 0.04	7.52 ± 0.06	>11
UV-vis	HQCl-D-Pro	2.13 ± 0.03	7.77 ± 0.04	>11
	HQCl-D-hPro	2.43 ± 0.01	7.49 ± 0.04	11.43 ± 0.01
^1^H NMR	HQCl-D-Pro	2.32 ± 0.03	7.75 ± 0.02	>11
	HQCl-D-hPro	2.52 ± 0.03	7.54 ± 0.02	>11

^a^ Values determined for HQCl-L-Pro: p*K*_a_ (N_q_H^+^): 2.36; p*K*_a_ (OH): 7.76; p*K*_a_ (NH^+^_Pro_): >11.5 [25].

**Table 3:** Proton dissociation constants (p*K*_a_) and conditional H_2_O/Cl^−^ exchange constants (log*K′* (H_2_O/Cl^−^) of the complexes (see equilibria in Scheme S1). {*I* = 0.2 M KNO_3_; *T* = 25.0 °C}.

Complex	Method	p*K*_a1_	p*K*_a2_	log*K*′ (H_2_O/Cl^−^)
RhCp*–HQCl-D-Pro	UV-vis	10.08 ± 0.02	–	1.72 ± 0.01
RhCp*–HQCl-D-hPro	UV-vis	10.11 ± 0.02	–	1.79 ± 0.01
	^1^H NMR	9.82 ± 0.13 ^a^	10.06 ± 0.06 ^b^	–
RuCym–HQCl-D-Pro	UV-vis	8.96 ± 0.01	10.67 ± 0.01	1.18 ± 0.01
	^1^H NMR	9.02 ± 0.07	10.69 ± 0.06	–
RuCym–HQCl-D-hPro	UV-vis	8.78 ± 0.01	10.03 ± 0.02	1.28 ± 0.01
	^1^H NMR	8.77 ± 0.05	10.13 ± 0.04	–

^a^ Calculated from the chemical shifts of the C^15^H proton of hPro. ^b^ Calculated from the chemical shifts of the C^4^H proton of 8-hydroxyquinoline moiety.

**Table 4:** Conditional formation constant (log*K*_FHA_*′*) for the formation of the ternary complex with FHA determined by ^1^H NMR spectroscopy {*c*_FHA_ = 0.5 or 1 mM, c_complex_ = 0.5 or 1 mM, pH = 7.4, (PBS’); 10% (*v*/*v*) D_2_O}, Trp-quenching (log*K*_Q_*′*) and DG marker displacement (log*K*_DG_*′*) constants of the complexes for their binding to HSA at site I and II, respectively, obtained from spectrofluorometric measurements. {*c*_HSA_ = 1 µM, *c*_DG_ = 1 µM, c_complex_ = 0–40 µM, λ_EX_ = 295 nm (Trp-214) or λ_EX_ = 335 nm (DG); pH = 7.4, (PBS’)}.

	FHA log*K*_FHA_′	Trp-214 Quenching log*K*_Q_′, Site I	DG Displacement log*K*_DG_′, Site II
RhCp*–HQCl-L-Pro ^a^	4.7	5.5	5.6
RuCym–HQCl-L-Pro ^a^	5.1	5.7	6.1
RhCp*–HQCl-D-Pro	4.4 ± 0.2	5.6 ± 0.1	5.5 ± 0.1
RuCym–HQCl-D-Pro	4.4 ± 0.3	5.5 ± 0.1	5.6 ± 0.1
RhCp*–HQCl-D-hPro	4.5 ± 0.2	5.6 ± 0.1	5.4 ± 0.1
RuCym–HQCl-D-hPro	4.6 ± 0.2	5.3 ± 0.1	5.7 ± 0.1

^a^ Data are taken from Ref. [34].

## Data Availability

Authors can confirm that all relevant data are included in the article.

## Supplementary Materials

The following are available online at https://www.mdpi.com/article/10.3390/ijms222011281/s1.
