# Supplementary material for: 8-Hydroxyquinoline-Amino Acid Hybrids and Their Half-Sandwich Rh and Ru Complexes: Synthesis, Anticancer Activities, Solution Chemistry and Interaction with Biomolecules
Source: Int J Mol Sci. 2021 Oct 19;22(20):11281. doi: 10.3390/ijms222011281 (PMC8570331; doi:10.3390/ijms222011281)
Supplement: Supplementary file 1 [file ijms-22-11281-s001.zip › ijms-1415512 supplementary proofed.pdf]

8-Hydroxyquinoline-amino acid hybrids and their half-sandwich Rh and Ru complexes: synthesis, anticancer activities, solution chemistry and interaction with biomolecules

Tamás Pivarcsik, Orsolya Dömötör, János P. Mészáros, Nóra V. May, Gabriella Spengler, Oszkár Csuvik, István Szatmári, Éva A. Enyedy

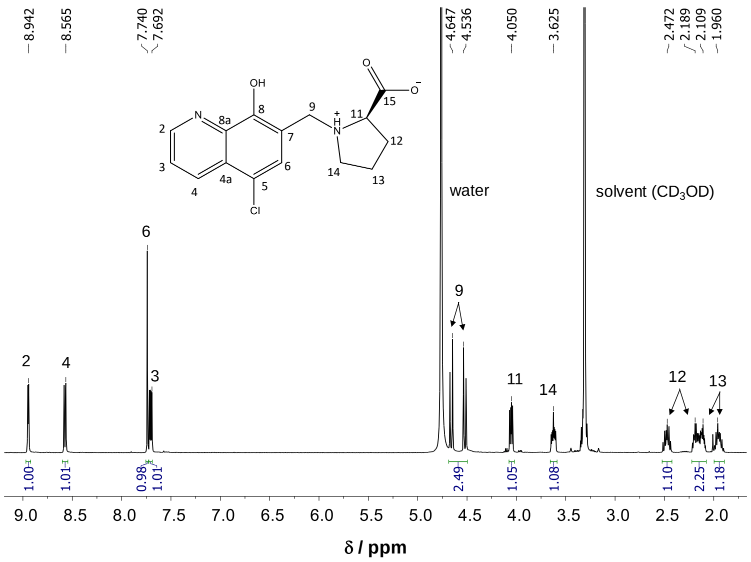

Figure S1. ^{1}H NMR spectrum of HQCl-D-Pro in CD_3OD. Inserted structure shows numbering of peaks. {$c_{HQCl\text{-}D\text{-}Pro}$ = 10 mM, T = 25.0°C}

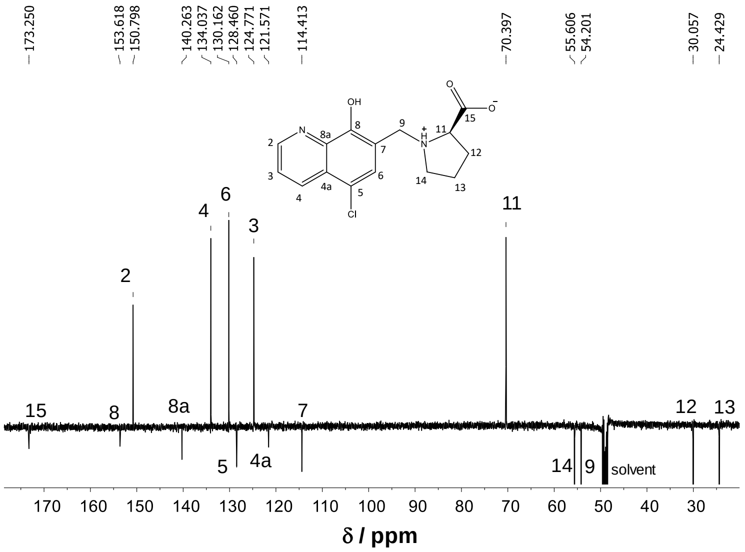

Figure S2. ^{13}C APT NMR spectrum of HQCl-D-Pro in CD_3OD. Attached proton test method: CH and CH_3 peaks are positive, C and CH_2 peaks are negative. Inserted structure shows numbering of peaks. {$c_{HQCl\text{-}D\text{-}Pro}$ = 10 mM, T = 25.0°C}

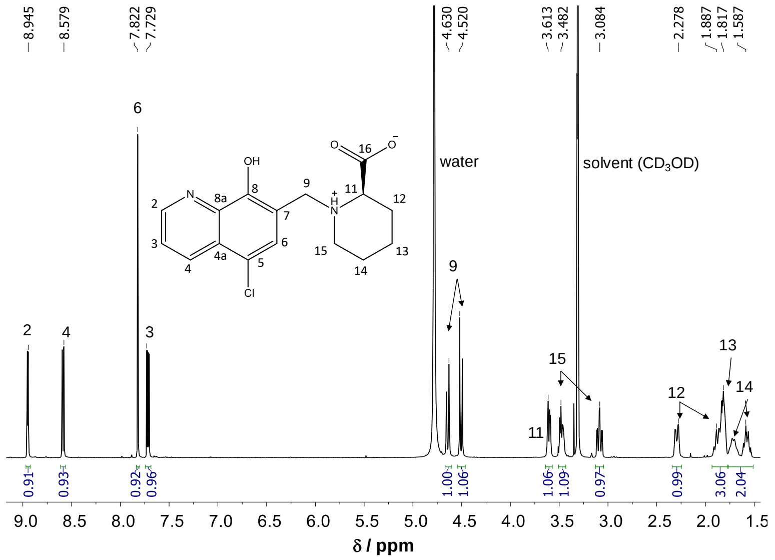

Figure S3. ^{1}H NMR spectrum of HQCl-D-hPro in CD_3OD. Inserted structure shows numbering of peaks. {$c_{\text{HQCl-D-hPro}}$ = 10 mM, T = 25.0°C}

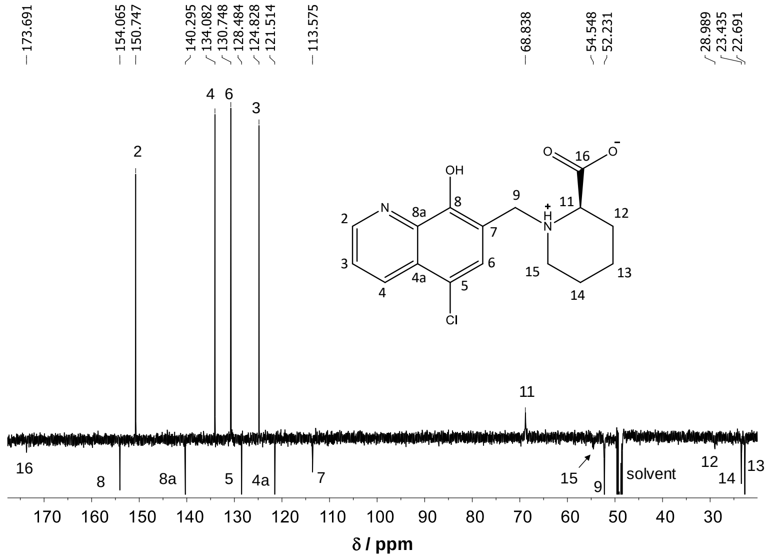

Figure S4. ^{13}C APT NMR spectrum of HQCl-D-hPro in CD_3OD. Attached proton test method: CH and CH_3 peaks are positive, C and CH_2 peaks are negative. Inserted structure shows numbering of peaks. {$c_{\text{HQCl-D-hPro}}$ = 10 mM, T = 25.0°C}

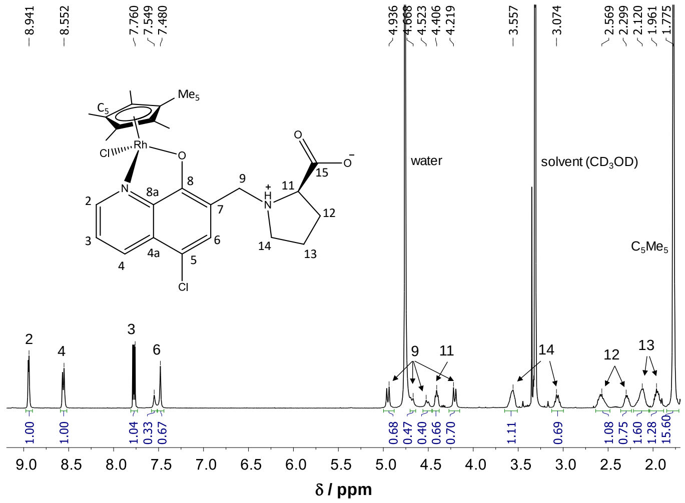

Figure S5. ^{1}H NMR spectrum of [Rh(η^5-C_5Me_5)(HQCl-D-Pro)Cl]Cl in CD_3OD. Inserted structure shows numbering of peaks. {$c_{complex}$ = 10 mM, T = 25.0°C}

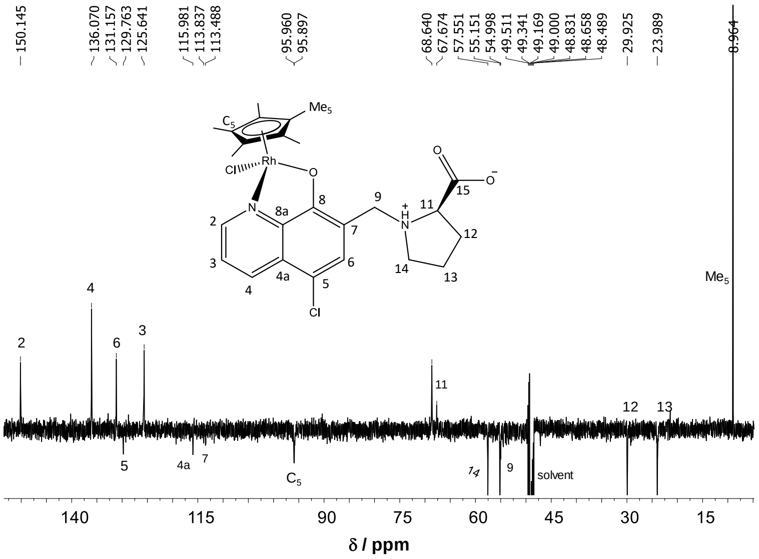

Figure S6. ^{13}C APT NMR spectrum of [Rh(η^5-C_5Me_5)(HQCl-D-Pro)Cl]Cl in CD_3OD. Attached proton test method: CH and CH_3 peaks are positive, C and CH_2 peaks are negative. Inserted structure shows numbering of peaks. {$c_{complex}$ = 10 mM, T = 25.0°C}

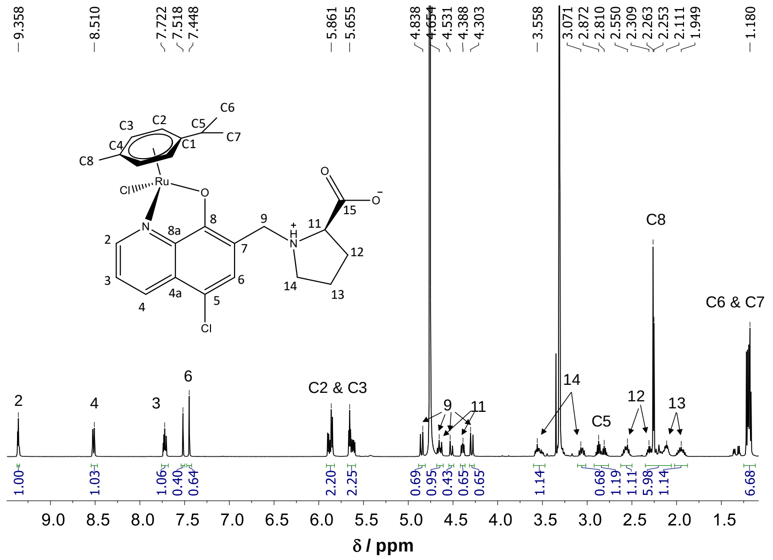

Figure S7. ^{1}H NMR spectrum of [Ru(η^{6}-*p*-cymene)(HQCl-D-Pro)Cl]Cl in CD_3OD. Inserted structure shows numbering of peaks. {$c_{complex}$ = 10 mM, T = 25.0°C}

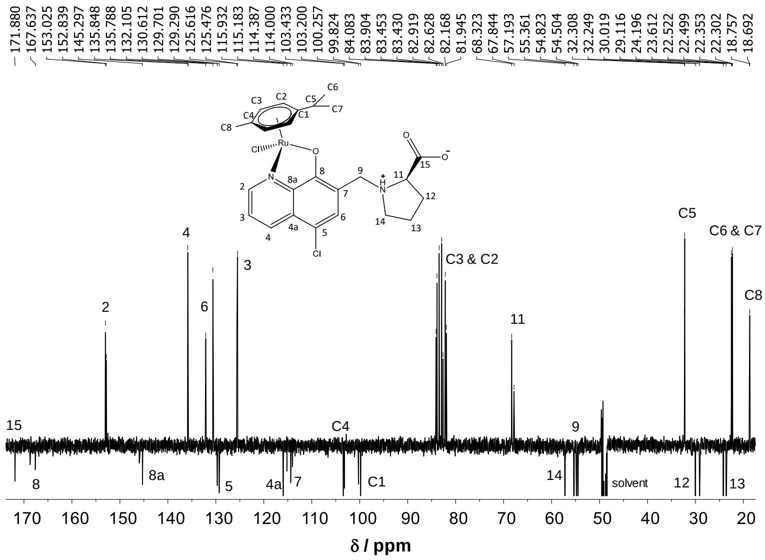

Figure S8. ^{13}C APT NMR spectrum of [Ru(η^{6}-*p*-cymene)(HQCl-D-Pro)Cl]Cl in CD_3OD. Attached proton test method: CH and CH_3 peaks are positive, C and CH_2 peaks are negative. Inserted structure shows numbering of peaks. {$c_{complex}$ = 10 mM, T = 25.0°C}

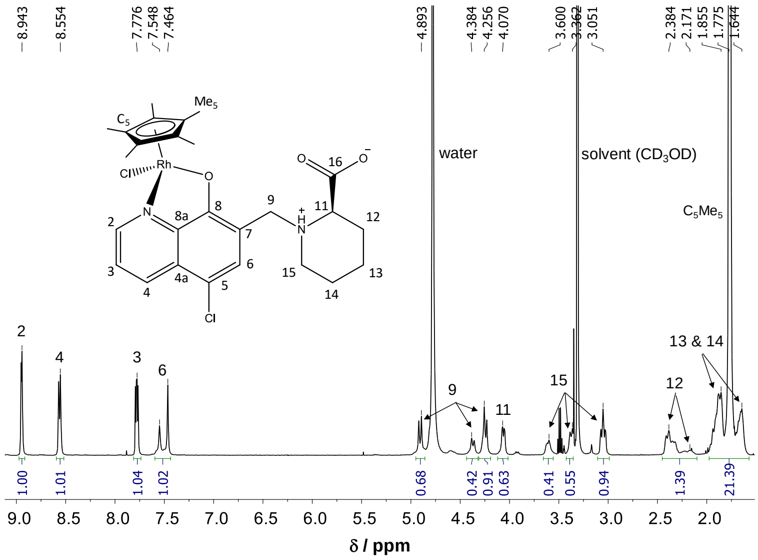

Figure S9. ^{1}H NMR spectrum of [Rh(η^5-C_5Me_5)(HQCl-D-hPro)Cl]Cl in CD_3OD. Inserted structure shows numbering of peaks. {$c_{complex}$ = 10 mM, T = 25.0°C}

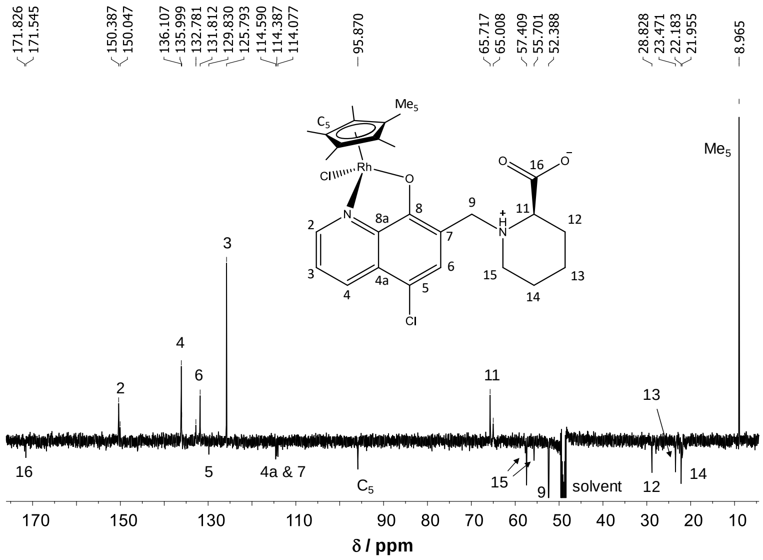

Figure S10. ^{13}C APT NMR spectrum of [Rh(η^5-C_5Me_5)(HQCl-D-hPro)Cl]Cl in CD_3OD. Attached proton test method: CH and CH_3 peaks are positive, C and CH_2 peaks are negative. Inserted structure shows numbering of peaks. {$c_{complex}$ = 10 mM, T = 25.0°C}

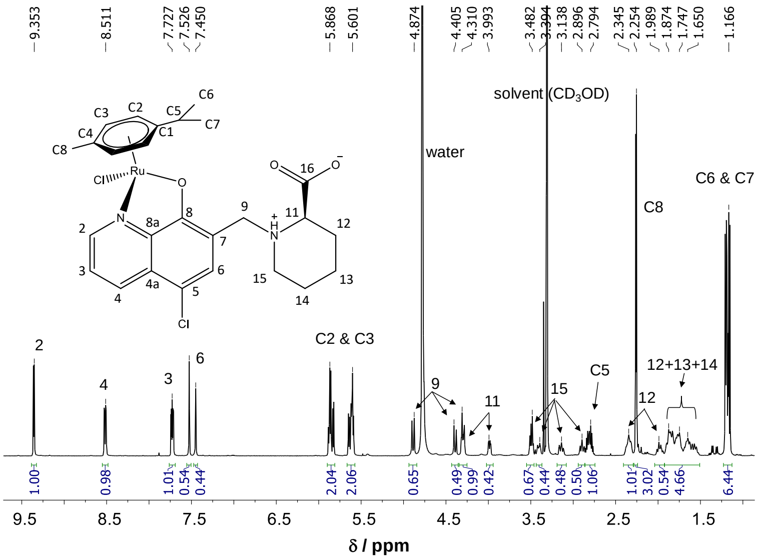

Figure S11. ^{1}H NMR spectrum of [Ru(η^6-*p*-cymene)(HQCl-D-hPro)Cl]Cl in CD_3OD. Inserted structure shows numbering of peaks. {$c_{complex}$ = 10 mM, T = 25.0°C}

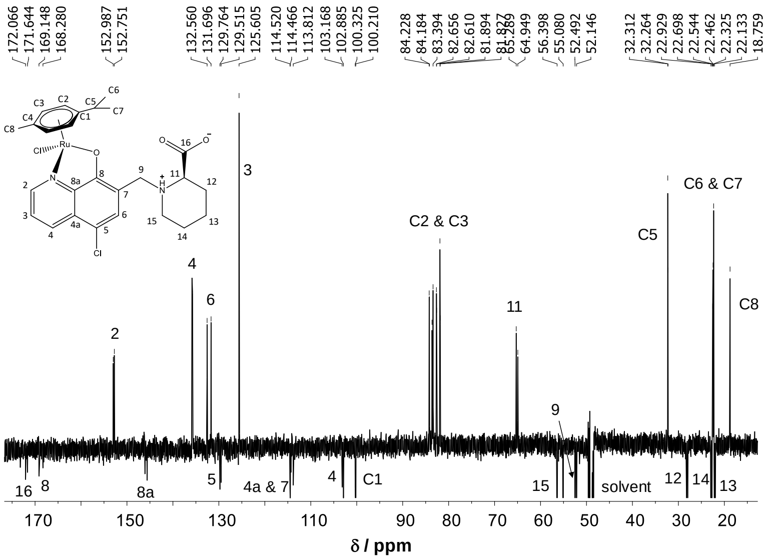

Figure S12. ^{13}C APT NMR spectrum of [Ru(η^6-*p*-cymene)(HQCl-D-hPro)Cl]Cl in CD_3OD. Attached proton test method: CH and CH_3 peaks are positive, C and CH_2 peaks are negative. Inserted structure shows numbering of peaks. {$c_{complex}$ = 10 mM, T = 25.0°C}

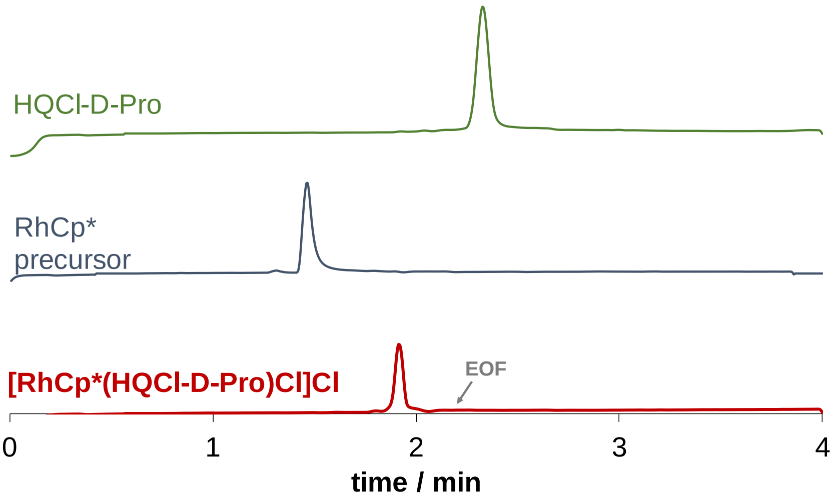

Figure S13. Electropherograms of the isolated complex [RhCp*(HQCl-D-Pro)Cl]Cl, the RhCp* precursor [RhCp*(μ-Cl)Cl]$_2$ and ligand HQCl-D-Pro at pH 7.4. {c = 500 μM; PBS'; λ = 200 nm}

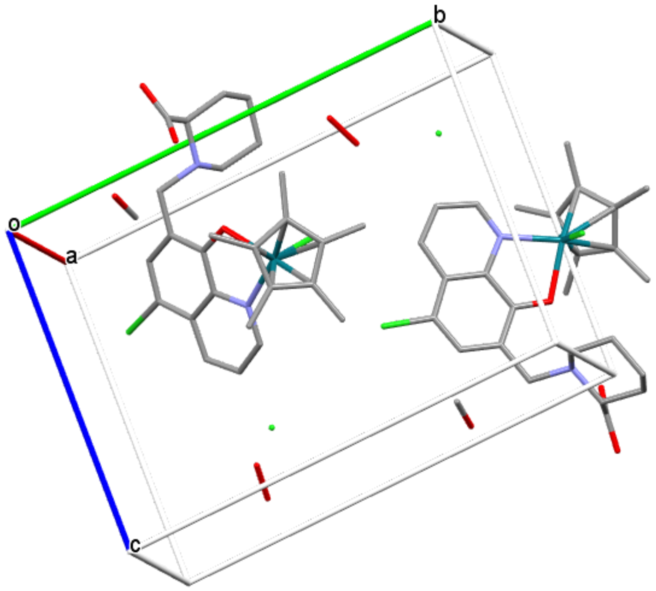

Figure S14. Unit cell containing two molecules in crystal (**1**).

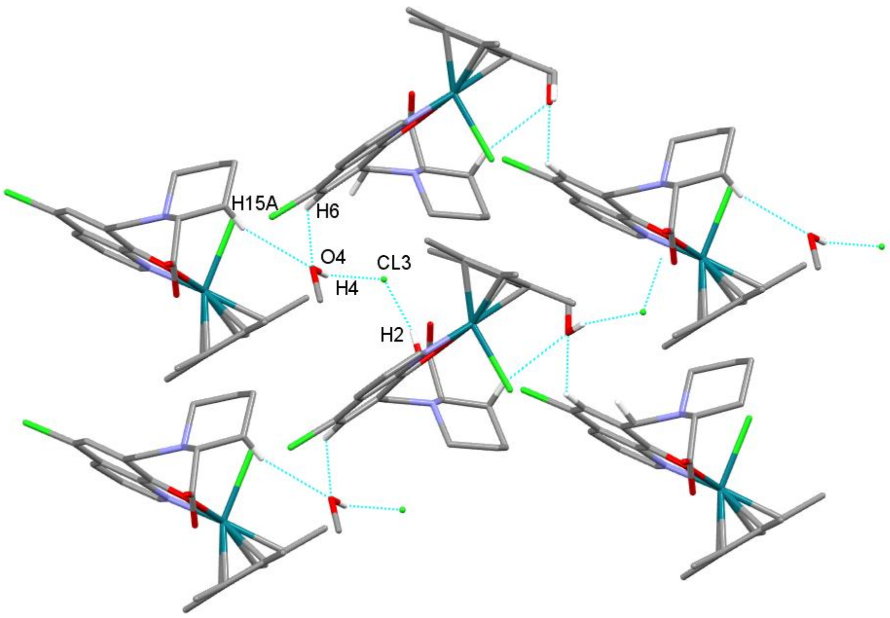

Figure S15. Packing arrangements in crystal (**1**) showing the C/O-H…O/Cl hydrogen bond interactions in the crystallographic direction 'b'.

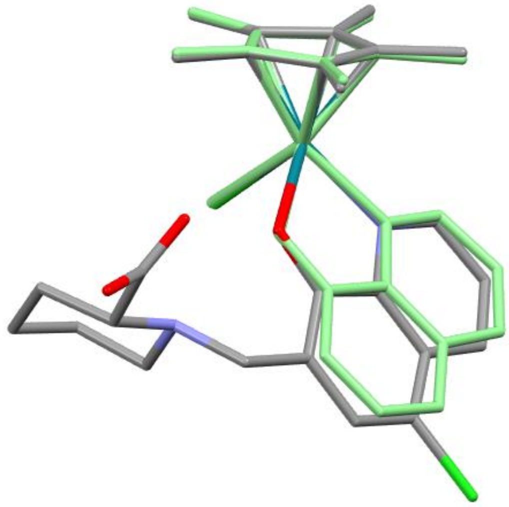

Figure S16. Comparison of the coordination geometry in crystal (**1**) (coloured by element) and [RhCp*(8-hydroxyquinolinato)Cl] Ref. code GAQLES [Dömötör, O.; Pape, V. F. S.; May, N. V.; Szakács, G.; Enyedy, É. A. Comparative solution equilibrium studies of antitumor ruthenium(η^6-*p*-cymene) and rhodium (η^5-C_5Me_5) complexes of 8-hydroxyquinolines. *Dalton Trans.* **2017**, 46, 4382-4392.] (green) with the overlay of Rh and the coordinating Cl, O and N atoms.

Table S1. Crystal data and structure refinement for [Rh(η^5-C_5Me_5)(HQCl-D-hPro)Cl]Cl·H_2O·CH_3OH (**1**).

Empirical formula	$C_{27}H_{36}Cl_3N_2O_5Rh$
Formula weight	677.84
Temperature	140(2) K
Radiation and wavelength	Mo-K_α, λ =0.71073Å
Crystal system	monoclinic
Space group	$P\,2_1$
Unit cell dimensions	a =7.6878(5)Å
	b =15.7291(10)Å
	c =12.9089(13)Å
	β =106.503(7)°
Volume	1496.7(2)$Å^3$
Z	2
Density (calculated)	1.504 Mg/m^3
Absorption coefficient, μ	0.876 mm^{-1}
F(000)	696
Crystal colour	yellow
Crystal description	platelet
Crystal size	0.45 x 0.15 x 0.15 mm
Absorption correction	numerical
Max. and min. transmission	0.909, 0.975
θ–range for data collection	3.052° ≤ θ ≤ 27.466°
Index ranges	$-9 \leq h \leq 9$;$-20\leq k \leq 20$;$-16 \leq l \leq 16$
Reflections collected	45948
Completeness to 2θ	0.998
Absolute structure parameter	0.034(14)
Friedel coverage	0.929
Friedel fraction max.	0.995
Friedel fraction full	0.999
Independent reflections	6809 [R(int) =0.0923]
Reflections I>2σ(I)	5846
Refinement method	full-matrix least-squares on F^2
Data / restraints / parameters	6809 / 1 /361
Goodness-of-fit on F2	0.994
Final R indices [I>2σ(I)]	R1 =0.0397, wR2 =0.0688
R indices (all data)	R1 =0.0524, wR2 =0.0717
Max. and mean shift/esd	0.000; 0.000
Largest diff. peak and hole	0.674; -0.783 e.$Å^{-3}$

Table S2. Selected bond lengths (Å) and angles (°) in crystal (**1**).

**Bond**	**Distance (Å)**	**Bond**	**Angle (°)**
Rh1-O1	2.104(4)	O1-Rh1-N1	78.7(2)
Rh1-C23	2.126(6)	O1-Rh1-Cl2	86.0(2)
Rh1-C21	2.132(6)	N1-Rh1-Cl2	87.4(14)
Rh1-C22	2.153(6)	Cl2-Rh1-Cg	128.14(11)
Rh1-N1	2.118(5)	N1-Rh-Cg	134.13(17)
Rh1-C24	2.129(5)	O1-Rh-Cg	125.37(14)
Rh1-C20	2.148(5)	C8-C7-C10-N2	43.1(4)
Rh1-Cl2	2.398(2)	Cp* - HQ[b]	57.6(4)
Rh-Cg[a]	1.758(3)	HQ - piperidine[b]	72.5(4)

[a] Cg is the centre of gravity of the Cp* ring. [b] Dihedral angle calculated between two ring planes.

Table S3. Hydrogen-bond geometry of crystal (**1**).

**D-H…A**	**D-H** (Å)	**H…A**(Å)	**D…A**(Å)	**D-H…A** (°)	**symmetry codes**
C11-H2A…O1	0.82	2.02	2.672(6)	131	intra
O2-H2…Cl3	0.82	2.16	2.953(5)	164	-1+x,y,z
O4-H4…Cl3	0.82	2.37	3.149(7)	167	
C6-H6…O4	0.93	2.52	3.283(9)	140	-1+x,y,z
C10-H10B…O5B	0.97	2.30	3.15(4)	146	-x,1/2+y,-z
C12-H12B…Cl3	0.97	2.76	3.650(8)	152	-1+x,y,z
C13-H13B…O5B	0.97	2.57	3.33(4)	135	
C15-H15A…O4	0.97	2.58	3.423(11)	145	1-x,-1/2+y,-z
C25-H25B…Cl3	0.96	2.80	3.705(8)	157	x,y,1+z
C29-H29C…Cl2	0.96	2.73	3.531(8)	141	1+x,y,z

Table S4. Gated events (%) in Colo-205 cells measured by flow cytometry. {3 h induction time}

name	concentration	gated events %		
		early apoptosis %	late apoptosis and necrosis %	cell death %
control: M627[a]	10 µM	5.27	16.8	15.2
control: M627[a]	20 µM	21.7	36.2	20.9
control: cisplatin	15 µM	1.06	4.70	3.18
HQCl-D-hPro	25 µM	0.987	9.10	27.5
RhCp*–HQCl-D-hPro	25 µM	0.838	7.50	20.7
RuCym–HQCl-D-Pro	25 µM	0.288	5.37	27.9

[a] M627: 12*H*-benzo[α]phenothiazine.

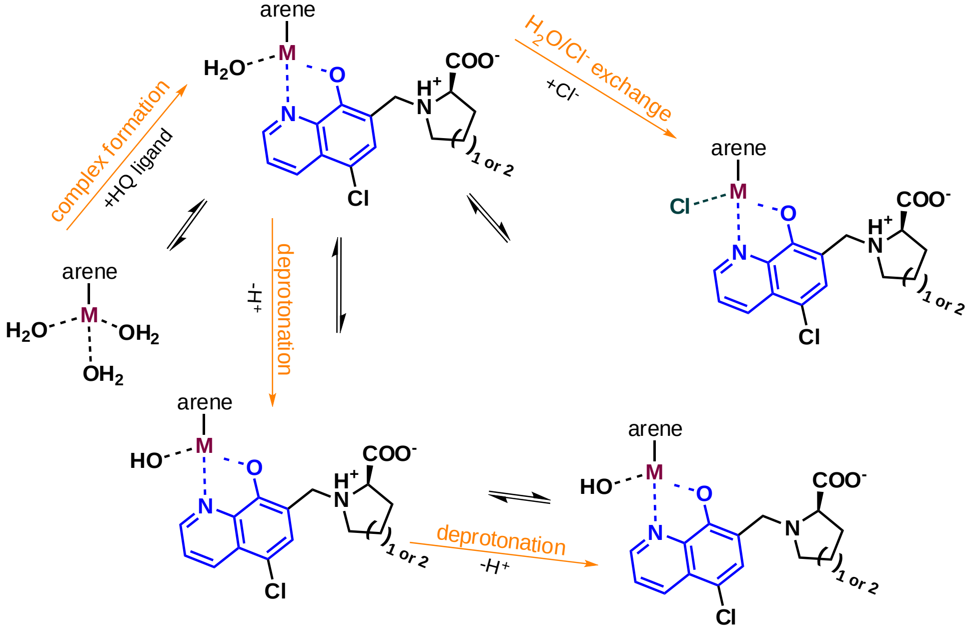

Scheme S1. Equilibrium processes for the formation, deprotonation and water/chloride co-ligand exchange of the RhCp* and RuCym complexes of the amino acid-8-hydroxyquinoline hybrids.

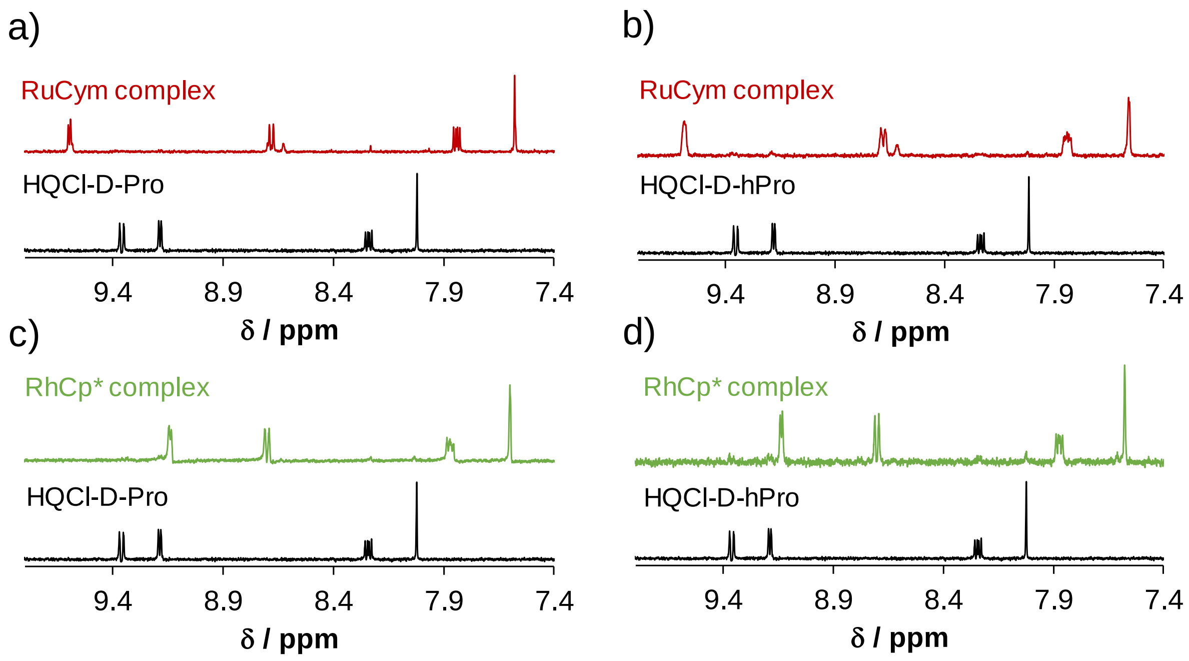

Figure S17. ^{1}H NMR spectra of a) [RuCym(HQCl-D-Pro)Cl]Cl, b) [RuCym(HQCl-D-hPro)Cl]Cl, c) [RhCp*(HQCl-D-Pro)Cl]Cl and d) [RhCp*(HQCl-D-hPro)Cl]Cl at pH 0.70 in the low-field region in comparison to the spectra of the ligands. {c = 500 μM; 10% (v/v) D_2O; 24 h}

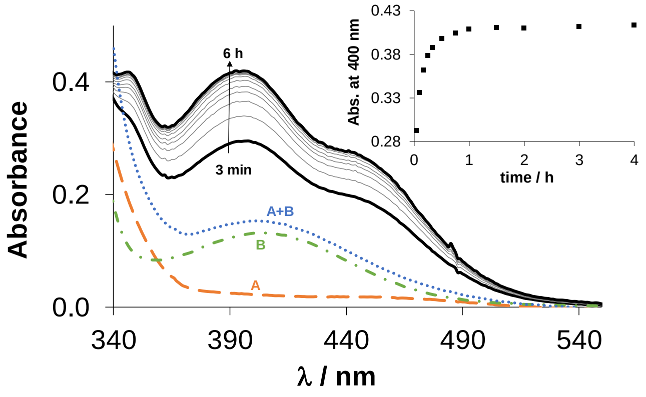

Figure S18. Time-dependent UV-vis spectra of the RuCym – HQCl-D-Pro (1:1) system (solid lines), spectra of $[RuCym(H_2O)_3]^{2+}$ (B, dash-dotted line), ligand (A, dashed line) and with their summed spectra (A+B, dotted line) at pH = 4.06. Inserted figure shows the absorbance changes at 400 nm plotted against the time. {c_{RuCym} = c_{ligand} = 120 μM; I = 0.2 M KNO_3; pH = 4.06; T = 25.0 °C; ℓ = 1 cm}

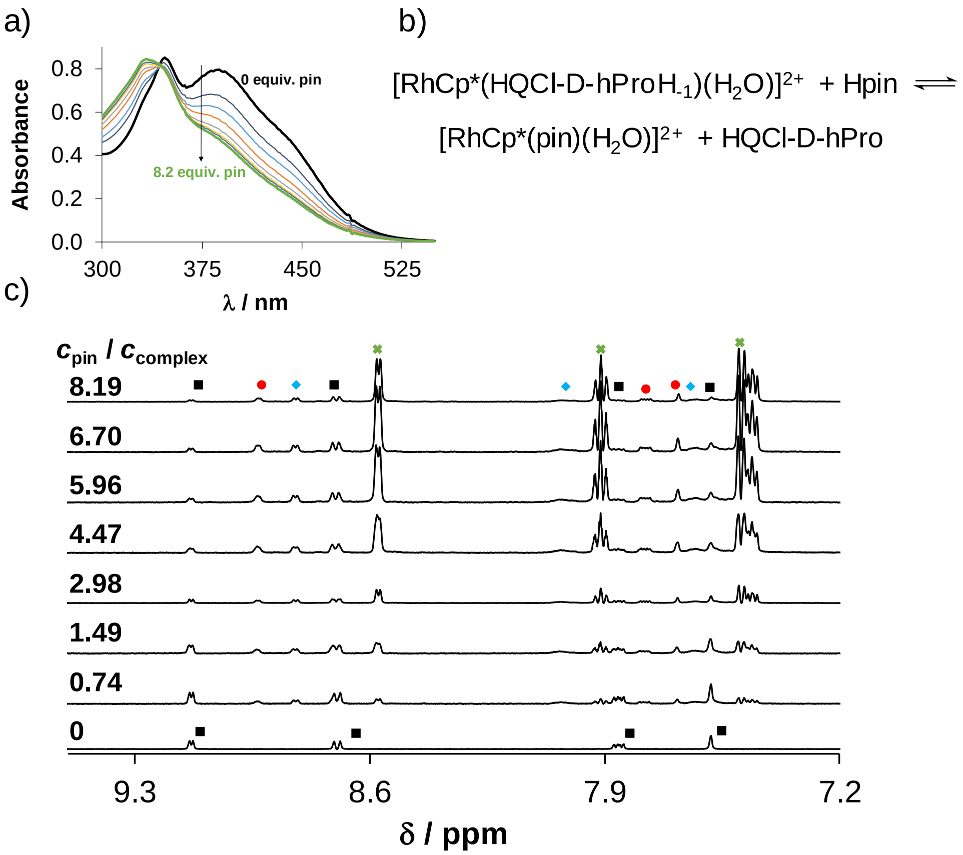

Figure S19. a) UV-vis spectra of RhCp* – HQCl-D-hPro – 2-picolylamine (pin) system at various pin-to-complex ratios in chloride ion free medium using 24 h equilibration time. b) Equilibrium process for the competition reaction. c) ^{1}H NMR spectra recorded for the same samples used for UV-vis experiments. Symbols: ■: $[RhCp^*(HQCl\text{-}D\text{-}hPro)(H_2O)]^{2+}$ complex, ●: free HQCl-D-hPro ligand, ♦: $[RhCp^*(pin)(H_2O)]^{2+}$ complex, ×: free pin. {c_{RhCp^*} = $c_{HQCl\text{-}D\text{-}hPro}$ =184 μM; c_{pin} = 0 – 1.522 mM; I = 0.2 M KNO_3; T = 25.0 °C; 10% (v/v) D_2O; pH = 7.4 (20 mM phosphate buffer); ℓ = 1 cm}

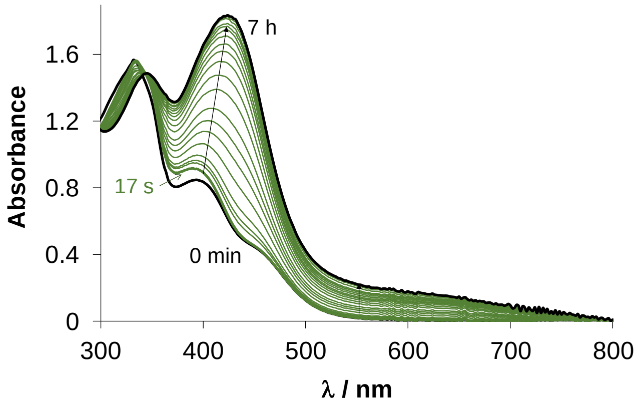

Figure S20. Time-dependent UV-vis spectra of the RuCym–HQCl-D-Pro complex in the presence of 2 equiv. HQCl-D-Pro at pH = 7.40. {$c_{complex}$ = 200 μM; $c_{HQCl\text{-}D\text{-}Pro}$ = 400 μM; I = 0.2 M KNO_3; pH = 7.40 (20 mM phosphate buffer); T = 25.0 °C; ℓ = 1 cm

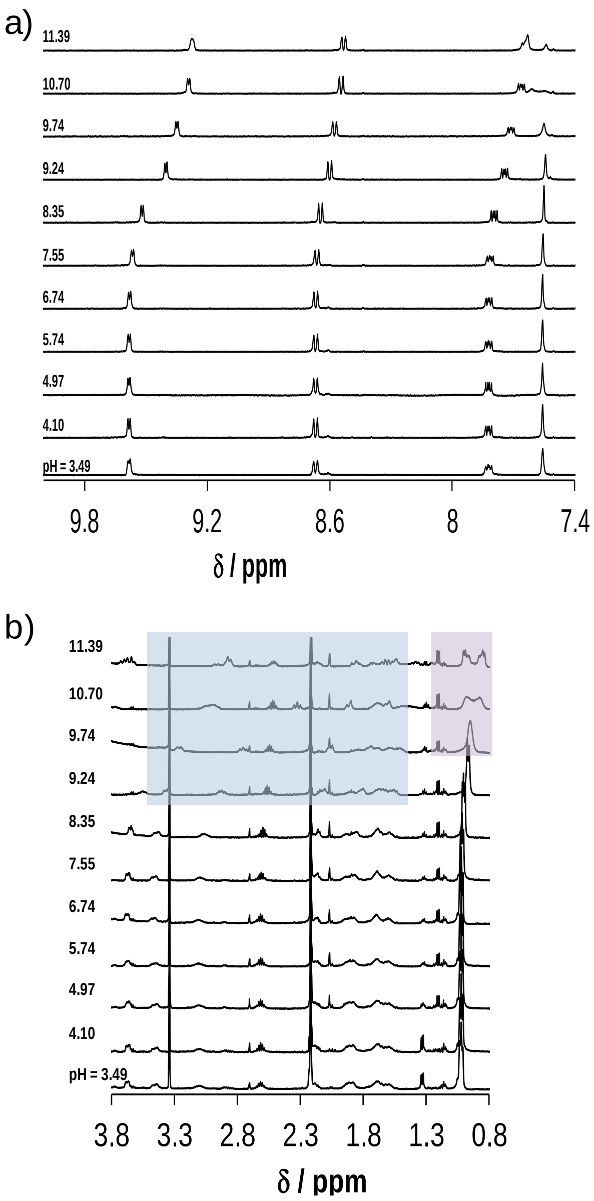

Figure S21. ^{1}H NMR spectra of the RuCym – HQCl-D-Pro (1:1) system at various pH values in the a) high-field and the b) low-field regions. Peaks in a) belong to the aromatic CH protons of the 8-hydroxyquinoline scaffold. Peaks in b) in the blue box show the changes of the proline CH_2 protons, and yellow box indicate the loss of symmetry of the *p*-cymene CH_3 groups. {$c_{RuCym} = c_{ligand}$ = 500 μM; I = 0.2 M KNO_3; 10% (v/v) D_2O; T = 25.0 °C}

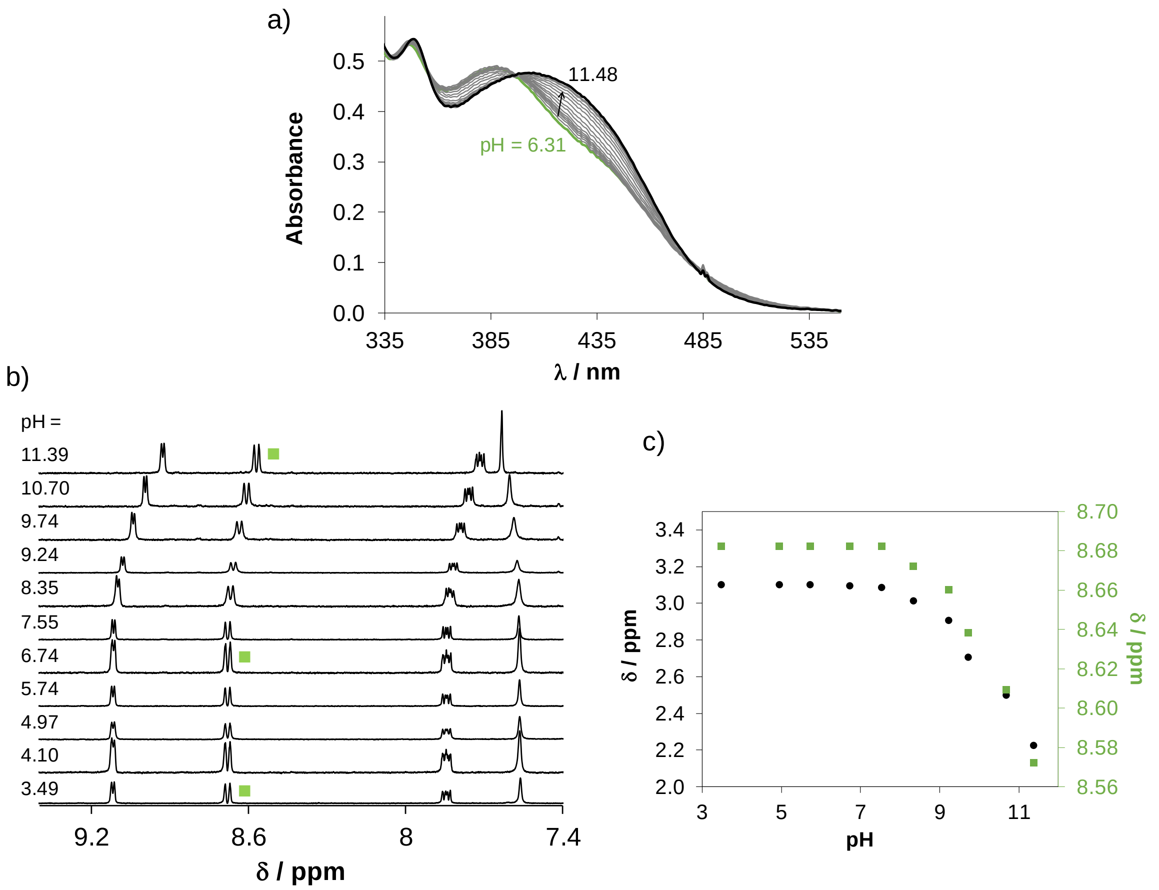

Figure S22. a) UV-vis spectra of the RhCp* – HQCl-D-hPro (1:1) system at various pH values. b). ^{1}H NMR spectra at various pH values in the high-field region, and c) chemical shifts of C^{4}H of the 8-hydroxyquinoline scaffold (■) and C^{15}H of the hPro moiety. {c_{RhCp*} = c_{ligand} = 111 μM (UV-vis; ℓ = 1 cm) or 500 μM (NMR, with 10% (v/v) D_2O); I = 0.2 M KNO_3; T = 25.0 °C}

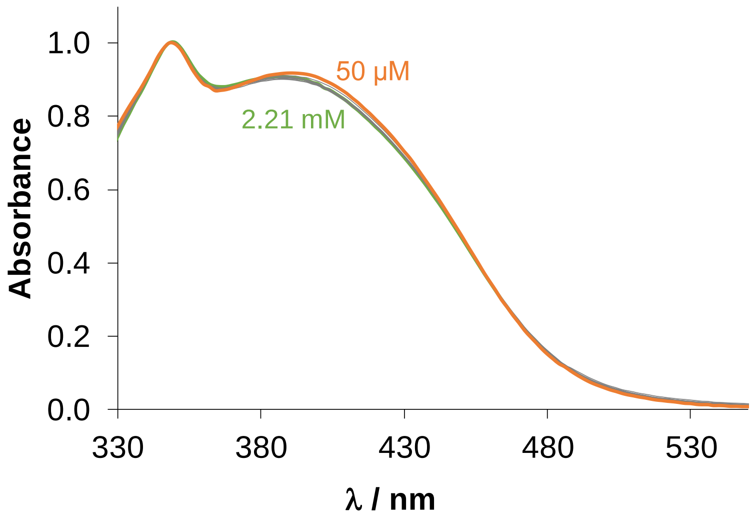

Figure S23. Normalized UV-vis spectra of the RhCp* – HQCl-D-Pro complex measured at various concentrations and path lengths. {c_{RhCp*} = c_{ligand} = 50 – 2210 μM; ℓ = 1 – 50 mm; pH = 11.0; T = 25.0 °C}

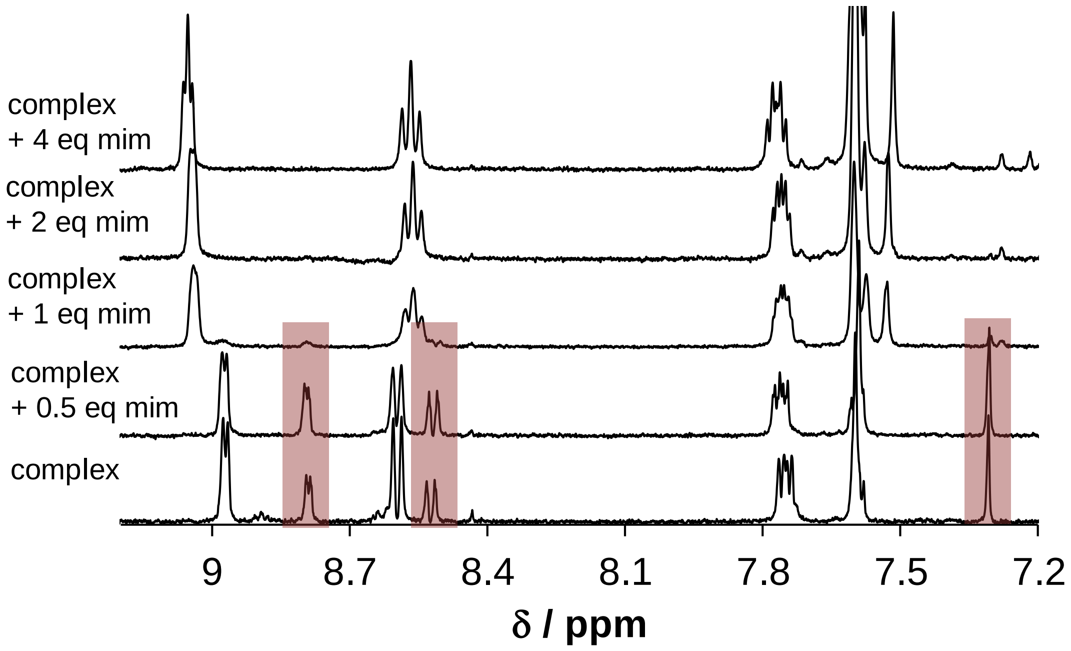

Figure S24. ^{1}H NMR spectra of the RhCp*–HQCl-D-Pro complex with various equivalents of *N*-methylimidazole (mim). Red boxes indicate the peaks assigned to the dimeric RhCp* – HQCl-D-Pro species. {c_{RhCp*} = c_{ligand} = 500 µM (NMR, with 10% (v/v) D_2O); pH = 11.0; *I* = 0.2 M KNO_3; *T* = 25.0 °C}

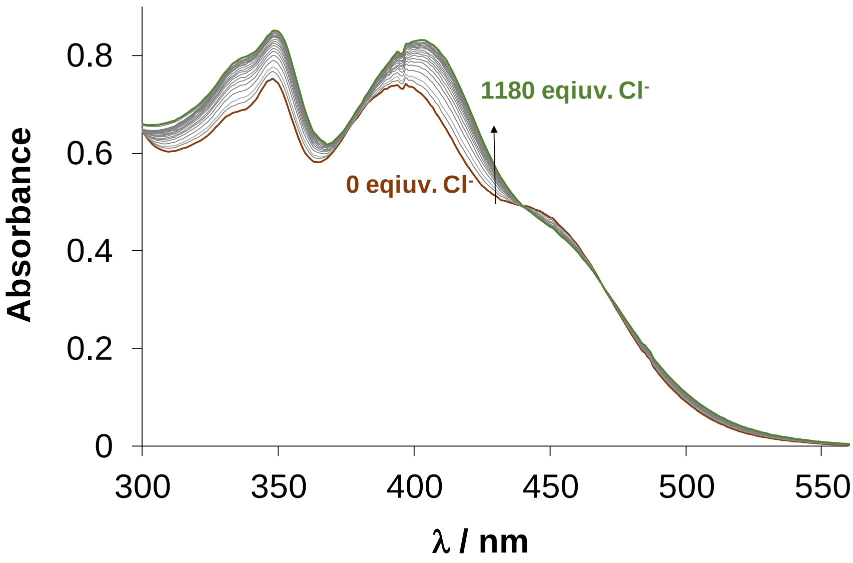

Figure S25. UV-vis spectra of the RhCp*–HQCl-D-hPro complex at pH 7.4 measured at various chloride ion concentrations. {c_{RhCp*} = c_{ligand} = 210 µM; ℓ = 1 cm; pH = 7.4 (20 mM phosphate buffer); *T* = 25.0 °C}

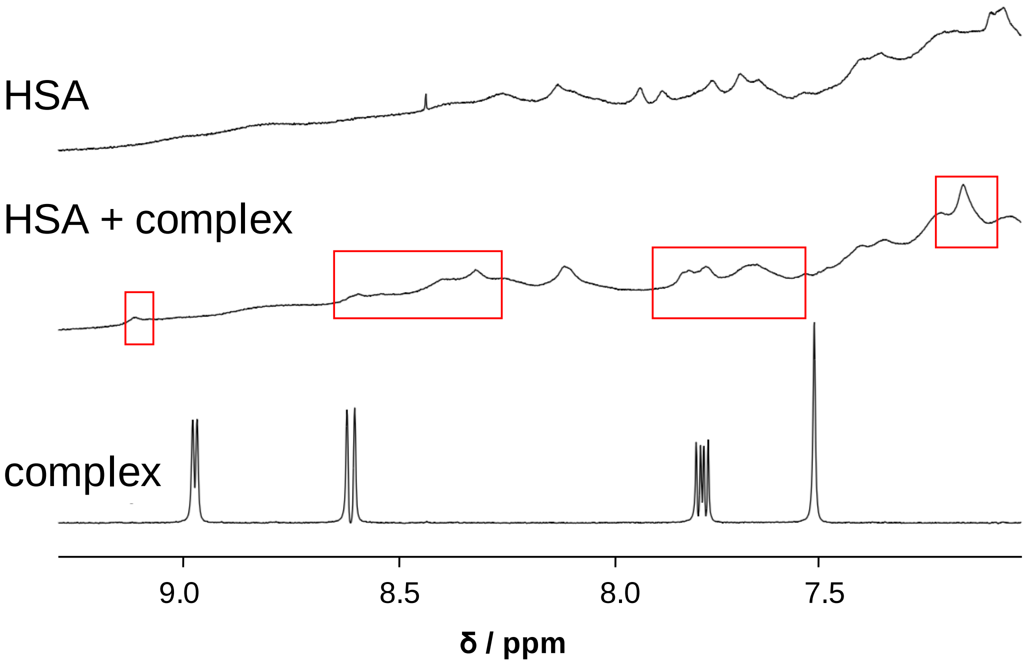

Figure S26. ^{1}H NMR spectra of HSA, the RhCp*–HQCl-D-Pro complex and their mixtures. Red boxes indicate the peaks assigned to the bound complexes. {$c_{complex}$ = 1 mM; c_{HSA} = 0.5 mM; 10% (v/v) D_2O); pH = 7.4 (PBS'); T = 25.0 °C}

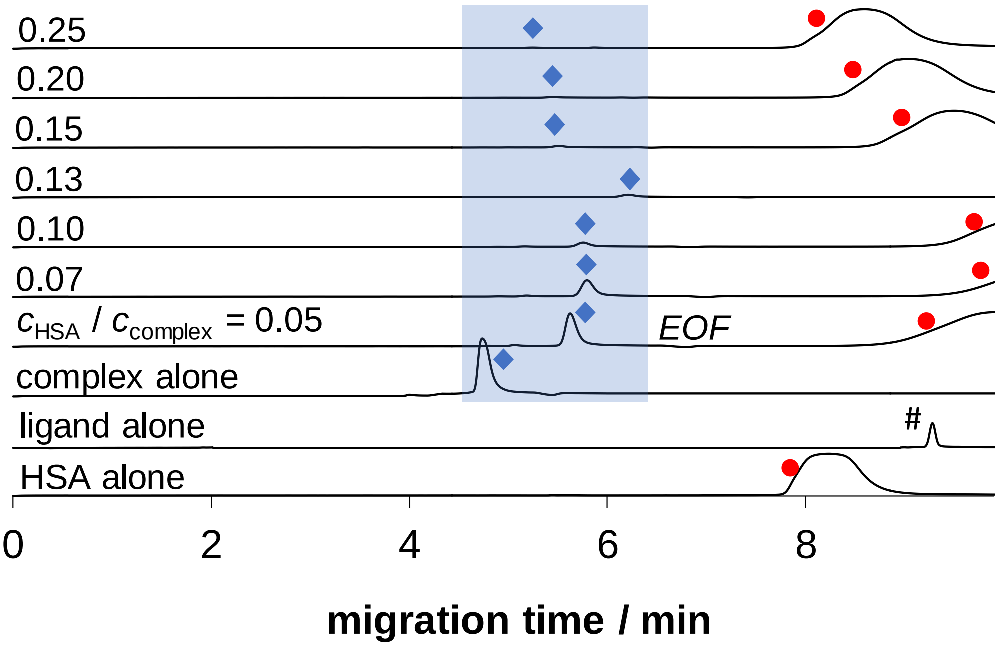

Figure S27. Electropherograms of HSA alone and in the presence of various equivalents of RuCym–HQCl-D-Pro complex (0.05 – 0.25). Symbols are indicating the free complex (♦), HSA & HSA-complex adduct (●), free ligand (#) and electroosmotic flow (EOF). Migration time of the free complex is in the range of 5.6 – 6.2 min, and peaks belonging to the free complex were assigned based on their UV-vis spectra measured at the peak maxima. {c_{HSA} = 50 µM, $c_{complex}$ = 250 – 1000 µM; pH = 7.40 (PBS' buffer); λ = 200 nm; T = 25.0 °C}

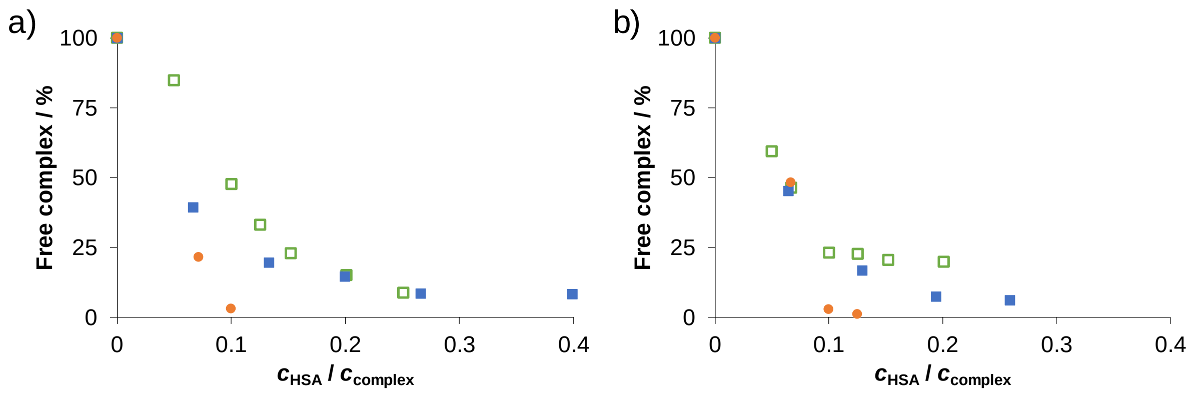

Figure S28. Molar fractions of the unbound a) RhCp* and b) RuCym complexes plotted against c_{HSA} / $c_{complex}$ ratio calculated on the basis of the CZE measurements. The concentration of the free complex was determined using external calibration. Symbols for the ligands in the complexes: HQCl-D-Pro (□), HQCl-L-Pro (■) and HQCl-D-hPro (●). {c_{HSA} = 50 µM, $c_{complex}$ = 250 – 1000 µM; pH = 7.40 (PBS' buffer)}

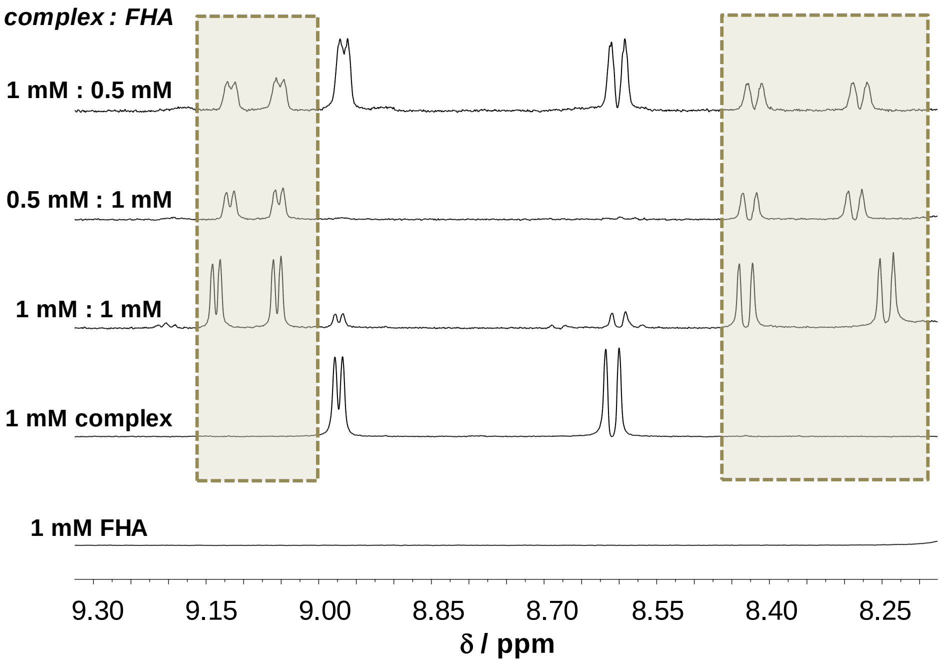

Figure S29. ^{1}H NMR spectra recorded for FHA, for the RhCp*–HQCl-D-hPro complex and their various mixtures. Framed boxes show the peaks of the forming ternary complex. {10% (v/v) D_2O; pH = 7.40 (PBS' buffer)}

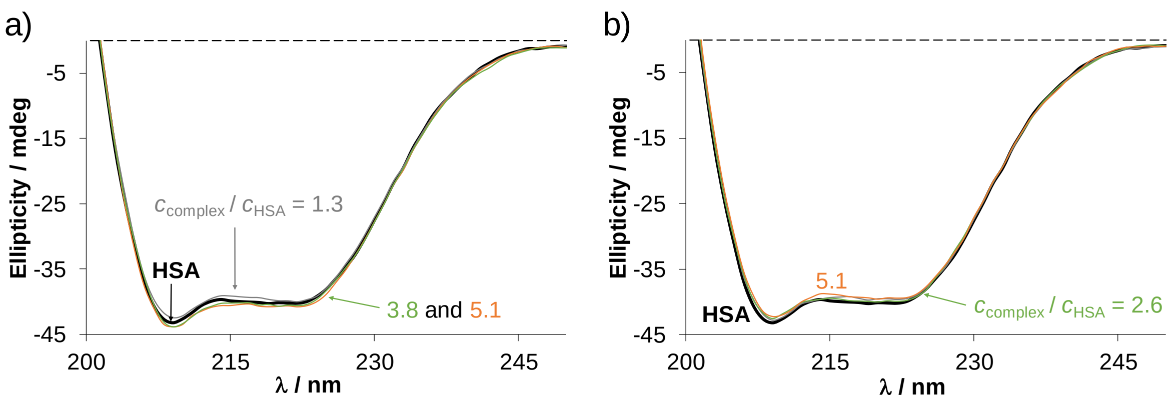

Figure S30. CD spectra of HSA in the absence and presence of various equiv. a) RhCp*–D-HQCl-hPro and b) RuCym–D-HQCl-hPro complex. The % α-helix content of HSA was decreased from 50% to 47% or 46% in the case of the RhCp* and RuCym complex, respectively based on the calculation suggested by Micsonai *et al.* [Micsonai, A.; Wien, F.; Kernya, L.; Lee, Y.-H.; Goto, Y.; Réfrégiers, M.; Kardos, J. Accurate secondary structure prediction and fold recognition for circular dichroism spectroscopy. *Proc. Natl. Acad. Sci.* **2015**, 112, E3095-E3103]. {$c_{complex}$ = 0 – 10.2 μM, c_{HSA} = 2 μM, pH = 7.40 (PBS' buffer); ℓ = 0.2 cm}

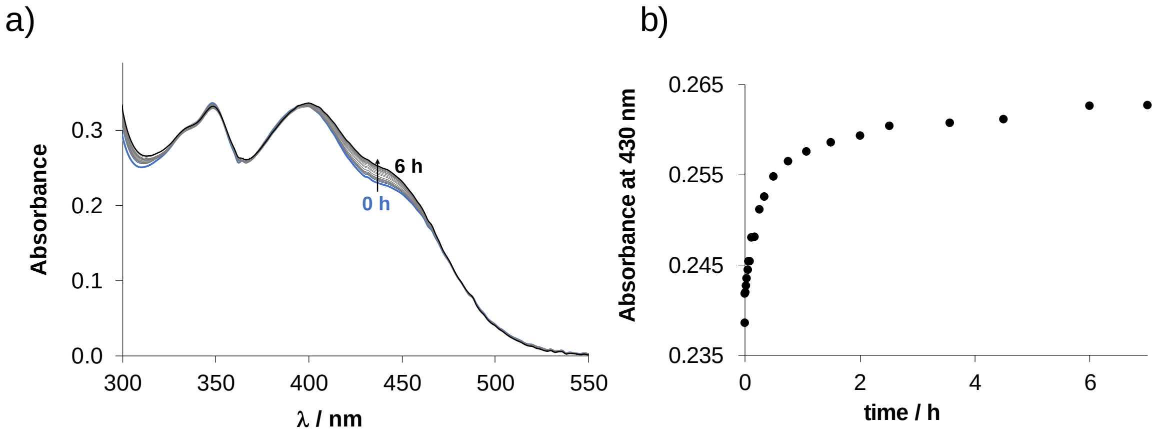

Figure S31. a) Time-dependent UV-vis spectra of RuCym-HQCl-D-hPro – ct-DNA system. b) Absorbance values at 430 nm as a function of time. {$c_{complex}$ = 100 μM, $c_{ct\text{-}DNA}$ = 200 μM, c_{KCl} = 4 mM; pH = 7.40 (20 mM phosphate buffer); ℓ = 1 cm}

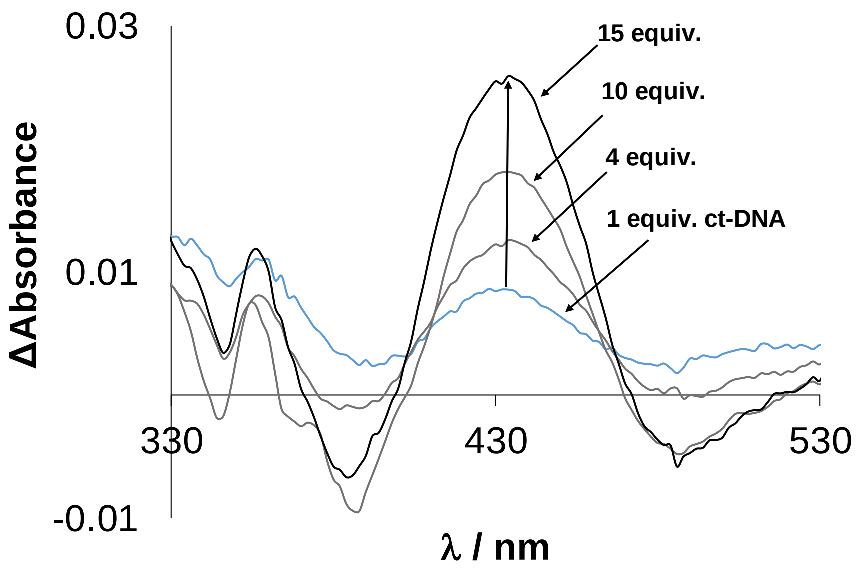

Figure S32. UV-vis difference spectra of RuCym–HQCl-L-Pro complex in the presence of various equiv. ct-DNA (difference spectrum = spectrum of the complex with ct-DNA – spectra of the complex and the ct-DNA alone). {$c_{complex}$ = 88 μM, $c_{ct\text{-}DNA}$ = 0 – 1.75 mM, c_{KCl} = 4 mM; pH = 7.40 (20 mM phosphate buffer); ℓ = 1 cm}

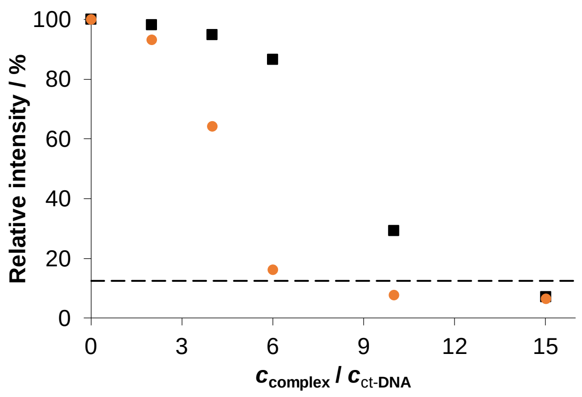

Figure S33. Relative fluorescence intensity of ct-DNA–EB followed at 605 nm for the complexes RhCp*–HQCl-D-hPro (●) and RuCym–HQCl-D-hPro (■). Dashed line denotes the relative intensity of the free EB. {$c_{ct\text{-}DNA}$ = 20 μM, c_{EB} = 5 μM, $c_{complex}$ = 0 – 300 μM, λ_{EX} = 510 nm, c_{KCl} = 4 mM; pH = 7.40 (20 mM phosphate buffer); ℓ = 1 cm}

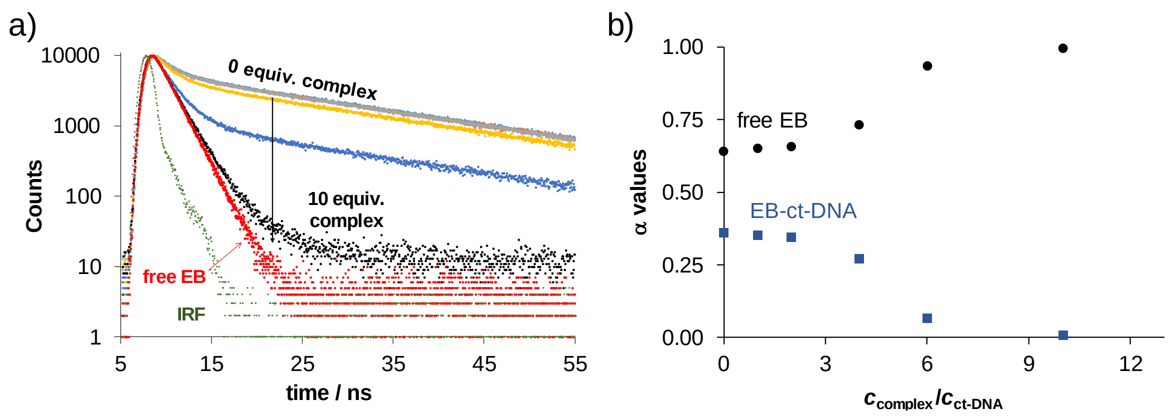

Figure S34. a) Fluorescence emission decay curves of ct-DNA – EB system at various equiv. RhCp*–HQCl-D-hPro, and instrument response function (IRF). b) Amplitudes (α) calculated at different $c_{complex}$ / c_{DNA} ratios for the RhCp*–HQCl-D-hPro – DNA – EB system. {$c_{ct\text{-}DNA}$ = 20 μM, c_{EB} = 5 μM, $c_{complex}$ = 0 – 300 μM, λ_{EX} = 460 nm, λ_{EM} = 610 nm; c_{KCl} = 4 mM; pH = 7.40 (20 mM phosphate buffer); ℓ = 1 cm}

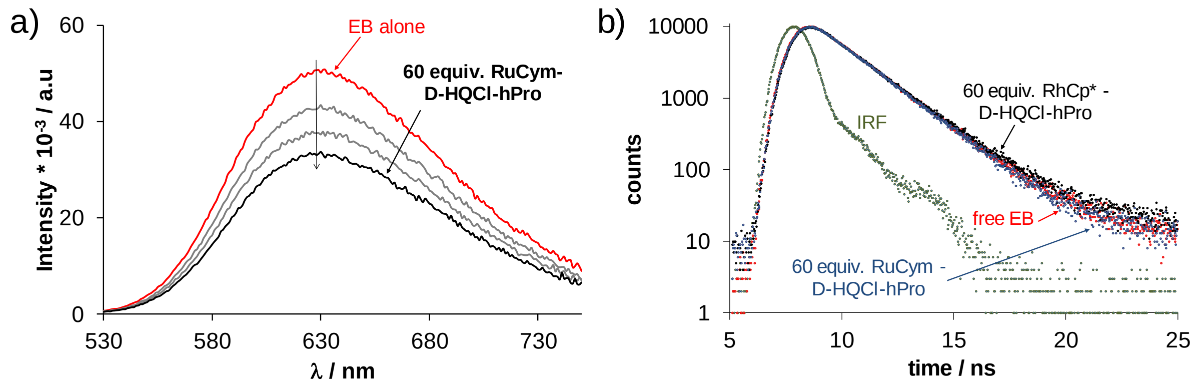

Figure S35. a) Fluorescence emission spectra of EB alone and in the presence of the RuCym–D-HQCl-hPro complex. b) Fluorescence emission decay curves of EB in the absence and presence of the indicated HQCl-D-hPro complexes and the instrument response function (IRF). {c_{EB} = 5 μM, $c_{complex}$ = 0 – 300 μM, λ_{EX} = 510 nm (steady-state), λ_{EX} = 460 nm, λ_{EM} = 610 nm (lifetime), c_{KCl} = 4 mM; pH = 7.40 (20 mM phosphate buffer); ℓ = 1 cm}

Table S5. Calculated fluorescence decay parameters [lifetime (τ_i) / ns; amplitude (α_i) and average lifetime (τ_{ave}) / ns] for complex–ct-DNA–EB systems in addition to fitting parameters (χ^2). {$c_{\text{ct-DNA}}$ = 20 µM, c_{EB} = 5 µM, c_{complex} = 0 – 300 µM, l = 1 cm, λ_{EX} = 460 nm, λ_{EM} = 610 nm, c_{KCl} = 4 mM; pH = 7.40 (20 mM phosphate buffer); ℓ = 1 cm}

		τ_1	τ_2	τ_{ave}	α_1	α_2	χ^2
	EB	1.56	-	1.56	1	-	1.26
	EB–ct-DNA	1.69	21.74	8.89	0.641	0.359	1.04
	$c_{\text{complex}}/c_{\text{ct-DNA}}$						
RhCp*–HQCl-D-hPro	1.00	1.67	21.76	8.69	0.651	0.349	1.07
	2.00	1.65	21.58	8.51	0.656	0.344	1.06
	4.01	1.64	21.44	6.96	0.731	0.269	1.08
	6.01	1.60	20.42	2.83	0.935	0.065	1.22[b]
	10.02	1.59	21.27[a]	1.69	0.995	0.005	1.23[b]
RuCym–HQCl-D-hPro	1.00	1.67	21.78	8.80	0.645	0.355	1.04
	2.00	1.63	21.53	8.47	0.656	0.344	1.05
	4.01	1.63	21.37	8.42	0.656	0.344	1.08
	6.01	1.66	21.15	8.17	0.666	0.334	1.08
	10.02	1.59	19.99	3.13	0.916	0.084	1.11
	15.03	1.57	-	1.57	1.000	0.000	1.23[b]

[a] It was kept constant.
[b] χ^2 values are considerably higher at the indicated complex/ct-DNA ratios. This phenomenon is due to the decrease of intercalated EB quantity, which results in relatively higher noise levels over the analyzed time window.
